# Photochromic Fentanyl Derivatives for Controlled μ‐Opioid Receptor Activation

**DOI:** 10.1002/chem.202201515

**Published:** 2022-09-12

**Authors:** Ranit Lahmy, Harald Hübner, Maximilian F. Schmidt, Daniel Lachmann, Peter Gmeiner, Burkhard König

**Affiliations:** ^1^ Institute of Organic Chemistry Department of Chemistry and Pharmacy University of Regensburg 93053 Regensburg Germany; ^2^ Department of Chemistry and Pharmacy Friedrich Alexander University 91052 Erlangen Germany

**Keywords:** arylazopyrazoles, fentanyl, mu-opioid receptor, G protein-coupled receptor, photopharmacology

## Abstract

Photoswitchable ligands as biological tools provide an opportunity to explore the kinetics and dynamics of the clinically relevant μ‐opioid receptor. These ligands can potentially activate or deactivate the receptor when desired by using light. Spatial and temporal control of biological activity allows for application in a diverse range of biological investigations. Photoswitchable ligands have been developed in this work, modelled on the known agonist fentanyl, with the aim of expanding the current “toolbox” of fentanyl photoswitchable ligands. In doing so, ligands have been developed that change geometry (isomerize) upon exposure to light, with varying photophysical and biochemical properties. This variation in properties could be valuable in further studying the functional significance of the μ‐opioid receptor.

## Graphical abstract

**Photoresponsive fentanyl‐based** ligands provide the opportunity to spatially, temporally, and non‐invasively regulate μ‐opioid receptor activity by using light. The repertoire of such ligands has been expanded in this work, yielding a desirable selection that differ in photophysical properties and/or metabotropic response.

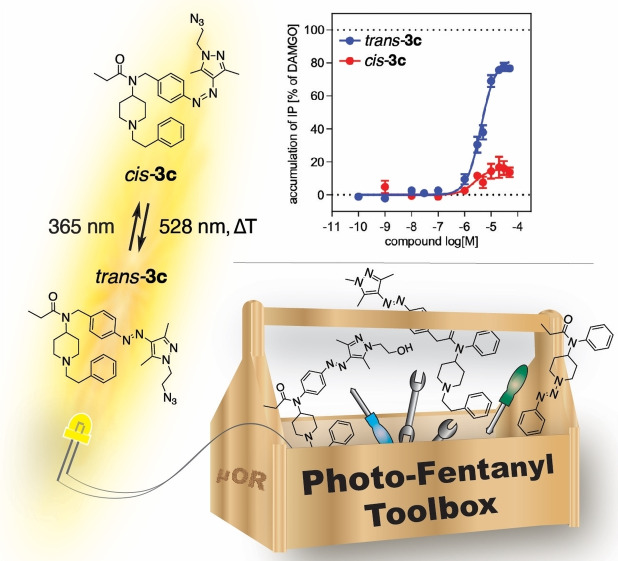

## Introduction

In recent years, the potent μ‐opioid receptor (μOR) agonist fentanyl has attracted significant media attention as a controversial medicine for the treatment of severe pain. Despite having advantageous analgesic properties, which are particularly useful in a clinical setting, this commercially available opioid also causes sedation and euphoria.^[1]^ The potency of fentanyl to induce these physiological effects has been linked to drug dependency and tolerance in clinical patients, which can ultimately lead to substance abuse with devastating consequences.[^1a^, ^2^] As a result, there has been an increasing demand to better understand the mechanism of the μOR and interacting opioid ligands. A better understanding of this complex system is important in the pursuit of physiologically‐biased opioids that solely induce the desired analgesic response, and not the unwanted side effects.^[3]^

The μOR is a G protein‐coupled receptor (GPCR), containing the characteristic seven‐transmembrane cellular domain with an extracellular N terminus and a cytoplasmic C terminus. Once agonists bind on the extracellular surface, the heterotrimeric G_i/o_ protein, which is specific to this class of GPCR, dissociates into G_αi_ and G_ßγ_ subunits. This induces intracellular transduction pathways, including the inhibition of cAMP production and the activation of G protein‐coupled inward‐rectifying potassium (GIRK) channels, that ultimately result in various physiological responses.[^3b^, ^4^] Current challenges in studying such GPCRs are their low expression levels in native cells, their flexibility and instability once purified from cell cultures, and their low affinity for their endogenous proteins.^[5]^ Advances in technology have been pivotal for the development of numerous selective and potent ligands that can be used as probe molecules to overcome some of these issues.[^5^, ^6^] These compounds have been implemented in a diverse range of chemical, biological, microscopic and spectroscopic techniques in order to further elucidate GPCR receptor signalling pathways and their resulting cellular responses.[^6^, ^7^]

Over the past few decades, there has been an increasing interest in using photochemical tools for such purposes.^[8]^ Despite being investigated as early as 1969, photoswitchable ligands have only recently received increasing attention and have been described as new‐age powerful biological tools.^[9]^ Photoswitchable ligands are composed of two main components: a selective bioactive small molecule and a photoswitchable moiety that has either been incorporated into or linked to the structure of the molecule. Upon exposure to light, the photoswitchable functionality undergoes a reversible change in structure and/or properties.[^9b^, ^10^] This change might result in a significant change in receptor affinity, thus resulting in compounds that have a biologically active and inactive state. The main advantage in developing such a photoswitchable ligand is that it could allow for spatial and temporal control of drug activity. Such examples have been documented, including those that are relevant to the GPCR field.^[11]^ A tool with this capability is useful to further understand receptor mechanism and signalling pathways through kinetic and dynamic studies.^[9b]^ For example, current limitations in the use of conventional probes are the inability to have a uniform start and stop time of receptor activation in biological and biochemical experiments.

**Figure 1:**
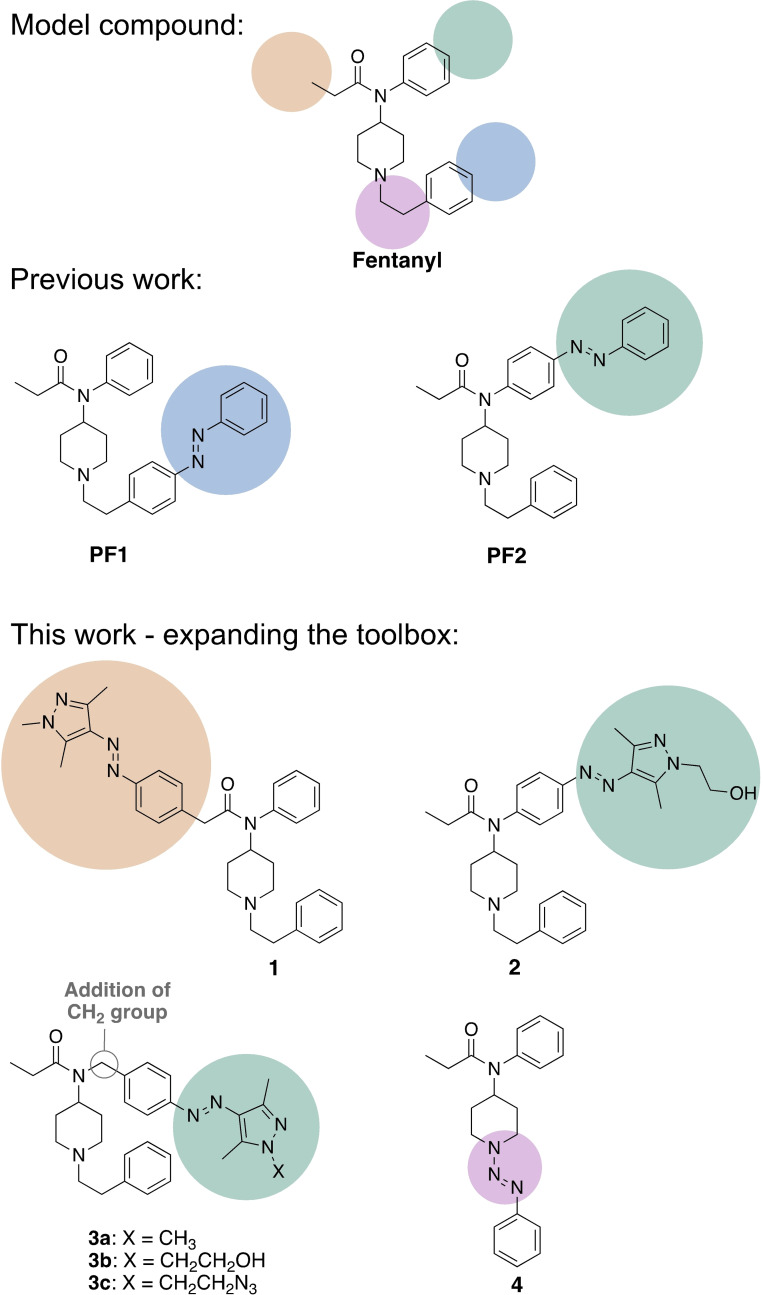
Structures of photoswitch‐containing ligands that target the μOR, modelled on the fentanyl pharmacophore (top). Previous work by Trauner et al.^[4b]^ attached the azobenzene photoswitch moiety to the pharmacophore in two positions (middle). Our work expands the biological “toolbox” of fentanyl photoswitchable ligands by attaching a reversibly switchable arylazopyrazole unit in various positions, or by incorporating triazene into the core structure of fentanyl (bottom).

The most explored class of photoswitchable ligands that undergoes a change in geometry are the azobenzenes.^[9b]^ This change in geometry is a result of a *trans*‐to‐*cis* isomerization upon light exposure. Azobenzenes and their derivatives can then revert to the more stable *trans* isomer either thermally or upon exposure with light of a different wavelength.^[12]^ A recent application of this was performed by Trauner et al., who developed the first fentanyl azobenzene photoswitches.^[4b]^ Incorporating azobenzene on the terminus of the phenethyl moiety to form photofentanyl 1 (**PF1**), diminished receptor agonism, however, incorporation on the phenyl propanamide unit to obtain photofentanyl 2 (**PF2**) provided a successful candidate (Figure 1). By monitoring potassium influx through GIRK channels, it was revealed that switching **PF2** to the *trans* isomer (irradiation with blue light) resulted in a potassium influx through the GIRK channels, while the *cis* isomer (irradiation with 360 nm) retracted this μOR activation.^[4b]^

## Results and Discussion

Due to the clinical significance of fentanyl, the work described herein aims to expand the repertoire of photochromic fentanyl ligands. Creating such a repertoire would provide access to a “toolbox” of photochromic fentanyl ligands, with various photophysical properties and biological potencies that cater to a broader range of assays. In order to achieve this, the azobenzene moiety was replaced with an arylazopyrazole, as the latter has been shown to provide superior photophysical properties (Figure 1).^[13]^ A shift of the n–π* transition when compared to azobenzenes, provides a red‐shifted absorbance band for the *cis* isomer of arylazopyrazole. This is ideal for biological studies as it circumvents the sole use of UV irradiation for isomerization.^[14]^ This shift and thus, separation between the absorbance bands of *trans* and *cis* isomers, also provides near quantitative switching. In addition, the substitution pattern of these arylazopyrazole‐based photoswitches can be modified to tune the thermal stabilities of the *cis* isomer to range from seconds to weeks.^[15]^

**Figure 2:**
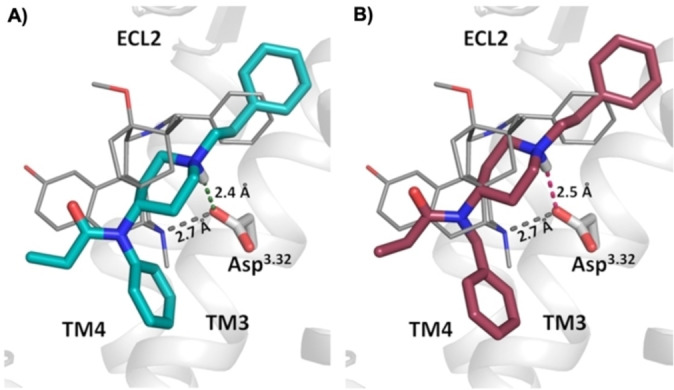
Modelled binding poses of A) fentanyl and B) its higher homologue fentanyl‐CH_2_ using the active‐state BU72‐bound μOR‐Nb39 X‐ray structure (PDB ID: 5 C1 M).^[2]^ For each ligand, the best‐scored poses are shown in comparison with the binding mode of BU72 in its μOR co‐crystal structure (grey sticks). As observed for BU72, both ligands form the canonical salt bridge of the protonated tertiary amine with Asp147^3.32^.

Pyrazole systems exist in nature, therefore, adapting the benzene moiety from the literature reported **PF2** structure into a pyrazole that contains 2 nitrogen atoms might result in further binding interactions in the μOR active site.^[16]^ For this work, the “4‐pyrazoles” system was chosen due to the reported long thermal half‐life of the *cis* isomer.^[15]^ The arylazopyrazole was attached to either the benzeneacetamide unit (compound **1**) or the phenylpropanamide unit (compound **2**) of fentanyl, as shown in Figure 1. When the azopyrazole was directly attached to the phenylpropanamide unit, the resulting photophysical properties included a fast thermal back‐isomerization of the *cis* isomer within seconds (described in more detail below). This might be due to the presence of a strong push–pull system, as previously described.[^13^, ^17^] To obtain more thermally stable photochromic ligands, the compound **3** series were designed to contain an extra methylene group to insulate phenyl from the anilino nitrogen, as shown in Figure 1. A fentanyl analogue that contains such a methylene insertion (referred to as fentanyl‐CH_2_) has been previously reported to maintain high binding affinity to the μOR, as well as full agonist activity.^[18]^ Furthermore, in this work, molecular modelling studies were performed with fentanyl‐CH_2_ (Figure 2). The results indicated that a key interaction is maintained between a conserved aspartic acid residue (Asp147^3.32^) on transmembrane helix 3 of μOR and a charged amine on the N‐piperidinyl unit of fentanyl‐CH_2_.^[19]^ This interaction has been reported to be crucial for receptor function.^[19]^ Interestingly, a novel μOR−Gα_i1_ cryoEM structure was reported very recently in complex with the fentanyl derivative lofentanil while this study was underway.^[20]^ Of particular note, the calculated binding poses of fentanyl‐CH_2_ are in accordance with the reported binding mode of lofentanil at the μOR.

Compound **3 a**, which contains a methyl‐capped pyrazole unit, was selected due to the reported long thermal stabilities of the *cis* isomer.^[15]^ The addition of a hydroxy group (**3 b**) and an azide moiety (**3 c**) was chosen to allow for differing binding interactions within the active site of μOR. Compound **4** was designed to include a triazene unit within the structure of fentanyl. While π‐conjugated triazenes have been reported to isomerize upon exposure to irradiation, linear triazenes have been found to undergo photofragmentation.^[21]^ Even though the latter class would be unable to reversibly isomerize, it was still of interest to incorporate such a structure into the pharmacophore due to synthetic accessibility. This class was designed to be incorporated within the core structure of fentanyl (compound **4**), and if photodegradation would indeed occur then that could involve the removal of the phenethyl moiety.[^21c^, ^21d^, ^21e^] This phenethyl moiety has been reported to be important for binding to the μOR, and therefore, removal of this moiety may result in diminished potency.[^19^, ^22^] Such probes that are either irreversibly activated or deactivated using light have been explored in the field of photopharmacology.^[23]^

**Figure 3:**
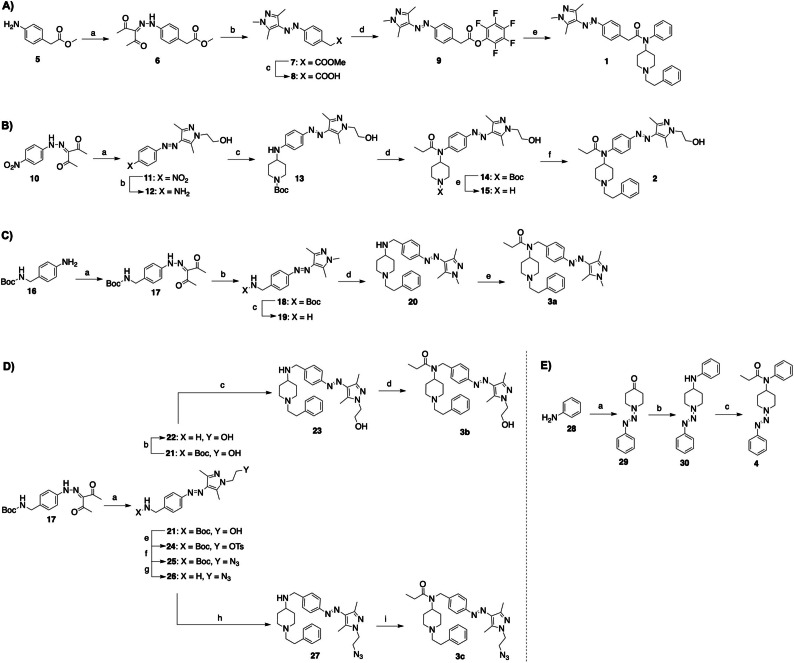
Synthesis of target compounds **1**, **2**, **3 a**, **3 b**, **3 c** and **4**. A) Synthesis of compound **1**. a) Methyl(4‐aminophenyl)acetate, NaNO_2_, HCl, H_2_O, AcOH, 0 °C, 45 min, then NaOAc, EtOH, 0 °C, 1 h, 27 %; b) methylhydrazine, EtOH, reflux, 3 h, 93 %; c) LiOH, THF/H_2_O (3 : 1), 20 °C, 4 h, 85 %; d) pentafluorophenol, EDCI, DMAP, THF, RT, 18 h, 34 %; e) *N*‐[1‐(2‐phenylethyl)‐4‐piperidinyl]aniline, DMF_dry_, N_2_, RT, 16 h, 8 %. B) Synthesis of compound **2**. a) 3‐(2‐(4‐Nitrophenyl)hydrazono)pentan‐2,4‐dione, 2‐hydrazinoethanol, EtOH, reflux, 3 h, 98 %; b) Na_2_S, THF/H_2_O, 80 °C, 3 h, 67 %; c) 1‐Boc‐4‐piperidone, NaHB(OAc)_3_, AcOH, DCE, RT, 24 h, 77 %; d) propionic anhydride, DMAP, toluene_dry_, N_2_, RT, 24 h, 10 %; e) TFA, CH_2_Cl_2_, 0 °C→RT, 1 h, 77 %; f) phenylacetaldehyde, NaHB(OAc)_3_, AcOH, DCE, RT, 24 h, 45 %. C) Synthesis of compound **3 a**. a) 4‐[(*N*‐Boc)aminomethyl]aniline, NaNO_2_, HCl, H_2_O, AcOH, 0 °C, 45 min, then NaOAc, EtOH, 0 °C, 1 h, 74 %; b) methylhydrazine, EtOH, reflux, 3 h, 97 %; c) TFA, CH_2_Cl_2_, 0 °C→RT, 1 h, 99 %; d) 1‐phenethyl‐4‐piperidone, NaHB(OAc)_3_, AcOH, DCE, RT, 20 h, 34 %; e) propionyl chloride, Et_3_N, CH_2_Cl_2_, 1 h, RT, 12 %. D) Synthesis of compounds **3 b** and **3 c**. a) Intermediate **17**, 2‐hydrazinoethanol, EtOH, reflux, 3 h, 99 %; b) TFA, CH_2_Cl_2_, 1 h, 0 °C→RT, 95 %; c) 1‐phenethyl‐4‐piperidone, NaHB(OAc)_3_, AcOH, DCE, 20 h, RT, 37 %; d) propionyl chloride, Et_3_N, CH_2_Cl_2_, 10 min, RT, 7 %; e) TsCl, Et_3_N, CH_2_Cl_2_, 16 h, RT, 76 %; f) NaN_3_, NaI, DMSO_dry_, N_2_, 65 °C, 24 h, 72 %; g) TFA, CH_2_Cl_2_, 0 °C→RT, 1 h, 99 %; h) 1‐phenethyl‐4‐piperidone, NaHB(OAc)_3_, AcOH, DCE, 20 h, RT, 64 %; i) propionyl chloride, Et_3_N, CH_2_Cl_2_, RT, 1 h, 71 %. E) Synthesis of compound **4**. a) Aniline, NaNO_2_, HCl, ACN/H_2_O (2 : 1), 0 °C, 45 min, then 4‐piperidone, K_2_CO_3_, RT, 1 h, 52 %; b) aniline, NaHB(OAc)_3_, AcOH, DCE, RT, 20 h, 31 %; c) propionyl chloride, Et_3_N, CH_2_Cl_2_, RT, 1 h, 51 %.

To obtain target compound **1**, commercially available starting material **5** was utilized in a diazotisation reaction to obtain diketone **6** (Figure 3A). This was followed by a condensation reaction that successfully resulted in azopyrazole **7** with 93 % yield. Following treatment with lithium hydroxide to form **8**, the free acid was reacted with pentafluorophenol to form intermediate **9** in 34 % yield. A coupling reaction with the previously synthesized *N*‐[1‐(2‐phenylethyl)‐4‐piperidinyl]aniline resulted in target compound **1** in 8 % yield.^[24]^ The synthesis of target compound **2** resembled a synthesis strategy used in the publication of Trauner et al. (Figure 3B).^[4b]^ A condensation reaction with previously reported diketone **10** allowed azopyrazole **11** to be obtained in 98 % yield.^[25]^ The nitro group of intermediate **11** was then reduced to amine **12** in 67 % yield. The pharmacophore moiety was then attached by reductive amination to obtain compound **13** in 77 % yield. Acylation of secondary amine **13** yielded intermediate **14** in poor yield due to an unwanted diacylated product. Following Boc deprotection under acidic conditions to obtain intermediate **15**, reductive amination was performed with phenylacetaldehyde to obtain target compound **2** in 45 % yield. The synthesis of target compound **3 a** began with the commercially available 4‐[(*N*‐Boc)aminomethyl]aniline **16** that was employed in a diazotisation reaction to obtain intermediate **17** in 74 % yield (Figure 3C). Intermediate **17** was used in a condensation reaction with commercially available methylhydrazine to obtain the azopyrazole‐containing intermediate **18**. Boc‐deprotection using trifluoroacetic acid then afforded amine **19** in quantitative yield. Reductive amination with 1‐phenethyl‐4‐piperidone resulted in intermediate **20**, which was then acylated to obtain target compound **3 a** in 12 % yield. The synthesis of target compounds **3 b** and **3 c** involved a condensation reaction using intermediate **17** and 2‐hydrazinoethanol that resulted in the azobenzene‐containing compound **21** (Figure 3D). For **3 b**, the Boc‐containing intermediate **21** was deprotected under acidic conditions, obtaining **22** in quantitative yield. Reductive amination to form **23**, followed by N‐acylation, yielded target compound **3 b** in 7 % yield. The synthesis of **3 c** was inspired by a previously reported literature procedure,^[26]^ where intermediate **21** was activated using *p*‐toluenesulfonyl chloride to form **24** in 76 % yield. Azide **25** was then obtained in 72 % yield upon reaction with sodium azide. Boc‐deprotection using trifluoroacetic acid then afforded amine **26** in quantitative yield. Reductive amination with commercially available 1‐phenethyl‐4‐piperidone resulted in compound **27**, which was then acylated to obtain target compound **3 c** in 71 % yield. Synthesis of target compound **4** began with a diazotisation reaction of aniline **28** with commercially available 4‐piperidone to obtain triazene **29** in 52 % yield (Figure 3E). Triazene **29** has been previously obtained by an alternative synthetic route.^[27]^ Reductive amination with aniline resulted in precursor **30** in 31 % yield. An acylation reaction allowed access to target compound **4** in 51 % yield.

**Figure 4:**
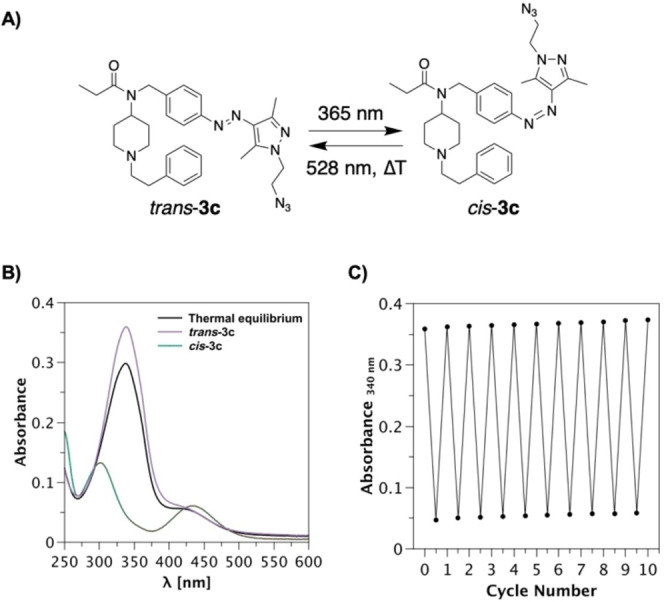
Light‐induced isomerization and cycle performance of compound **3 c**. This compound is shown here as an example, as compounds **1**, **3 a**, **3 b** and **3 c** displayed similar photophysical properties (see the Supporting Information). A) The structural changes that ensue upon photo‐induced isomerization of **3 c**. B) UV/Vis absorption spectra of thermal equilibrium, *trans* and *cis* isomers. The *cis* isomer was accessed by irradiation with 365 nm, whereas the *trans* isomer was obtained with 528 nm irradiation. C) Cycle performance of **3 c** upon alternating irradiation of 365 and 528 nm. Data points were recorded at the absorbance maximum of the respective *trans* isomer (340 nm). Results are shown of **3 c** (20 μM) in buffer solution (Tris ⋅ HCl, pH 7.5)+0.2 % DMSO at 25 °C.

The photophysical properties of compounds **1**, **2**, **3 a**, **3 b**, **3 c** and **4** were evaluated. This involved obtaining UV/Vis absorbance spectra of thermal equilibrium, *trans* and *cis* isomers, as well as evaluating thermal stability of the *cis* isomer, cycle performance and photostationary states (Table 1, Figure 4 and the Supporting Information).

**Table 1:** Summary of experimental photophysical properties.^[a]^

Cmpd	Solvent	PSS^[b]^		*t*_1/2_^[d]^
		*cis*→*trans trans* : *cis*	*trans*→*cis trans* : *cis*	*cis* isomer
1	DMSO	94 : 6	6 : 94	6.2
1	buffer^[c]^	99 : 1	7 : 93	6.4
2	DMSO	99 : 1	20 : 80 ^[e]^	26^[f]^
3a	DMSO	95 : 5	7 : 93	5.3
3a	buffer^[c]^	93 : 7	6 : 94	6.0
3b	DMSO	96 : 4	7 : 93	6.4
3b	buffer^[c]^	97 : 3	7 : 93	10
3c	DMSO	94 : 6	4 : 96	8.7
3c	buffer^[c]^	93 : 7	9 : 91	11

[a] Isomerization was obtained by irradiation of 365 nm (*cis* isomer) and 528 nm (*trans* isomer) at 25 °C, except for compound **2** that required 400 nm to obtain the *cis* isomer. [b] PSS was determined by HPLC measurements. [c] Buffer solution (Tris ⋅ HCl Buffer, pH 7.5)+0.2 % or 0.5 % DMSO, see the Supporting Information. [d] Experiment was performed at 27 °C. [e] Estimated PSS by UV/VIS measurements with irradiation wavelength of 400 nm to obtain *cis* isomer. [f] Value reported in seconds.

Compounds **1**, **3 a**, **3 b** and **3 c** displayed similar and promising photophysical properties. When compared to the previously reported azobenzene analogue (**PF2**),^[4b]^ the respective *cis* isomers of compounds **1** and series **3** displayed an increase of the n→π* transition, allowing for a red‐shifted wavelength of 528 nm to be used for isomerization back to the *trans* isomer.

In addition, these compounds exhibited long thermal half‐lives of their respective *cis* isomer, ranging from 5 to 11 days in both DMSO and buffer solutions (Table 1). Compounds **1**, **3 a**, **3 b** and **3 c** also exhibited resistance to cycle fatigue, as toggling between *trans* and *cis* isomers was achieved for at least 10 cycles (Figure 4 and the Supporting Information). Exciting the π→π* transition of *trans*‐**2** to obtain the *cis* isomer, required a wavelength of 400 nm to be used. Such a red‐shifted photoswitch is ideal for biological purposes and was the result of a slight bathochromic shift in absorbance. However, the resulting *cis* isomer of compound **2** was found to be significantly less thermally stable (*t*_1/2_=26 s, Table 1). Even though a thermally unstable ligand was outside the scope of the biological investigations described herein, such a ligand may be valuable when investigating receptor function, especially the role of dynamics. Interestingly, while compounds **1**, **2** and **3** possess an arylazopyrazole that undergoes isomerization upon exposure to light, compound **4** that possesses a triazene was found to decompose upon exposure to UV irradiation. This finding was consistent with literature and was confirmed by UV/Vis spectroscopy and HPLC measurements (see the Supporting Information).[^21b^, ^21c^, ^21d^]

Lead photoswitchable compounds **1**, **3 a**, **3 b** and **3 c**, as well as previously reported **PF2**, were subjected to radioligand binding studies to determine compound affinity for the μOR. To obtain each of the respective isomers, each compound in solution was irradiated with their corresponding wavelength prior to biological analysis. Each isomer was then subjected to evaluation, with results shown in Table 2. Control compounds in these investigations included fentanyl and the previously reported fentanyl‐CH_2_.^[18b]^

**Table 2:** Radioligand binding studies.^[a]^

Cmpd	μOR_wt_		
	*K*_i_ [nM±SEM]^[b]^	*K*_i_ ratio^[c]^	(*n*)^[d]^
fentanyl	10±0.15		10
fentanyl‐CH_2_	100±28		5
*trans*‐**PF2** ^[e]^	16±0.58	2.8 (*trans*)	3
*cis*‐**PF2**	44±9.8		5
*trans*‐**1**	1 400±260	2.0 (*cis)*	4
*cis*‐**1**	690±93		5
*trans*‐**3 a**	1 400±260	1.4 (*cis)*	4
*cis*‐**3 a**	980±170		4
*trans*‐**3 b**	3 000±170	1.0	4
*cis*‐**3 b**	3 000±700		3
*trans*‐**3 c**	880±210	1.9 (*trans*)	4
*cis*‐**3 c**	1 700±500		5
**4**	960±88		6

[a] Binding data to wild‐type μOR (μOR_wt_) determined by competition binding with [^3^H]diprenorphine; samples were pre‐irradiated prior to the assay, with 365 nm for isomerization to obtain the *cis* isomer and 528 nm for isomerization to obtain the *trans* isomer. [b] Mean *K*_i_ value [nM±SEM]. [c] The isomer shown in brackets has a lower *K*_i_ value than its respective isomer. [d] Number of individual experiments each performed in triplicate. [e] Irradiation of 420 nm required for isomerization to the *trans* isomer.

In these studies, fentanyl displayed a binding affinity of 10 nM to the μOR. This low nanomolar binding affinity was maintained after substitution at the *para*‐position of the aniline to obtain the **PF2** photoswitchable system, as *trans*‐**PF2** and *cis*‐**PF2** were found to have *K*_i_ values of 16 and 44 nM, respectively. Although the insertion of a methylene unit in fentanyl‐CH_2_ attenuated binding to a *K*_i_ value of 100 nM, this range of affinity was considered as a good starting point for the development of azopyrazole photoswitchable analogues. Here, it could be observed that the introduction of an azopyrazole photoswitch moiety in compounds **1**, **3 a**, **3 b** and **3 c** led to binding affinities that range from 690 nM to 3 μM. Compared to their respective *trans* isomers, both methylpyrazoles *cis*‐**1** and *cis*‐**3 a** showed approximately a twofold higher binding affinity, with obtained values of 690 and 980 nM, respectively. When extending the substituent in position 1 of the pyrazole with a hydroxyethyl group, affinity slightly decreased to 3 μM for both the isomers *trans*‐**3 b** and *cis*‐**3 b**. In contrast, replacing the methyl group of **1** by azidoethyl in **3 c**, resulted in *K*_i_ values of 880 nM and 1.7 μM for *trans*‐**3 c** and *cis*‐**3 c**, respectively. Here, *trans*‐**3 c** binds to μOR with two‐fold higher affinity than *cis*‐**3 c**.

**Figure 5:**
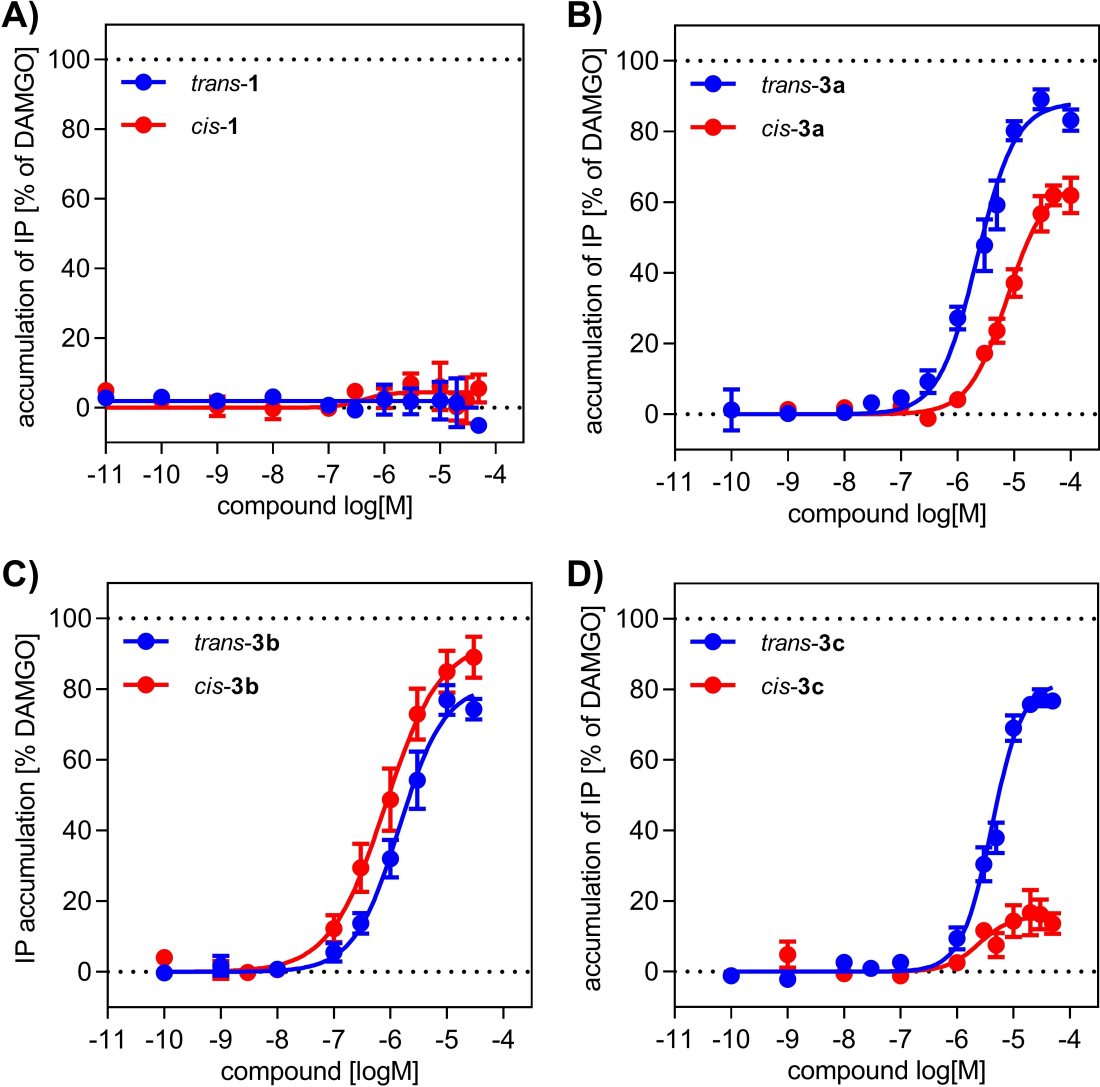
Activation of the μOR by selected photoswitches. G protein‐mediated receptor activation by A) *trans*‐**1**, *cis*‐**1**, B) *trans*‐**3 a**, *cis*‐**3 a**, C) *trans*‐**3 b**, *cis*‐**3 b**, and D) *trans*‐**3 c**, *cis*‐**3 c** was measured by applying the IP‐One® accumulation assay in HEK293T cells transiently co‐transfected with μOR and the hybrid G protein Gα_qi5HA_. While *trans*‐**1** and *cis*‐**1** behave as antagonists, *trans*‐**3 a,b,c** and *cis*‐**3 a,b** show strong partial agonist activity and *cis*‐**3 c** reveals only weak partial agonist activity. The great difference in activation between *trans* and *cis* isomers of **3 c** indicates *trans‐*/*cis*‐**3 c** to be a promising tool for photoswitching experiments. Graphs show mean curves (±SEM) of 4–11 single experiments each performed in duplicate.

As the binding profiles for compounds **1**, **3 a**, **3 b** and **3 c** displayed moderate differences between *trans* and *cis* isomers, it became of interest to determine whether these photoswitches and their respective isomers show a larger difference in activating the μOR. Therefore, ligand‐mediated μOR activation was evaluated in a G protein activation assay (IP‐One^®^), measuring the agonist‐stimulated accumulation of IP in HEK293T cells that were transiently co‐transfected with the receptor and the hybrid G protein Gα_qi5HA_.^[28]^ Each photoswitch‐containing compound was irradiated prior to biological analysis with the appropriate wavelength to obtain either their respective *trans* or *cis* isomer, which were then used to determine dose‐response curves in comparison to the full agonist reference DAMGO (Table 3, Figure 5 and the Supporting Information). Fentanyl and the homologous fentanyl‐CH_2_ both behaved as full agonists with potencies of 2.6 and 77 nM, respectively. These results demonstrated that the insertion of the methylene group into fentanyl is well tolerated for receptor activation, though at reduced potency. Surprisingly, we could not determine any significant difference in activation properties between *trans*‐ and *cis‐* **PF2** (*trans‐* **PF2**: EC_50_=96 nM, *E*_max_=93 %; *cis‐* **PF2**: EC_50_=85 nM, *E*_max_=95 %; see the Supporting Information). These results are different to previously published data that describes agonist activity for *trans‐* **PF2** and after irradiation, an inactive effect for *cis‐* **PF2**.^[4b]^ This can be explained by the application of different biological assays. In contrast to monitoring the ionotropic response,^[4b]^ this work focused on examining the metabotropic response. As observed for the binding affinity of the new photoswitch ligands, the addition of an azopyrazole to the aniline moiety of fentanyl‐CH_2_ resulted in an attenuation of activation potency for *trans‐/cis‐* **3 a**,**b**,**c** with EC_50_ values that range from 1600 nM (for *cis‐* **3 b**) to 7900 nM (for *cis*‐**3 a**). These compounds displayed agonist properties with intrinsic activities, described by *E*_max_ values, that range from 18 % (for *cis‐* **3 c**) to 100 % (for *cis‐* **3 b**). Interestingly, the addition of the azopyrazole photoswitch unit to the acylamide of fentanyl‐CH_2_ in *trans‐/cis‐* **1** resulted in a complete loss of intrinsic activity (Table 3, Figure 5A). Within the series of azopyrazole‐substituted anilines, the *N*‐methyl derivative **3 a** and the hydroxyethyl derivative **3 b** displayed little to moderate differences in the activation profile between their respective *trans* and *cis* isomers.

**Table 3:** Ligand‐mediated activation of the μOR.^[a]^

Cmpd	μOR_wt_				
	EC_50_ [nM±SEM]^[b]^	EC_50_ ratio^[c]^	*E*_max_ [%±SEM]^[d]^	Δ*E*_max_ [%]^[e]^	(*n*)^[f]^
DAMGO	5.0±0.62		100		7
fentanyl	2.6±0.27		99±3		6
fentanyl‐CH_2_	77±19		102±2		6
*trans*‐**PF2**	96±16	1.1 (*cis*)	93±5	2 (*cis*)	4
*cis*‐**PF2**	85±43		95±8		3
*trans*‐**1**	n/a		<5		4
*cis*‐**1**	n/a		<5		4
*trans*‐**3 a**	2,400±350	3.3 (*trans*)	93±2	24 (*trans*)	10
*cis*‐**3 a**	7,900±690		69±5		8
*trans* **‐3 b**	2,000±440	1.3 (*cis*)	88±5	12 (*cis*)	8
*cis* **‐3 b**	1,600±510		100±3		9
*trans*‐**3 c**	4,700±510	2.0 (*cis*)	90±2	72 (*trans*)	11
*cis*‐**3 c**	2,300±550		18±5		7
**4**	2,400±400		106±2		8

[a] IP‐One accumulation assay (Cisbio) with HEK 239T cells transiently co‐transfected with the cDNAs of the human μOR and the hybrid G protein Gα_qi5HA_. [b] Mean EC_50_ values are given in [nM±SEM]. [c] The isomer shown in brackets has a lower EC_50_ value than its respective isomer. [d] Maximum receptor activation in [%±SEM] relative to the full effect of DAMGO. [e] Δ*E*_max_ refers to the difference between *E*_max_ values. The isomer shown in brackets has a higher *E*_max_ value than its respective isomer. [f] Number of individual experiments each performed in duplicates. n/a: not applicable due to poor receptor activity.

Compound *trans*‐**3 b** displayed a potency of 2000 nM and an efficacy of 88 %, while its respective isomer *cis*‐**3 b** activated μOR with an EC_50_ of 1600 nM and an *E*_max_ of 100 %. A better difference in activity could be observed for *trans‐* **3 a** when compared to *cis‐* **3 a**, with a threefold higher activity found for the former isomer (EC_50_=2400 nM, *E*_max_=93 %) than that found for the latter (EC_50_=7900 nM, *E*_max_=69 %). Most interestingly, the azidoethyl derivative **3 c** revealed substantial differences in the activation profile of both isomers. While *trans‐* **3 c** acts as a strong partial agonist with an efficacy of 90 % (EC_50_=4700 nM), its isomer *cis‐* **3 c** only displayed weak partial agonist properties with an *E*_max_ value of 18 % (EC_50_=2300 nM). This clear difference in efficacy offers the opportunity to use *trans‐/cis‐* **3 c** as a photoswitch tool that allows targeted *on‐* and *off‐*switching of μOR activity by irradiation. Although compound **4** decomposes upon irradiation, we were interested in its biological properties at the μOR. Binding affinity was determined with a *K*_i_ value of 960 nM and the potency to activate μOR was measured showing an EC_50_ of 2400 nM (Tables 2 and 3). These values are about 100‐ and 1000‐fold worse than for fentanyl, which may be explained by an attenuated basicity induced by the inductive effect of the azo group.^[29]^

**Figure 6:**
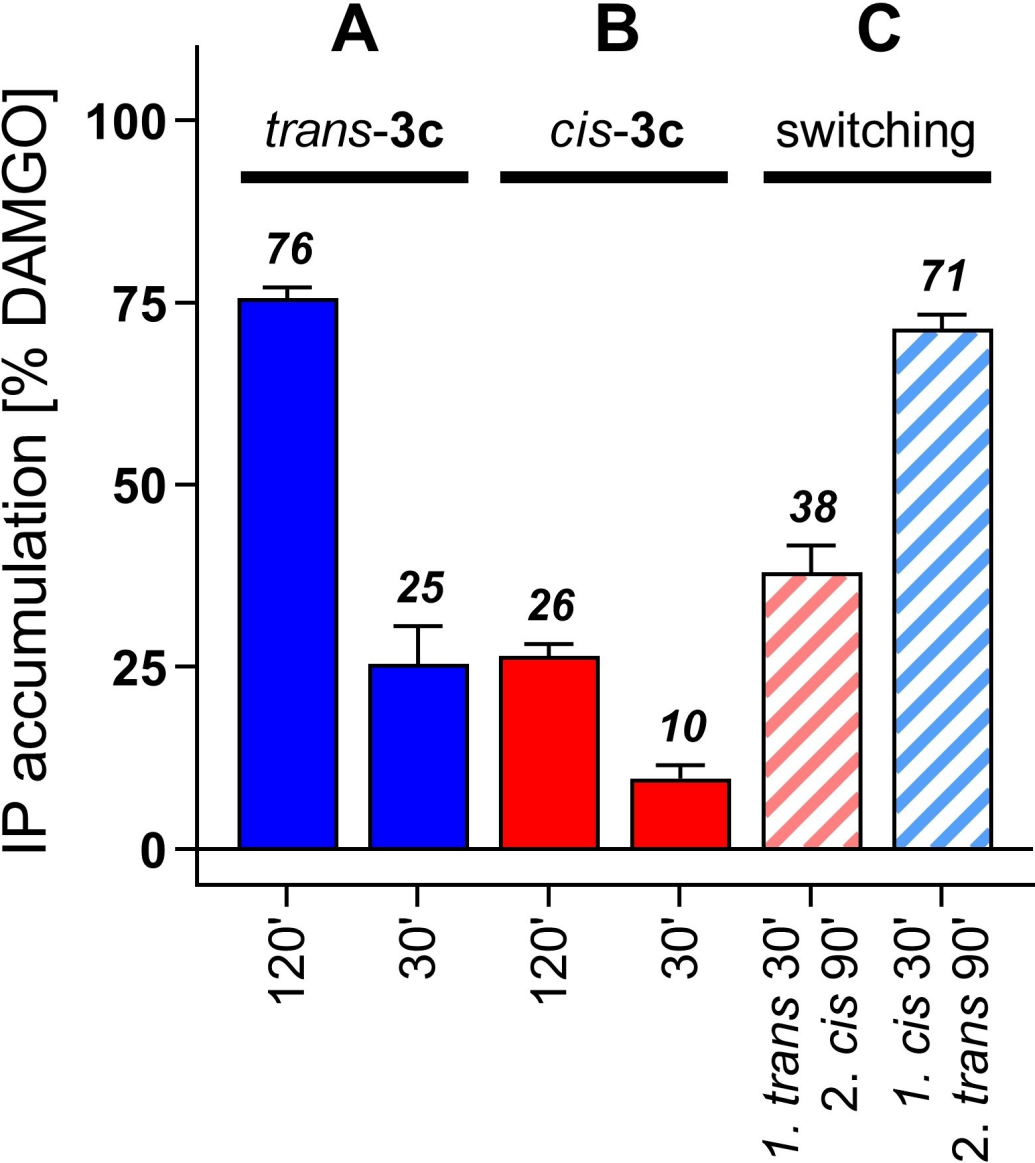
Photoisomerization of *trans‐*/*cis*‐**3 c** during incubation with μOR‐expressing HEK293T cells determined in an IP accumulation assay. Receptor efficacy induced by 30 μM of A) *trans*‐**3 c** and B) *cis*‐**3 c** after 30 or 120 min. C) In‐situ photo‐induced switching of *trans*‐**3 c** to *cis*‐**3 c** (red stripes) or for *cis*‐**3 c** to *trans*‐**3 c** (blue stripes) after 30 min reveals full conversion of *trans*/*cis* and *cis*/*trans* isomers, resulting in different μOR activation. Incubation was initiated by irradiation with 528 nm for 180 s for *trans*‐**3 c** (A, C) or with 365 nm for 20 s to obtain *cis*‐**3 c** (B, C). For switching during incubation, a second irradiation step was performed after 30 min with 365 nm for 20 s (red stripes) and 528 nm for 180 s (blue stripes) (C). Bars represent efficacy relative to the effect of DAMGO (after 120 min) as mean values (%±SEM) of 6–11 single experiments each performed in quadruplicate.

With the identification of *trans‐/cis*‐**3 c** as a photoswitch compound that displays different activation properties for its *trans* and *cis* isomers, it became of interest to determine whether this tool could also be switched during a cell incubation experiment and thereby, enable the activation and inactivation of μOR in situ. To prove this, cells were incubated with 30 μM of *trans‐*/*cis‐* **3 c** in a micro‐plate format. Incubation of the cells was initiated by irradiation of the wells with a wavelength of 528 nm (for 180 s) to adjust to *trans* isomer and at 365 nm (for 20 s) to adjust to *cis* isomer. In situ photoisomerization was performed after 30 min and second messenger accumulation was continued for a total incubation time of 120 min. To be able to compare the amount of IP accumulation at the time of in‐situ switching to that after in‐situ switching, receptor activation was additionally determined after 30 min. After 120 min, *trans*‐**3 c** and *cis*‐**3 c** activated μOR with an efficacy of 76 and 26 %, respectively (Figure 6A, B), which reflected the strong and weak partial agonist properties that were determined for both isomers in dose‐response experiments (Table 3, Figure 5). A similar ratio of efficacy between these two isomers (25 % for *trans*‐**3 c**, 10 % for *cis*‐**3 c**) was found after 30 min. Switching *trans*‐**3 c** to *cis*‐**3 c** resulted in receptor activation of 38 % (Figure 6C, red stripes). Given that *trans*‐**3 c** induces approximately 25 % of second messenger (within 30 min), the additionally formed IP (13 % over 90 min) indicated a complete transformation of the strong partial agonist *trans*‐**3 c** to the weakly activating *cis*‐**3 c**. For the other way around, the shorter 30 min incubation with *cis*‐**3 c** and subsequent switching to *trans*‐**3 c** resulted in an efficacy of 71 %. Here, the additionally accumulated IP (61 % during 90 min) can clearly be explained by the fact that conversion of *cis*‐**3 c** to *trans*‐**3 c** was successfully achieved (Figure 6C, blue stripes). With compound *trans‐*/*cis*‐**3 c**, a photoswitch tool has been developed that displays very promising activation properties, which enable the control of activation states of the μ‐opioid receptor in vitro. When switched to the *trans* isomer, compound *trans*‐**3 c** elicits a near full agonist receptor response, and when switched to the *cis* isomer, receptor activation is diminished. This is a significant finding since developing a “switch on” and “switch off” tool has been an obstacle in the field of photopharmacology so far, especially when applied to commercially accessible and cell‐based assays.^[30]^ Switching receptor efficacy by non‐invasive means, such as irradiation by light, reveals azopyrazole **3 c** to be a useful tool for future mechanistic investigations of the μOR.

## Conclusion

The work herein describes the generation of diverse photoresponsive fentanyl‐based ligands, with different photophysical and biochemical properties. In this way, a “toolbox” was developed that could be applied in a diverse range of biochemical investigations to better understand the μOR. By attaching an azopyrazole photoswitch to the benzeneacetamide moiety (compound **1**), receptor activation was abolished. This might indicate that large modifications at this position of fentanyl are less tolerated. Although most of the compounds in the series displayed relatively long thermal stabilities of the *cis* isomer, compound **2** isomerized back to the *trans* isomer with a thermal half‐life of 26 s. This might be beneficial for dynamic investigations that require a fast‐switching ligand. However, continuous near‐UV irradiation is required (400 nm), which might not be as compatible with cell‐based assays. Each of the compounds in series **3** exhibited excellent photophysical properties, with good PSS values and the ability to switch between isomers for at least 10 cycles. The lead ligand in this series was azide **3 c**, which displayed a significant difference between the *trans* and *cis* isomers when comparing the maximum response of receptor activation. The *trans* isomer of compound **3 c** was able to activate the μOR to a 90 % response, whereas the *cis* isomer significantly reduced the maximum activation response to 18 %. This suggests compound **3 c** to be a valuable tool that potently targets μOR and displays “switch on” and “switch off” capabilities that can be accessed non‐invasively by using light. Importantly, photo‐induced switching, and the resulting biological effects of *trans*‐/*cis*‐**3 c** that ensue, was successfully achieved in situ. Furthermore, the azide moiety allows this ligand to be accessible to further bioorthogonal reactions, such as the known click reaction, to covalently attach the ligand to the μOR.^[31]^ Compound **4** proved not to be able to reversibly switch between isomers but decomposed upon exposure to UV irradiation. This could have provided an opportunity to irreversibly deactivate ligand activity in situ by using light; however, the incorporation of the triazene unit into the fentanyl structure diminished ligand potency towards μOR.

The combination of results described herein establishes the diverse range of photoswitchable fentanyl ligands that have been developed, which provide access to beneficial tool compounds that could aid in better understanding the dynamic and/or kinetic mechanisms surrounding μOR and interacting ligands.

## Experimental Section

For details of molecular modelling, synthetic procedures, chemical and photochemical characterizations, as well as biochemical evaluations, see the Supporting Information.

## Conflict of interest

The authors declare no conflict of interest.

1

## Supporting information

As a service to our authors and readers, this journal provides supporting information supplied by the authors. Such materials are peer reviewed and may be re‐organized for online delivery, but are not copy‐edited or typeset. Technical support issues arising from supporting information (other than missing files) should be addressed to the authors.

Supporting Information

## Data Availability

The data that support the findings of this study are available from the corresponding author upon reasonable request.
