# Supplementary material for: Photochromic Fentanyl Derivatives for Controlled μ‐Opioid Receptor Activation
Source: Chemistry. 2022 Sep 12;28(63):e202201515. doi: 10.1002/chem.202201515 (PMC9826449; doi:10.1002/chem.202201515)
Supplement: Supplementary file 1 — Supporting Information [file CHEM-28-0-s001.pdf]

Chemistry–A European Journal

Supporting Information

Photochromic Fentanyl Derivatives for Controlled μ-Opioid Receptor Activation

Ranit Lahmy, Harald Hübner, Maximilian F. Schmidt, Daniel Lachmann, Peter Gmeiner,* and Burkhard König*

Table of Contents

1. Supplementary Molecular Modeling Information 2
 1.1. General Information 2
2. Supplementary Chemistry Information 3
 2.1. General Information 3
 2.2. Chemistry Synthesis Procedures 4
 2.3. Analytical HPLC Chromatograms for Purity Determination in DMSO 12
3. Supplementary Photophysical Information 18
 3.1. General Information 18
 3.2. UV/Vis Absorption Spectroscopy, Cycle Performance and Thermal Stabilities 19
 3.3. Analytical HPLC Chromatogram for PSS Determination 24
 3.4. Degradation of Compound 4 Upon Exposure to UV Irradiation 40
  3.4.1. UV/Vis Spectroscopy to Monitor Effects of Light Exposure. 40
  3.4.2. Analytical HPLC to Monitor Effects of Light Exposure 41
4. Supplementary Biochemical Information 43
 4.1. General Information 43
 4.2. Supplementary Figures and Tables 44
5. ^{1}H and ^{13}C NMR Spectra 46
6. References 75

1. Supplementary Molecular Modeling Information

1.1. General Information

Molecular docking was conducted using GLIDE software (Schrödinger LLC). Geometries of fentanyl and fentanyl-CH_2 were prepared and energetically optimized using the LigPrep module (Schrödinger LLC). The tertiary amines were protonated, resulting in a formal charge of +1 for each ligand. The recently disclosed active-state µOR-Nb39 X-ray structure bound to the agonist BU72 (PDB-ID 5C1M)[1] was used as receptor and was prepared by means of the Protein Preparation Wizard module (Schrödinger LLC). Water molecules within the orthosteric binding site were kept. Amino acid side chains containing hydrogen-bond donors and acceptors were optimized for hydrogen bonding and were modelled in their dominant protonation state at pH 7. The residue Asp147$^{3.32}$ was chosen as box center for the grid box. Molecular Docking was performed using the default settings of the obtained docking poses were inspected manually and according to the GLIDE docking score. Visualization was performed using the PyMOL Molecular Graphics System, Version 2.1.1 (Schrödinger, LLC).

2. Supplementary Chemistry Information

2.1. General Information

Commercial reagents were obtained from Merck, Sigma-Aldrich, TCI Deutschland GmbH, ABCR GmbH or Fluorochem, and were used without further purification. Solvents were used in P.A. quality and if necessary, dried according to common procedures. Anhydrous reactions were performed using dried glassware under a nitrogen or argon atmosphere, unless otherwise specified. Technical grade solvents were used for column chromatography without further purification. Flash chromatography was performed using Biotage Isolera One System for normal phase chromatography, using Davisil Chromatographic Silica Media 60 Å (particle size 40-63 μM, Merck). For reversed phase chromatography, Biotage SNAP Cartridges KP-C18-HS were used. Analytical thin layer chromatography (TLC) was performed on silica gel 60 F-254 with a 0.2 mm layer and aluminium-backed plates (Merck). Visualization was obtained by fluorescence quenching under UV light (short and long wave) and/or by staining the plate with potassium permanganate stain (60 mM $KMnO_4$, 480 mM K_2CO_3 and 5% w/v NaOH) and vanillin-H_2SO_4 solution (0.5 g vanillin, 85 mL ethanol, 10 mL conc. acetic acid, 3 mL conc. H_2SO_4). Preparative high-performance liquid chromatography (HPLC) was performed using Agilent1100 Series with a Phenomenex Luna 10 μM C18 column (100 Å, 250 x 21.2 mm) and a solvent flow rate of 20 mL/min. Analytic HPLC measurements were performed using Agilent UHPLC-MSD-System (column: Phenomenex Luna C18(2), 100 Å, 150 x 2.00 mm) and Agilent 1220 Infinity LC System (column: Phenomenex Luna, 3 μM C18(2), 100 Å 150 x 2.00 mm). All biologically tested compounds possessed a purity of ≥ 95%, which was determined by analytical HPLC with wavelength detections of 220 nm and 254 nm. Infrared (IR) Spectroscopy was recorded using Agilent Technologies Cary 630 FTIR instrument. NMR spectra were recorded on a Bruker Avance III HD 600 (^{1}H 600.25 MHz, ^{13}C 150.95 MHz, T = 300K), with solvents specified. The chemical shifts were reported as δ values in parts per million (ppm), referenced to the appropriate and specified solvent peak. Resonance multiplicity is abbreviated as: 's' (singlet), 'd' (doublet), 't' (triplet), 'q' (quartet) and 'm' (multiplet). J-coupling constants (J) were recorded in Hz. Mass spectra were recorded using Finnigan MAT-SSQ 710 A, ThermoQuest Finnigan TSQ 7000, Agilent Q- TOF 6540 UHD, or a JeolAccuTOF GCX instrument.

2.2. Chemistry Synthesis Procedures

Compound **10**[2], **PF2**[3] and **fentanyl-CH$_2$**[4] were synthesized according to literature procedures.

General Procedure 1: Diazotisation
A mixture of the respective aniline derivative (1.0 eq.), acetic acid (2 mL/mmol) and conc. HCl (0.25 mL/mmol) was allowed to stir at 0 °C. A solution of $NaNO_2$ (1.2 eq.) dissolved in a minimum amount of water was added dropwise to the reaction mixture. After stirring for 45 min at 0 °C, the resulting diazonium salt mixture was added to a suspension of acetylacetone (1.3 eq.) and NaOAc (3.0 eq.) in EtOH (2 mL/mmol). The reaction mixture was then stirred at room temperature for 1 h. The resulting bright yellow-orange precipitate was collected, filtered, washed with ice-cold water and hexane (1:1), and dried *in vacuo*. The desired product was obtained without further purification.

General Procedure 2: Pyrazole formation
To a stirred solution of the respective diketone (1.0 eq.) in EtOH (10 mL/mmol), 2-hydrazinoethanol (1.1 eq.) was added. The reaction mixture was allowed to reflux for 3 h. After cooling to room temperature, the solvent was removed *in vacuo*, yielding the desired product. If necessary, further purification was employed, as specified.

General Procedure 3: Deprotection of the Boc-protecting group
To a stirred solution of the Boc-protected amine (1.0 eq.) and DCM (10 mL/mmol), TFA (1 mL/mmol) was added dropwise at 0 °C. After 1 h of stirring at room temperature, the reaction was quenched with 2M NaOH (10 mL/mmol), followed by an extraction with DCM. The combined organic layer was dried with Na_2SO_4 and filtered. The solvent was them removed *in vacuo* to afford the desired product. If necessary, further purification was employed, as specified.

Methyl 2-(4-(2-(2,4-dioxopentan-3-ylidene)hydrazineyl)phenyl)acetate (6)
General Procedure 1 was followed, using commercially available methyl(4-aminophenyl)acetate (479 mg, 2.90 mmol) to obtain **6** as a yellow solid (198 mg, 0.717 mmol, 27%). ^{1}H NMR (400 MHz, $CDCl_3$): δ (ppm) = 7.39-7.37 (m, 2H), 7.33-7.31 (m, 2H), 3.71 (s, 3H), 3.64 (s, 2H), 2.60 (s, 3H), 2.48 (s, 3H). ^{13}C NMR (101 MHz, CDCl3): δ =198.14, 197.24, 171.89, 140.81, 133.40, 131.82, 130.75, 116.60, 52.32, 40.73, 31.82, 26.78. ESI-MS (m/z): [*M*+H]$^+$ found: 277.1188.

Methyl (*E*)-2-(4-((1,3,5-trimethyl-1*H*-pyrazol-4-yl)diazenyl) phenyl)acetate (7)
General Procedure 2 was followed, using intermediate **6** (178 mg, 0.644 mmol) and methylhydrazine (0.03 mL, 0.644 mmol, 1.0 eq.). The crude product was subjected to silica chromatography (MPLC), eluting at 100% DCM to 10% MeOH/DCM to obtain **7** as a yellow oil (172 mg, 0.601 mmol, 93%). ^{1}H NMR (400 MHz, CDCl3): δ (ppm) = 7.73 (d, J = 8.35 Hz, 2H), 7.36 (d, J = 8.35 Hz, 2H), 3.77 (s, 3H), 3.70 (s, 3H), 3.68 (s, 2H), 2.56 (s, 3H), 2.49 (s, 3H). ^{13}C NMR (101 MHz, CDCl3): δ (ppm) = 171.90, 152.84, 142.55, 138.86, 135.25, 135.21, 129.96, 122.06, 52.24, 41.11, 36.09, 13.93, 10.07. ESI-MS (m/z): [*M*+H]$^+$ found: 287.1505.

(*E*)-2-(4-((1,3,5-trimethyl-1*H*-pyrazol-4-yl)diazenyl)phenyl)acetic acid (8)
A mixture of **7** (157 mg, 0.548 mmol) and LiOH·H_2O (0.03 mL, 0.644 mmol, 1.0 eq.) in THF (7.0 mL) and water (2.3 mL) was allowed to stir at room temperature. After 18 h, 10% citric acid solution (5 mL) was added, and the resulting solution was extracted with ethyl acetate (2 x 10 mL). The combined organic layer was then washed with 10% $NaHCO_3$ (1 x 10 mL), followed by a wash with sat. NaCl solution (1 x 10 mL). This solution was then dried with Na_2SO_4 and concentrated *in vacuo* to obtain **8** as an orange solid (127 mg, 0.466 mmol, 85%). ^{1}H NMR (400 MHz, MeOD): δ (ppm) = 7.72 (d, J = 8.40 Hz, 2H), 7.40 (d, J = 8.40 Hz, 2H), 3.78 (s, 3H), 3.67 (s, 2H), 2.59 (s, 3H), 2.45 (s, 3H). ^{13}C NMR (101 MHz, MeOD): δ = 175.22, 153.86, 143.05, 143.05, 140.99, 137.76, 135.89, 131.11, 122.78, 41.64, 36.05, 13.83, 9.75. ESI-MS (m/z): [*M*+H]$^+$ found: 273.1349.

Perfluorophenyl (*E*)-2-(4-((1,3,5-trimethyl-1*H*-pyrazol-4-yl)diazenyl)phenyl)acetate (9)
To a solution of intermediate **8** (116 mg, 0.43 mmol) in THF (5 mL), pentafluorophenol (78.4 mg, 0.43 mmol, 1 eq.), EDCI (66.1 mg, 0.84 mmol, 1.0 eq.) and DMAP (5.2 mg, 0.08 mmol, 0.1 eq.) was added. The reaction mixture was allowed to stir for 16 h at rt. The mixture was then filtered, and the resulting filtrate was then concentrated *in vacuo*. The crude product was subjected to silica chromatography (MPLC), eluting at 100% DCM to 10% MeOH/DCM to obtain **9** as an orange solid (63 mg, 0.144 mmol, 34%). ^{1}H NMR (400 MHz, $CDCl_3$): δ (ppm) = 7.79 (d, J = 8.36 Hz, 2H), 7.45 (d, J = 8.35 Hz, 2H), 4.02 (s, 2H), 3.79 (s, 3H), 2.58 (s, 3H), 2.50 (s, 3H). ^{13}C NMR (101 MHz, $CDCl_3$): δ = 167.40, 153.24, 142.67, 139.13, 135.27, 133.10, 129.99, 122.38, 40.11, 36.09, 13.92, 10.11. ESI-MS (m/z): $[M+H]^+$ found: 439.1195.

(*E*)-*N*-(1-phenethylpiperidin-4-yl)-*N*-phenyl-2-(4-((1,3,5-trimethyl-1*H*-pyrazol-4-yl)diazenyl) phenyl)acetamide (1)
A solution of pentafluorophenyl ester **9** (50 mg, 0.11 mmol), *N*-[1-(2-phenylethyl)-4-piperidinyl]aniline[5] (32 mg, 0.11 mmol, 1.0 eq.) and Et_3N (0.05 mL, 0.34 mmol, 3.0 eq.) in DMF (3 mL) was stirred at room temperature. After 16 h, the solvent was removed under reduced pressure and the crude product was subjected to silica chromatography (MPLC), eluting at 100% DCM to 10% MeOH/DCM. The product was further purified by preparative HPLC (solvent A: H_2O [0.05 Vol% TFA], solvent B: MeCN; gradient A/B: 0-20 min: 90/10 to 2/98, 20-25 min: 2/98), eluting at 40% MeCN, to obtain **1** as an orange solid (4.6 mg, 0.009 mmol, 8%). ^{1}H NMR (400 MHz, MeOD): δ (ppm) = 7.65 (d, J = 8.36 Hz, 2H), 7.52-7.51 (m, 3H), 7.34-7.20 (m, 7H), 7.11 (d, J = 8.35 Hz, 2H), 3.78 (s, 3H), 3.68-3.65 (m, 2H), 3.46 (s, 2H), 3.29-3.26 (m, 2H), 3.22-3.16 (m, 2H), 3.00-2.96 (m, 2H), 2.59 (s, 3H), 2.44 (s, 3H), 2.19-2.16 (m, 2H), 1.76-1.65 (m, 2H). ^{13}C NMR (101 MHz, MeOD): δ = 173.04, 153.78, 143.03, 141.01, 138.00, 135.89, 131.70, 130.96, 130.76, 130.51, 130.01, 129.72, 128.35, 122.73, 58.94, 53.32, 51.62, 42.58, 36.07, 31.41, 28.96, 13.82, 9.73. HR-ESI-MS (m/z): $[M+H]^+$ calculated: 535.3117; found: 535.3190.

(E)-2-(3,5-dimethyl-4-((4-nitrophenyl)diazenyl)-1H-pyrazol-1-yl)-ethan-1-ol (11)
General Procedure 2 was followed, using compound previously synthesized compound **10**[2] to obtain **11** as an orange solid (98%). ^{1}H-NMR (400 MHz, $CDCl_3$): δ = 8.32 (d, J = 9.1 Hz, 2H), 7.87 (d, J = 9.0 Hz, 2H), 4.18 (t, J = 4.8 Hz, 2H), 4.07 (t, J = 4.8 Hz, 2H), 2.64 (s, 3H), 2.51 (s, 3H). ^{13}C-NMR (101 MHz, $CDCl_3$): δ = 157.17, 147.79, 143.38, 141.61, 135.80, 124.83, 122.47, 61.47, 50.56, 14.32, 10.08. ESI-MS (m/z): $[M+H]^+$ found: 290.1257.

(*E*)-2-(4-((4-aminophenyl)diazenyl)-3,5-dimethyl-1*H*-pyrazol-1-yl)ethan-1-ol (12)
Intermediate **11** (1.0 g, 3.5 mmol, 1.0 eq.) was dissolved in a solvent mixture of THF/H2O (3:1, 60 mL), and Na_2S (1.0 g, 12.8 mmol, 3.4 eq.) was then added. The resulting mixture was stirred at reflux for 4 h. Afterwards, the reaction mixture was cooled to room temperature and the organic solvent was removed *in vacuo*. Aqueous 1 M NaOH and EtOAc were then added and after separation of the aqueous layer, the organic layer was extracted with sat. $NaHCO_3$ solution (1 x 50 mL) and sat. NaCl solution (1 x 50 mL). The organic layer was dried with Na_2SO_4, filtered and the solvent removed *in vacuo*. The crude product was subjected to silica chromatography (MPLC), eluting at 100% DCM (0.1%) Et_3N to 20% MeOH/DCM (0.1% Et_3N) to obtain compound **12** (0.6 g, 2.31 mmol, 67%) as an orange solid. ^{1}H-NMR (400 MHz, DMSO-D_6): δ = 7.49 (d, J = 8.7 Hz, 2H), 6.62 (d, J = 8.8 Hz, 2H), 4.04 (t, J = 5.6 Hz, 2H), 3.71 (q, J = 5.5 Hz, 2H), 2.51 (s, 3H), 2.34 (s, 3H). ^{13}C-NMR (101 MHz, DMSO-D_6): δ = 150.83, 143.80, 139.89, 138.01, 133.79, 123.22, 113.40, 60.06, 50.91, 13.92, 9.47. ESI-MS (m/z): $[M+H]^+$ found: 260.1533.

***tert*-butyl (*E*)-4-((4-((1-(2-hydroxyethyl)-3,5-dimethyl-1*H*-pyrazol-4-yl)diazenyl)phenyl)amino) piperidine-1-carboxylate (13)**
A solution of intermediate **12** (0.7 g, 2.7 mmol, 1.05 eq.), commercially available 1-boc-4-piperidone (0.5 g, 2.6 mmol, 1.0 eq.) and AcOH (169 µL, 2.7 mmol, 1.0 eq.) in dichloroethane (50 mL) was stirred at room temperature. Afterwards, $NaHB(OAc)_3$ (0.8 g, 3.5 mmol, 1.0 eq.) was slowly added over 15 mins,

and the mixture was then stirred at room temperature for 24 h. The reaction mixture was diluted with EtOAc and the organic layer was extracted with 1 M NaOH (2 x 50 mL), sat. $NaHCO_3$ solution (1 x 50 mL) and sat. NaCl solution (1 x 50 mL). The organic layer was dried with Na_2SO_4, filtered and the solvent removed *in vacuo*. The crude product was subjected to silica chromatography (MPLC), eluting at 100% DCM (0.1% Et_3N) to 10% MeOH/DCM (0.1% Et_3N) to obtain compound **13** as a yellow solid (0.8 g, 0.2 mmol, 77%). ^{1}H-NMR (400 MHz, DMSO-D_6): δ = 7.54 (d, J = 8.9 Hz, 2H), 6.67 (d, J = 8.9 Hz, 2H), 4.04 (t, J = 5.6 Hz, 2H), 3.90-3.87 (m, 2H), 3.71 (t, J = 5.6 Hz, 2H), 3.01-2.84 (m, 2H), 2.51 (s, 3H), 2.34 (s, 3H), 1.91-188 (m, 2H), 1.41 (s, 9H), 1.30-1.22 (m, 2H). ^{13}C-NMR (101 MHz, DMSO-D_6): δ = 153.95, 149.28, 143.75, 139.91, 138.05, 133.84, 123.28, 112.01, 78.62, 60.06, 50.92, 48.60, 28.10, 13.93, 9.49. ESI-MS (m/z): $[M+H]^+$ found: 443.2784.

tert-butyl (_E_)-4-(_N_-(4-((1-(2-hydroxyethyl)-3,5-dimethyl-1_H_-pyrazol-4-yl)diazenyl)phenyl) propionamido)piperidine-1-carboxylate (14)

Compound **13** (1.1g, 2.5 mmol, 1.0 eq.) was dissolved in anhydrous toluene (35 mL) under argon atmosphere. DMAP (0.62 g, 5.1 mmol, 2.0 eq), propionic anhydride (0.65 mL, 5.1 mmol, 2 eq.) and Et_3N (1.8 mL, 12.7 mmol, 5.0 eq.) were added and the mixture was stirred at room temperature for 16 h. The solvent was removed *in vacuo*, and the crude product was subjected to silica chromatography (MPLC), eluting at 100% PE to 90% EtOAc /PE to obtain **14** as a slightly yellow-coloured oil (0.1 g, 0.31 mmol, 10%). ^{1}H-NMR (400 MHz, $CDCl_3$): δ = 7.66 (d, J = 7.9 Hz, 2H), 6.61 (d, J = 7.8 Hz, 2H), 4.41 (t, J = 5.3 Hz, 2H), 4.26 (t, J = 5.3 Hz, 2H), 3.95-4.09 (m, 2H), 3.51-3.46 (m, 1H), 2.93 (t, J = 11.9 Hz, 2H), 2.54 (s, 3H), 2.46 (s, 3H), 2.32-2.26 (m, 2H), 2.08-2.03 (m, 2H), 1.45 (s, 9H), 1.39-1.31 (m, 2H), 1.10-1.06 (m, 3H). ^{13}C-NMR (101 MHz, $CDCl_3$): δ = 174.13, 154.85, 148.29, 145.68, 142.74, 137.92, 134.90, 123.81, 112.84, 79.80, 77.48, 77.16, 76.84, 62.88, 50.12, 47.49, 32.30, 28.50, 27.44, 13.87, 9.86, 9.02. ESI-MS (m/z): $[M+H]^+$ found: 499.3028.

(_E_)-_N_-(4-((1-(2-hydroxyethyl)-3,5-dimethyl-1_H_-pyrazol-4-yl)diazenyl)phenyl)-_N_-(piperidin-4-yl) propionamide (15)

Intermediate **14** (196 mg, 0.39 mmol, 1 eq.) was dissolved in a solvent mixture of DCM/THF (1:1). Afterwards, 4N HCl in dioxane (5 mL) was added and the mixture was stirred at room temperature for 1.5 h. The solvent was removed *in vacuo* and the crude product was then purified by reverse phase column chromatography (solvent A: H_2O [0.05 Vol% TFA], solvent B: MeCN; gradient A/B: 0-20 min: 90/10 to 2/98, 20-25 min: 2/98). After lyophilization, compound **15** was obtained as a white solid (120 mg, 0.3 mmol, 77%). ^{1}H-NMR (400 MHz, DMSO-D_6): δ = 7.56 (d, J = 8.8 Hz, 2H), 6.71 (d, J = 8.8 Hz, 2H), 4.36-4.33 (m, 2H), 4.29-4.26 (m, 2H), 3.66-3.60 (m, 1H), 3.33-3.30 (m, 2H), 3.06-2.98 (m, 2H), 2.51 (s, 3H), 2.34 (s, 3H), 2.28 (q, J = 7.5 Hz, 2H), 2.08-2.05 (m, 2H), 1.64-1.56 (m, 2H), 0.98 (t, J = 7.5 Hz, 3H). ^{13}C-NMR (101 MHz, DMSO-D_6): δ = 173.32, 149.10, 143.82, 140.33, 137.88, 133.93, 123.33, 111.99, 62.31, 47.14, 46.19, 42.07, 28.31, 26.67, 13.86, 9.26, 8.84. ESI-MS (m/z): $[M+H]^+$ found: 399.2517.

(_E_)-_N_-(4-((1-(2-hydroxyethyl)-3,5-dimethyl-1_H_-pyrazol-4-yl)diazenyl)phenyl)-_N_-(1-phenethyl piperidin-4-yl)propionamide (2)

Compound **15** (1.6 g, 4.0 mmol, 1.0 eq.) was dissolved in dichloroethane (100 mL) and stirred at room temperature. Phenylacetaldehyde (0.72 g, 6.0 mmol, 1.5 eq.) and $NaHB(OAc)_3$ (7.7 g, 36 mmol, 6.0 eq.) were added and the mixture was stirred under argon atmosphere for 16 h. The reaction mixture was diluted with EtOAc and the organic mixture was extracted with 1 M NaOH (1 x 100 mL), sat. $NaHCO_3$ (1 x 100 mL) and sat. NaCl solution (1 x 100 mL). The organic layer was dried with Na_2SO_4, filtered and the solvent was removed *in vacuo*. The crude product was purified silica chromatography (MPLC), eluting at 100% DCM to 8% MeOH/DCM to afford target compound **2** as an orange solid (0.9 g, 1.8 mmol, 45%). ^{1}H-NMR (400 MHz, MeOD): δ = 7.62 (d, J = 8.8 Hz, 2H), 7.28-7.24 (m, 2H), 7.19-7.17 (m, 3H), 6.66 (d, J = 8.9 Hz, 2H), 4.39 (t, J = 5.2 Hz, 2H), 4.27 (t, J = 5.2 Hz, 2H), 3.38-3.33 (m, 1H), 3.00-2.97 (m, 2H), 2.81-2.77 (m, 2H), 2.61-2.57 (m, 2H), 2.54 (s, 3H), 2.42 (s, 3H), 2.28 (q, J = 7.6, 2H), 2.24-2.21 (m, 2H), 2.05-2.02 (m, 2H), 1.59-1.50 (m, 2H), 1.04 (t, J = 7.6, 3H). ^{13}C-NMR (101 MHz, MeOD): δ = 175.50, 151.19, 146.02, 143.22, 141.19, 139.47, 135.73, 129.66, 129.50, 127.19, 124.82, 113.42,

63.70, 61.59, 53.37, 50.45, 48.43, 34.18, 32.64, 28.07, 13.95, 9.86, 9.30. HR-ESI-MS (m/z): [*M*+H]$^+$ calculated: 503.3129; found: 503.3134.

***tert*-Butyl (4-(2-(2,4-dioxopentan-3-ylidene)hydrazineyl)benzyl)carbamate (17)**
General Procedure 1 was followed, using commercially available 4-[(N-Boc)-aminomethyl]aniline **16** (1.00 g, 4.50 mmol) to obtain **17** as a yellow solid (1.11 g, 3.33 mmol, 74%). ^{1}H NMR (400 MHz, $CDCl_3$): δ =7.38-7.30 (m, 4H), 4.30 (br s, 2H) 2.59 (s, 3H), 2.48 (s, 3H), 1.46 (s, 9H). ^{13}C NMR (101 MHz, $CDCl_3$): δ = 198.09, 197.21, 156.01, 140.89, 137.01, 133.36, 128.90, 116.56, 79.83, 44.28, 31.79, 28.53, 26.76. ESI-MS (m/z): [*M*+H]$^+$ found: 334.18.

***tert*-butyl (*E*)-(4-((1,3,5-trimethyl-1*H*-pyrazol-4-yl)diazenyl)benzyl)carbamate (18)**
General Procedure 2 was followed, using intermediate **17** (520 mg, 1.56 mmol) and methylhydrazine (82 µL, 1.56 mmol, 1.0 eq.) to obtain **18** as a yellow solid (520 mg, 1.51 mmol, 97%.). ^{1}H NMR (400 MHz, $CDCl_3$): δ (ppm) = 7.74 (d, *J* = 8.30 Hz, 2H), 7.36 (d, *J* = 8.21 Hz, 2H), 4.36 (br s, 2H), 3.78 (s, 3H), 2.57 (s, 3H), 2.49 (s, 3H), 1.47 (s, 9H). ^{13}C NMR (101 MHz, $CDCl_3$): δ =155.91, 152.95, 142.45, 140.13, 138.73, 135.12, 127.99, 121.99, 120.62, 77.23, 44.44, 28.42, 13.81, 9.98. ESI-MS (m/z): [*M*+H]$^+$ found: 344.2087.

(*E*)-(4-((1,3,5-trimethyl-1*H*-pyrazol-4 yl)diazenyl)phenyl)methanamine (19)
General Procedure 3 was followed, using intermediate **18** (510 mg, 1.49 mmol) to obtain **19** as an orange oil (358 mg, 1.47 mmol, 99%). ^{1}H NMR (400 MHz, MeOD): δ (ppm) = 7.82 (d, *J* = 8.42 Hz, 2H), 7.57 (d, *J* = 8.42 Hz, 2H), 4.18 (s, 2H), 3.78 (s, 3H), 2.60 (s, 3H), 2.45 (s, 3H). ^{13}C NMR (101 MHz, MeOD): δ =155.21, 143.16, 141.56, 136.01, 135.56, 130.85, 123.30, 119.68, 116.76, 43.96, 36.12, 13.89, 9.76. ESI-MS (m/z): [*M*+H]$^+$ found: 244.1557.

(*E*)-1-phenethyl-*N*-(4-((1,3,5-trimethyl-1*H*-pyrazol-4-yl)diazenyl)benzyl)piperidin-4-amine (20)
To a solution of 1-phenethyl-4-piperidone (298 mg, 1.47 mmol, 1.05 eq.) in DCE (30 mL), was added sequentially to **19** (340 mg, 1.40 mmol), $NaHB(OAc)_3$, (415 mg, 2.88 mmol, 1.4 eq.) and AcOH (0.08 mL, 1.40 mmol, 1.0 eq.). The bright orange reaction mixture was allowed to stir overnight at room temperature. After 15 h, the reaction mixture was diluted with EtOAc (2 x 10 mL), washed with 1M NaOH (1x 5 mL), sat. $NaHCO_3$ (1 x 5 mL), sat. aqueous NaCl (1 x 10 mL). The combined organic layers were dried (Na_2SO_4), filtered and concentrated *in vacuo*. The crude product was subjected to silica chromatography, eluting at 100% DCM (0.01% Et_3N) to 20% DCM/MeOH (0.01% Et_3N) to obtain **20** as an orange oil (205 mg, 0.48 mmol, 34%). ^{1}H NMR (400 MHz, $CDCl_3$): δ (ppm) = 7.76-7.73 (m, 3H), 7.44-7.39 (m, 3H), 7.31-7.27 (m, 1H), 7.23-7.16 (m, 2H), 3.93 (s, 2H), 3.88 (s, 1H), 3.77- 3.74 (m, 5H), 3.17-3.14 (m, 1H), 2.87-2.81 (m, 4H), 2.57 (s, 3H), 2.55 (s, 1H), 2.49-2.48 (m, 5H), 2.08-2.05 (m, 1H), 1.72-1.69 (m, 2H). ^{13}C NMR (101 MHz, MeOD): 155.20, 143.16, 141.55, 135.46, 131.78, 130.84, 129.92, 129.79, 128.76, 123.35, 123.32, 122.84, 73.49, 51.87, 44.50, 43.99, 36.13, 36.06, 31.81, 13.89, 13.84, 9.77. ESI-MS (m/z): [*M*+H]$^+$ found: 431.2918.

(*E*)-*N*-(1-phenethylpiperidin-4-yl)-*N*-(4-((1,3,5-trimethyl-1*H*-pyrazol-4-yl)diazenyl)benzyl) propionamide (3a)
To a solution of intermediate **20** (180 mg, 0.42 mmol) in dry DCM (6 mL), triethylamine (0.11 mL, 0.84 mmol, 2.0 eq.) was added, followed by propionyl chloride (0.73 mL, 0.84 mmol, 2.0 eq.). The reaction mixture was allowed to stir for 24 h under N_2 atmosphere. Water (5 mL) was then added, and the organic phase was extracted with DCM (2 x 5 mL). The pooled organic phase was washed with sat. $NaHCO_3$ (1 x 5 mL) and sat. aqueous NaCl (1 x 5 mL). The organic phase was then dried over Na_2SO_4 and concentrated. The crude residue was subjected to silica chromatography, eluting at 100% DCM to 10% MeOH/DCM. The product was further purified by preparative HPLC (solvent A: H_2O [0.05 Vol% TFA], solvent B: MeCN; gradient A/B: 0-20 min: 90/10 to 2/98, 20-25 min: 2/98), eluting at 65% MeCN, to obtain **3a** as an orange oil (21 mg, 0.04 mmol, 12%).^{1}H NMR (400 MHz, MeOD): δ (ppm) = 7.80-7.69 (m, 2H), 7.41-7.23 (m, 7H), 4.69 (s, 2H), 4.54-4.27 (m, 1H), 3.77 (s, 3H), 3.68-3.65 (m, 2H), 3.29-3.27

(m, 2H), 3.15-3.00 (m, 4H), 2.70-2.64 (m, 1H), 2.58 (s, 3H), 2.46-2.41 (m, 4H), 2.22-2.09 (m, 2H), 1.98-1.94 (m, 2H), 1.25-1.11 (m, 3H). ^{13}C NMR (101 MHz, MeOD): δ = 177.34, 154.39, 143.05, 141.19, 140.62, 137.46, 135.94, 129.98, 129.75, 128.44, 128.31, 127.93, 123.27, 122.89, 59.00, 53.47, 53.19, 52.35, 36.08, 31.42, 29.24, 28.25, 27.91, 27.59, 13.85, 9.82, 9.75. HR-ESI-MS (m/z): [*M*+H]$^+$ calculated: 487.3117; found: 487.3190.

***tert*-butyl(*E*)-(4-((1-(2-hydroxyethyl)-3,5-dimethyl-1*H*-pyrazol-4-yl)diazenyl)benzyl)carbamate (21)**
General procedure 2 was followed, using intermediate **17** (1.13 g, 3.39 mmol) to obtain **21** as a yellow solid (1.30 g, 3.48 mmol, 99%.). ^{1}H NMR (400 MHz, $CDCl_3$): δ = 7.72 (d, J = 8.6 Hz, 2H), 7.34 (d, J = 8.6 Hz, 2H), 4.35-4.33 (m, 2H), 4.15-4.10 (m, 2H), 4.05-4.00 (m, 2H), 2.58 (s, 3H), 2.47 (s, 3H), 1.46 (s, 9H). ^{13}C NMR (101 MHz, $CDCl_3$): 155.96, 152.80, 142.67, 140.37, 139.63, 134.89, 127.96, 122.04, 116.44, 79.65, 61.37, 50.27, 44.39, 28.42, 13.96, 9.85. δ = [*M*+H]$^+$ found: 374.2240.

(*E*)-2-(4-((4-(aminomethyl)phenyl)diazenyl)-3,5-dimethyl-1*H*-pyrazol-1-yl)ethan-1-ol (22)
General Procedure 3 was followed, using intermediate **21** to obtain compound **22** as yellow crystals (95%). ^{1}H NMR (400 MHz, $CDCl_3$): δ = 7.70 (d, J = 8.4 Hz, 2H), 7.32 (d, J = 8.6 Hz, 2H), 4.11 (t, J = 4.8 Hz, 2H), 3.99 (t, J = 4.9 Hz, 2H), 3.84 (s, 2H), 2.58 (s, 3H), 2.46 (s, 3H). ^{13}C NMR (101 MHz, $CDCl_3$): δ = 152.62, 144.39, 142.71, 139.77, 134.96, 127.62, 122.08, 61.24, 50.65, 46.16, 14.14, 9.96. ESI-MS (m/z): [*M*+H]$^+$ found: 274.15.

(*E*)-2-(3,5-dimethyl-4-((4-(((1-phenethylpiperidin-4-yl)amino)methyl)phenyl)diazenyl)-1*H*-pyrazol-1-yl)ethan-1-ol (23)
Intermediate **22** (600 mg, 1.61 mmol, 1.0 eq.), 1-phenethyl-4-piperidone (444 mg, 1.61 mmol, 1.0 eq.) and AcOH (0.13 mL, 1.61 mmol, 1.0 eq.) was dissolved in dichloroethane (80 mL). To this reaction mixture, $NaHB(OAc)_3$ (651 mg, 3.07 mmol, 1.9 eq.) was slowly added over 15 mins. The mixture was stirred at room temperature for 24 h. The reaction mixture was diluted with EtOAc (120 mL) and the organic layer was extracted with 1 M NaOH (100 mL), sat. aqueous $NaHCO_3$ solution (100 mL) and sat. aqueous NaCl solution (100 mL). The organic layer was dried with Na_2SO_4, filtered and the solvent was removed *in vacuo*. The crude product was subjected to silica chromatography, eluting at 100% DCM (0.01% Et_3N) to 20% DCM/MeOH (0.01% Et_3N) to obtain intermediate **23** as a yellow solid (270 mg, 0.59 mmol, 37%). ^{1}H NMR (400 MHz, $CDCl_3$): δ = 7.74 (d, J = 8.3 Hz, 2H), 7.40 (d, J = 8.3 Hz, 2H), 7.30-7.27 (m, 2H), 7.20-7.19 (m, 3H), 4.13 (t, J = 4.8 Hz, 2H), 4.02 (t, J = 4.9 Hz, 2H), 3.86 (s, 2H), 2.98-2.95 (m, 2H), 2.82-2.78 (m, 3H), 2.60 (s, 3H), 2.59-2.55 (m, 2H), 2.50 (s, 3H), 2.09-2.04 (m, 2H), 1.94-1.91 (m, 2H), 1.52-1.42 (m, 2H). ^{13}C NMR (101 MHz, $CDCl_3$): δ = 152.74, 142.81, 142.23, 140.53, 139.60, 135.03, 128.78, 128.64, 128.46, 126.09, 121.95, 61.34, 60.70, 54.11, 52.53, 50.49, 33.87, 32.74, 14.12, 9.97. ESI-MS (m/z): [*M*+H]$^+$ found: 461.3032.

(*E*)-*N*-(4-((1-(2-hydroxyethyl)-3,5-dimethyl-1*H*-pyrazol-4-yl)diazenyl)benzyl)-*N*-(1-phenethyl piperidin-4-yl)propionamide (3b)
Intermediate **23** (270 mg, 0.587 mmol, 1.0 eq.), DMAP (86.0 mg, 0.704 mmol, 1.2 eq.), propionic anhydride (90 µL, 0.704 mmol, 1.2 eq.) and DIPEA (0.5 mL, 2.93 mmol, 5 eq.) were dissolved in DCM (10 mL) and stirred at room temperature for 24 h. The solvent was removed *in vacuo* and the crude product was subjected to silica chromatography, eluting at 100% DCM to 15% MeOH/DCM. The product was further purified by preparative HPLC (solvent A: H_2O [0.05 Vol% TFA], solvent B: MeCN; gradient A/B: 0-20 min: 10/98, 20-25 min: 2/98; tR = 10 min). The resulting compound was dissolved in MeOH/H2O (9:1, 10 mL) and then KOH (164 mg, 2.93 mmol, 5.0 eq.) was added. The mixture was stirred for 16 h and then acidified with 2M HCl and extracted with DCM (3 x 50 mL). The combined organic layers were dried with Na_2SO_4, filtered and the solvent was removed *in vacuo*. The product was purified by preparative HPLC (solvent A: H_2O [0.05 Vol% TFA], solvent B: MeCN; gradient A/B: 0-20 min: 90/10 to 2/98, 20-25 min: 2/98), eluting at 70% MeCN, to obtain target compound **3b** as a yellow residue (20 mg, 0.038 mmol, 7%). ^{1}H NMR (400 MHz, $CDCl_3$): δ = 7.72 (d, *J* = 8.4 Hz, 2H), 7.30-7.27 (m, 2H), 7.25-7.19 (m, 5H), 4.89 (br s, 1H), 4.64 (s, 2H), 4.14 (t, *J* = 4.8 Hz, 2H), 4.03 (t, *J* = 4.8 Hz, 2H),

3.60-3.58 (m, 2H), 3.15 (br s, 4H), 2.78-2.75 (m, 2H), 2.58 (s, 3H), 2.51-2.42 (m, 5H), 2.35-2.28 (m, 2H), 2.80-1.77 (m, 2H), 1.14-1.09 (m, 3H). ^{13}C NMR (101 MHz, $CDCl_3$): δ = 175.37, 153.02, 142.90, 139.87, 136.07, 135.07, 129.11, 128.78, 127.44, 126.36, 122.39, 61.51, 58.65, 52.77, 50.47, 48.91, 46.36, 30.59, 27.37, 27.16, 26.42, 14.13, 9.99, 9.58. HR-ESI-MS (m/z): $[M+H]^+$ calculated: 517.3286; found: 517.3292.

(*E*)-2-(4-((4-(((*tert*-butoxycarbonyl)amino)methyl)phenyl)diazenyl)-3,5-dimethyl-1*H*-pyrazol-1-yl)ethyl 4-methylbenzenesulfonate (24)
To a solution of intermediate **21** (1.30 g, 3.48 mmol) in DCM (80 mL), Et_3N (1.46 mL, 10.4 mmol, 3.0 eq.) was added, followed by the dropwise addition of p-toluenesulfonyl chloride (0.73 g, 3.83 mmol, 1.1 eq.) at room temperature. After 16 h of stirring, water was added (60 mL), and the mixture was extracted with DCM (2 x 40 mL). The combined organic layers were dried (Na_2SO_4), filtered and concentrated *in vacuo*. The crude residue was subjected to silica chromatography, eluting at 100% DCM to 20% MeOH/DCM to obtain **24** as yellow crystals (1.40 mg, 2.65 mmol, 76%).^1H NMR (400 MHz, $CDCl_3$): δ = 7.75 (d, J = 8.29 Hz, 2H), 7.59 (d, J = 8.29 Hz, 2H), 7.39 (d, J = 8.13 Hz, 2H), 7.18 (d, J = 8.17 Hz, 2H), 4.43 (t, J = 4.94 Hz, 2H), 4.39-4.37 (m, 2H), 4.27 (t, J = 4.92 Hz, 2H), 2.54 (s, 3H), 2.35 (s, 3H), 2.34 (s, 3H), 1.48 (s, 9H). ^{13}C NMR (101 MHz, $CDCl_3$): δ = 156.04, 152.83, 145.18, 142.79, 140.68, 134.92, 132.22, 129.98, 128.16, 127.86, 122.45, 122.24, 77.36, 68.37, 47.70, 28.55, 21.78, 13.86, 9.89. ESI-MS (m/z): $[M+H]^+$ found: 528.23.

***tert*-butyl (*E*)-(4-((1-(2-azidoethyl)-3,5-dimethyl-1*H*-pyrazol-4-yl)diazenyl)benzyl)carbamate (25)**
Compound **24** (1.30 g, 2.46 mmol) was dissolved in anhydrous DMSO (55 mL). Afterwards, NaN_3 (641 mg, 9.86 mmol, 4.0 eq.) and NaI (369 mg, 2.46 mmol, 1.0 eq.) were added and the reaction mixture was heated to 65 °C for 24 h under N_2 atmosphere. The reaction was allowed to cool to room temperature, water (50 mL) was added and the mixture was extracted with DCM (2 x 30 mL). The combined organic layers were dried (Na_2SO_4), filtered and concentrated *in vacuo*. The crude residue was subjected to silica chromatography, eluting at 100% DCM to 20% MeOH/DCM to obtain **25** as a yellow oil (714 mg, 1.79 mmol, 72%). ^1H NMR (400 MHz, $CDCl_3$): δ = 7.74 (d, J = 8.33 Hz, 2H), 7.36 (d, J = 8.09 Hz, 2H), 4.36-4.35 (m, 2H), 4.17 (t, J = 5.68 Hz, 2H), 3.77 (t, J = 5.68 Hz, 2H), 2.62 (s, 3H), 2.50 (s, 3H), 1.46 (s, 9H). ^{13}C NMR (101 MHz, $CDCl_3$): δ = 156.04, 152.94, 143.30, 140.09, 135.21, 128.09, 122.19, 116.55, 77.37, 50.79, 47.84, 44.53, 28.53, 14.13, 9.94. ESI-MS (m/z): $[M+H]^+$ found: 399.2260.

(*E*)-(4-((1-(2-azidoethyl)-3,5-dimethyl-1*H*-pyrazol-4-yl)diazenyl)phenyl)methanamine (26)
General Procedure 3 was followed, using intermediate **25** (704 mg, 1.79 mmol) to obtain compound **26** as an orange oil (675 mg, 2.26 mmol, 99%). ^1H NMR (400 MHz, $CDCl_3$): δ = 7.76 (d, J = 8.32 Hz, 2H), 7.40 (d, J = 8.24 Hz, 2H), 4.16 (t, J = 5.67 Hz, 2H), 3.93 (s, 2H), 3.77 (t, J = 5.67 Hz, 2H), 2.62 (s, 3H), 2.51 (s, 3H), 1.81 (s, 2H). ^{13}C NMR (101 MHz, $CDCl_3$): δ = 152.61, 144.40, 143.28, 139.76, 135.15, 127.66, 122.05, 50.70, 47.74, 46.16, 14.10, 9.82. ESI-MS (m/z): $[M+H]^+$ found: 299.17.

(*E*)-*N*-(4-((1-(2-azidoethyl)-3,5-dimethyl-1*H*-pyrazol-4-yl)diazenyl)benzyl)-1-phenethylpiperidin-4-amine (27)
To a solution of 1-phenethylpiperidin-4-one (465 mg, 2.29 mmol, 1.05 eq.) in DCE (10 mL), was added sequentially to **26** (650 mg, 2.18 mmol), $NaHB(OAc)_3$, (646 mg, 3.05 mmol, 1.4 eq.) and AcOH (0.12 mL, 2.18 mmol, 1.0 eq.). The bright orange reaction mixture was allowed to stir overnight at room temperature. After 15 h, the reaction mixture was diluted with EtOAc (2 x 10 mL), washed with 1M NaOH (1x 5 mL), sat. $NaHCO_3$ (1 x 5 mL), sat. NaCl solution (1 x 10 mL). The combined organic layers were dried with Na_2SO_4, filtered and concentrated *in vacuo*. The crude residue was subjected to silica chromatography, eluting at 100% DCM to 10% MeOH/DCM to obtain **26** as an orange oil (680 mg, 1.40 mmol, 64%). ^1H NMR (400 MHz, $CDCl_3$): δ = 7.75 (d, J = 8.3 Hz, 2H), 7.43 (d, J = 8.3 Hz, 2H), 7.30-7.27 (m, 2H), 7.21-7.18 (m, 3H), 4.17 (t, J = 5.7 Hz, 2H), 3.89 (s, 2H), 3.77 (t, J = 5.7 Hz, 2H), 3.10-3.07 (m, 2H), 2.89-2.2.85 (m, 2H), 2.72-2.68 (m, 2H), 2.62 (s, 3H), 2.51 (s, 3H), 2.30-2.25 (m, 2H), 2.03 (s, 2H), 2.01 (br s, 1H), 1.67-1.61 (m, 2H), ^{13}C NMR (101 MHz, $CDCl_3$): δ = 152.94, 143.44, 139.97, 135.30,

128.90, 128.85, 128.64, 126.42, 122.10, 59.96, 51.65, 51.63, 50.83, 50.30, 47.88, 32.98, 31.34, 14.23, 9.96. ESI-MS (m/z): $[M+H]^+$ found: 486.3088.

E)-*N*-(4-((1-(2-azidoethyl)-3,5-dimethyl-1*H*-pyrazol-4-yl)diazenyl)benzyl)-*N*-(1-phenethylpiperidin-4-yl)propionamide (3c)

To a solution of **27** (660 mg, 1.36 mmol) in anhydrous DCM (15 mL), Et_3N (0.37 mL, 2.72 mmol, 2 eq.) was added, followed by propionyl chloride (0.24 mL, 2.72 mmol, 2.0 eq.). The reaction mixture was allowed to stir for 24 h under N_2 atmosphere at room temperature. Water was then added, and the organic phase was extracted with DCM (2 x 10 mL). The pooled organic phase was washed with sat. $NaHCO_3$ (1x 20 mL) and sat. NaCl solution (1 x 20 mL). The combined organic layers were dried (Na_2SO_4), filtered and concentrated *in vacuo*. The crude residue was subjected to silica chromatography, eluting at 100% DCM to 10% MeOH/DCM. The product was further purified by preparative HPLC (solvent A: H_2O [0.05 Vol% TFA], solvent B: MeCN; gradient A/B: 0-20 min: 90/10 to 2/98, 20-25 min: 2/98), eluting at 70% MeCN, to obtain **3c** as an orange oil (525 mg, 0.97 mmol, 71%). ^1H NMR (400 MHz, $CDCl_3$): δ = 7.77 (d, J = 8.3 Hz, 2H), 7.32-2.25 (m, 5H), 7.17 (d, J = 8.2 Hz, 2H), 4.93-4.87 (m, 1H), 4.61 (s, 2H), 4.19 (t, J = 5.6 Hz, 2H), 3.78 (t, J = 5.6 Hz, 2H), 3.67-3.64 (m, 2H), 3.18-3.14 (m, 2H), 3.06-3.02 (m, 2H), 2.79-2.74 (m, 2H), 2.63 (s, 3H), 2.51 (s, 3H), 2.37 (q, J = 7.3 Hz, 2H), 2.21-2.15 (m, 2H), 1.85-1.82 (m, 2H), 1.15 (t, J = 7.3 Hz, 3H). ^{13}C NMR (101 MHz, $CDCl_3$): δ = 175.46, 153.08, 143.46, 140.35, 139.13, 135.79, 135.22, 129.19, 128.72, 127.56, 126.30, 122.54, 58.52, 52.70, 50.81, 48.87, 47.83, 46.30, 30.60, 27.16, 26.50, 14.04, 9.94, 9.63. HR-ESI-MS (m/z): $[M+H]^+$ calculated: 542.3284; found: 542.3357. IR: ν [cm^{-1}]: 2933, 2803, 2102, 1733, 1640, 1558, 1502, 1409, 1375, 1282, 1233, 1200, 1118, 1077, 1033, 999, 936, 824, 749.

(*E*)-1-(phenyldiazenyl)piperidin-4-one (29)

To a stirred solution of aniline **28** (1.0 g, 10.7 mmol, 1.0 eq.) dissolved in a mixture of acetonitrile and water (2:1, 15 mL), 12 M HCl (3.6 mL, 43.0 mmol, 4.0 eq.) was added dropwise at 0 °C. In dark conditions, the reaction mixture was further cooled to -5 °C and an aqueous solution of $NaNO_2$ (1.1 g, 34 mmol, 1.5 eq.) dissolved in a minimal amount of water was added dropwise and stirred for 0.5 h. At 0 °C, the reaction mixture was added slowly to a stirred solution of 4-piperidone (3.1 mL, 26.9 mmol, 2.5 eq.) and potassium carbonate (7.73 g, 55.9 mmol, 5.2 eq.) in a 2:1 mixture of acetonitrile and water (60 mL). The reaction mixture was allowed to warm to room temperature and stirred for 1 h. The mixture was then extracted with DCM (2 x 30 mL). The combined organic layer was washed with sat. $NaHCO_3$ (1 x 30 mL), dried with Na_2SO_4, and solvent removed *in vacuo*. The crude residue was subjected to silica chromatography in dark conditions, eluting at 100% DCM to 10% MeOH/DCM to obtain **29** as a red oil (1.127 g, 5.55 mmol, 52%). ESI-MS (m/z): $[M+H]^+$ found: 204.1136. Compound characterization matches that previously reported.[6]

(*E*)-*N*-phenyl-1-(phenyldiazenyl)piperidin-4-amine (30)

To a solution of **29** (1.05 g, 5.19 mmol, 1.05 eq.) in DCE (30 mL), was added sequentially aniline (0.50 mL, 5.45 mmol, 1.05 eq.), $NaHB(OAc)_3$ (1.54 g, 7.27 mmol, 1.4 eq.) and AcOH (0.30 mL, 5.19 mmol, 1.0 eq.). The bright orange reaction mixture was allowed to stir overnight at room temperature. After 15 h, the reaction mixture was diluted with EtOAc (2 x 10 mL), washed with 1M NaOH (1x 5 mL), sat. $NaHCO_3$ (1 x 5 mL), brine (1 x 10 mL). The combined organic layers were dried (Na_2SO_4), filtered and concentrated *in vacuo*. The crude residue was subjected to silica chromatography, eluting at 100% DCM (0.01% Et_3N) to 20% DCM/MeOH (0.01% Et_3N) to obtain **30** as a dark orange residue (453 mg, 1.62 mmol, 31%).^1H NMR (400 MHz, $CDCl_3$): δ (ppm) = 7.55-7.53 (m, 2H), 7.45-7.41 (m, 2H), 7.30-7.28 (m, 2H), 7.25-7.21 (m, 1H), 6.83-6.80 (m, 1H), 6.76-6.70 (m, 2H), 4.54-4.50 (m, 2H), 3.68-3.62 (m, 1H), 3.40-3.35 (m, 2H), 2.23-2.24 (m, 2H), 1.66-1.56 (m, 2H). ^{13}C NMR (101 MHz, $CDCl_3$): δ = 150.56, 146.72, 129.51, 129.36, 128.98, 126.15, 120.75, 117.77, 115.18, 113.48, 50.00, 31.63. ESI-MS (m/z): $[M+H]^+$ found: 281.1759.

(*E*)-*N*-phenyl-*N*-(1-(phenyldiazenyl)piperidin-4-yl)propionamide (4)

To a solution of intermediate **30** (360 mg, 0.42 mmol) in dry DCM (6 mL), triethylamine (0.35 mL, 2.57 mmol, 2.0 eq.) was added, followed by propionyl chloride (0.22 mL, 2.57 mmol, 2.0 eq.). The reaction mixture was allowed to stir for 24 h under N_2 atmosphere. Water (5 mL) was then added, and the organic phase was extracted with DCM (2 x 5 mL). The pooled organic phase was washed with sat. $NaHCO_3$ (1 x 5 mL) and brine (1 x 5 mL). The organic phase was then dried over Na_2SO_4 and concentrated. The crude residue was subjected to silica chromatography, eluting at 100% DCM to 10% MeOH/DCM to obtain **4** as a red oil (220 mg, 0.654 mmol, 51%). ^1H NMR (400 MHz, $CDCl_3$): δ (ppm) = 7.28-7.24 (m, 5H), 7.19-7.16 (m, 2H), 7.03-6.99 (m, 1H), 6.96-6.94 (m, 2H), 4.85-4.78 (m, 1H), 4.47-4.44 (m, 2H), 3.03-2.97 (m, 2H), 1.85-1.80 (m, 4H), 1.35-1.24 (m, 2H), 0.91 (t, *J* = 7.44 Hz, 3H). ^{13}C NMR (101 MHz, $CDCl_3$): δ = 173.67, 150.43, 138.65, 130.25, 129.55, 128.86, 128.62, 126.00, 120.62, 52.01, 43.66, 30.00, 28.53, 9.64. ESI-MS (m/z): $[M+H]^+$ found: 337.2020.

2.3. Analytical HPLC Chromatograms for Purity Determination in DMSO

Compound 1:

Detection at 220 nm: >99% purity

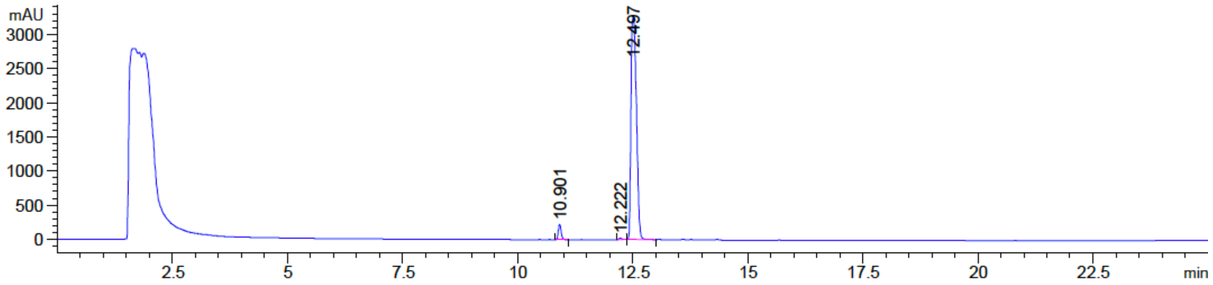

Signal 2: DAD1 B, Sig=220,4 Ref=off

Peak #	RetTime [min]	Type	Width [min]	Area [mAU*s]	Height [mAU]	Area %
1	10.901	BB	0.0655	944.72748	222.49606	3.3854
2	12.222	BB	0.0663	81.93166	19.00448	0.2936
3	12.497	BV R	0.1340	2.68792e4	3262.71216	96.3210

*Peak 1 and Peak 3 are *cis* and *trans* isomers of compound **1**, respectively.

Detection at 254 nm: 99% purity

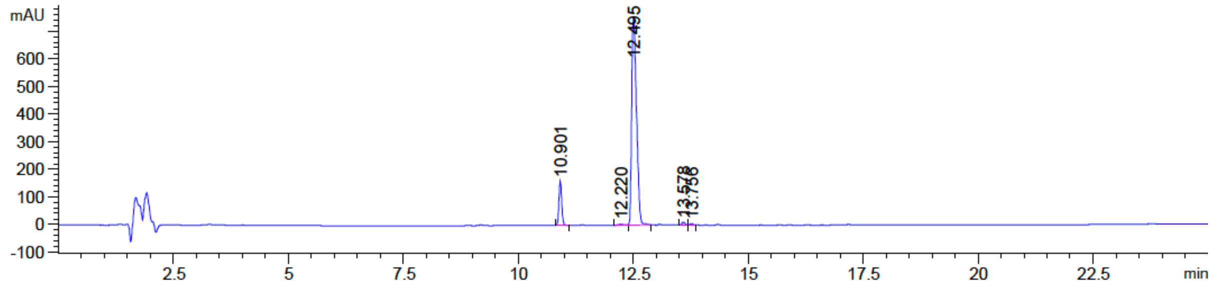

Signal 1: DAD1 A, Sig=254,4 Ref=off

Peak #	RetTime [min]	Type	Width [min]	Area [mAU*s]	Height [mAU]	Area %
1	10.901	VB	0.0655	673.46973	158.75671	10.7275
2	12.220	BV E	0.0788	21.58519	4.00680	0.3438
3	12.495	VV R	0.1131	5518.78857	754.05664	87.9069
4	13.578	BV	0.0706	44.29095	9.82660	0.7055
5	13.756	VB	0.0675	19.86117	4.68273	0.3164

*Peak 1 and Peak 3 are *cis* and *trans* isomers of compound **1**, respectively.

Compound 2:

Detection at 220 nm: >99% purity

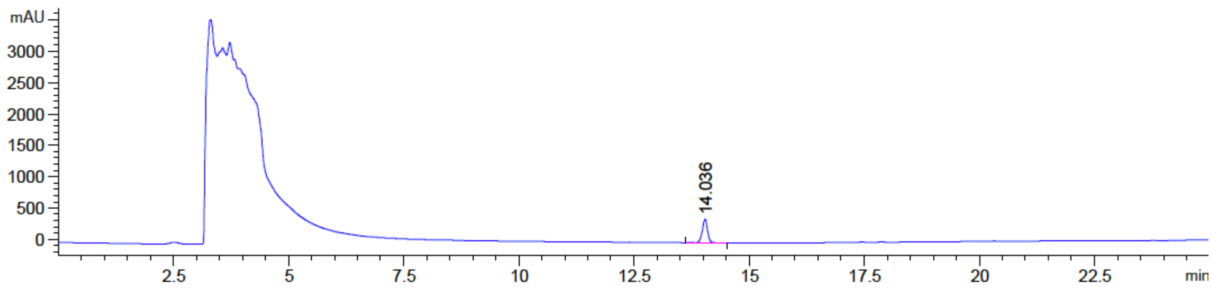

Signal 2: DAD1 B, Sig=220,4 Ref=off

Peak #	RetTime [min]	Type	Width [min]	Area [mAU*s]	Height [mAU]	Area %
1	14.036	VB R	0.1192	2952.95972	376.73669	100.0000

Detection at 254 nm: 97% purity

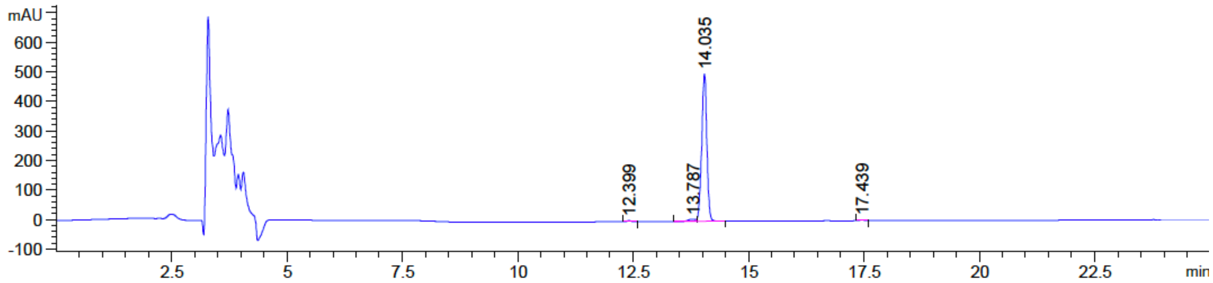

Signal 1: DAD1 A, Sig=254,4 Ref=off

Peak #	RetTime [min]	Type	Width [min]	Area [mAU*s]	Height [mAU]	Area %
1	12.399	BB	0.0947	24.90855	4.11141	0.6172
2	13.787	BV E	0.1745	76.92455	7.21064	1.9061
3	14.035	VB R	0.1194	3913.41895	499.68018	96.9718
4	17.439	BB	0.0987	20.37437	3.26880	0.5049

Compound 3a:
Detection at 220 nm: 98% purity

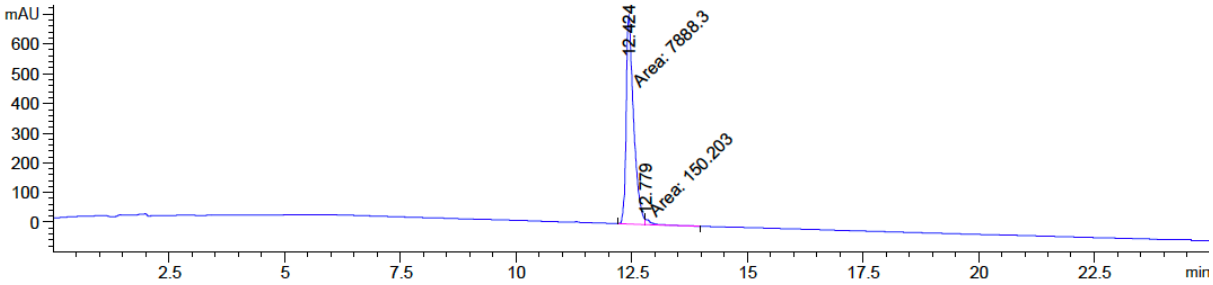

Signal 2: DAD1 B, Sig=220,4 Ref=off

Peak #	RetTime [min]	Type	Width [min]	Area [mAU*s]	Height [mAU]	Area %
1	12.424	MF	0.1877	7888.30420	700.51556	98.1315
2	12.779	FM	0.1446	150.20280	17.31518	1.8685

Detection at 254 nm: 98% purity

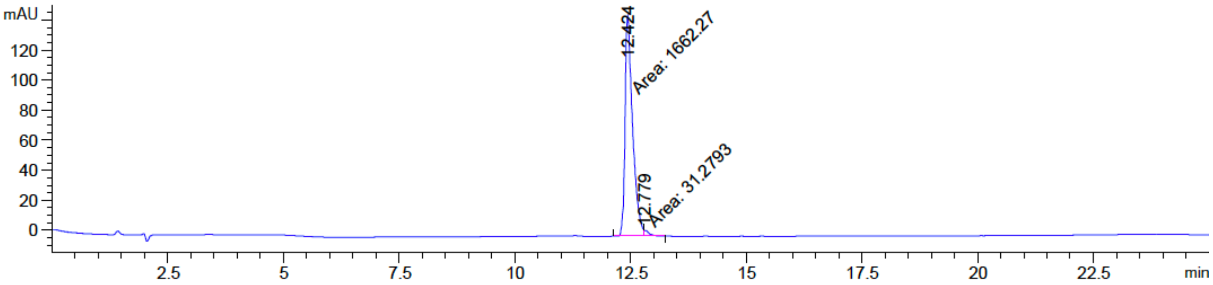

Signal 1: DAD1 A, Sig=254,4 Ref=off

Peak #	RetTime [min]	Type	Width [min]	Area [mAU*s]	Height [mAU]	Area %
1	12.424	MF	0.1892	1662.26672	146.42648	98.1530
2	12.779	FM	0.1320	31.27930	3.95037	1.8470

Compound 3b:
Detection at 220 nm: >99% purity

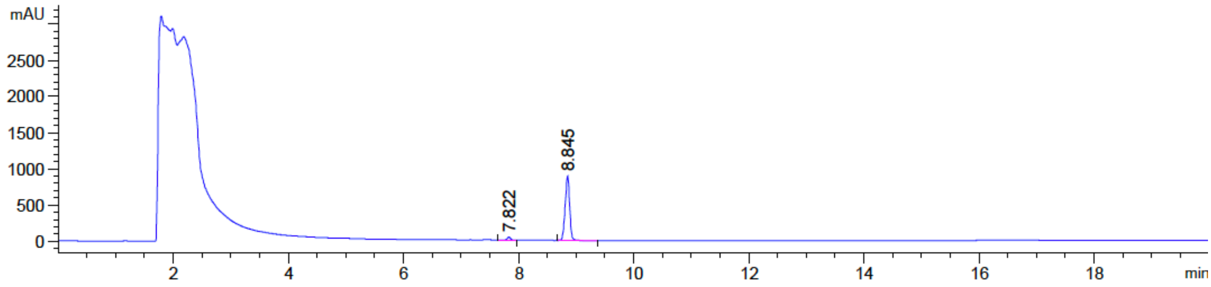

Peak #	RetTime [min]	Type	Width [min]	Area [mAU*s]	Height [mAU]	Area %
1	7.822	VB R	0.0660	180.70978	42.14679	3.5952
2	8.845	BV R	0.0832	4845.67578	894.04822	96.4048

*Peak 1 and Peak 2 are *cis* and *trans* isomers of compound **3b**, respectively.

Detection at 254 nm: 96% purity

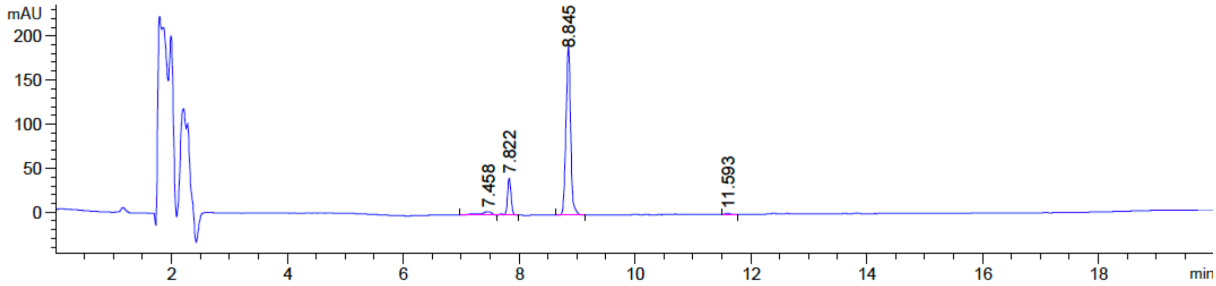

Signal 1: DAD1 A, Sig=254,4 Ref=off

Peak #	RetTime [min]	Type	Width [min]	Area [mAU*s]	Height [mAU]	Area %
1	7.458	BV E	0.2028	55.41312	4.02104	4.1769
2	7.822	VB R	0.0651	182.76895	42.03464	13.7766
3	8.845	BB	0.0855	1082.79150	192.77330	81.6179
4	11.593	BB	0.0737	5.68597	1.19136	0.4286

*Peak 2 and Peak 3 are *cis* and *trans* isomers of compound **3b**, respectively.

Compound 3c:

Detection at 220 nm: 96% purity

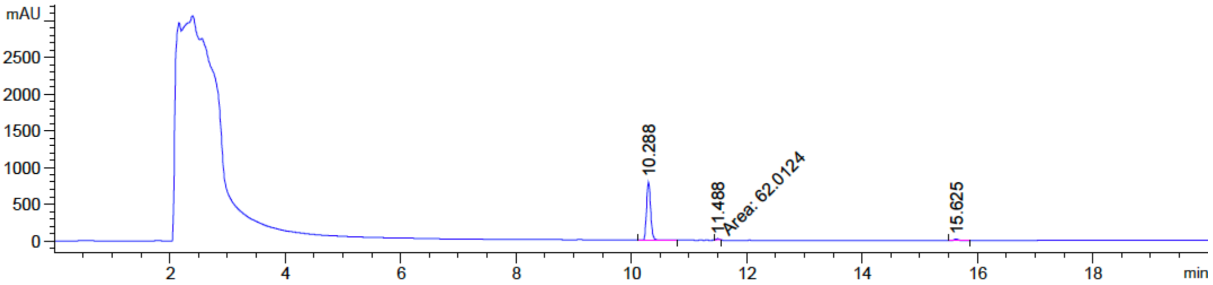

Signal 2: DAD1 B, Sig=220,4 Ref=off

Peak #	RetTime [min]	Type	Width [min]	Area [mAU*s]	Height [mAU]	Area %
1	10.288	BV R	0.0746	3870.74390	797.74713	95.5414
2	11.488	MM	0.0604	62.01241	17.10340	1.5306
3	15.625	BB	0.0811	118.62248	22.65009	2.9280

Detection at 254 nm: 98% purity

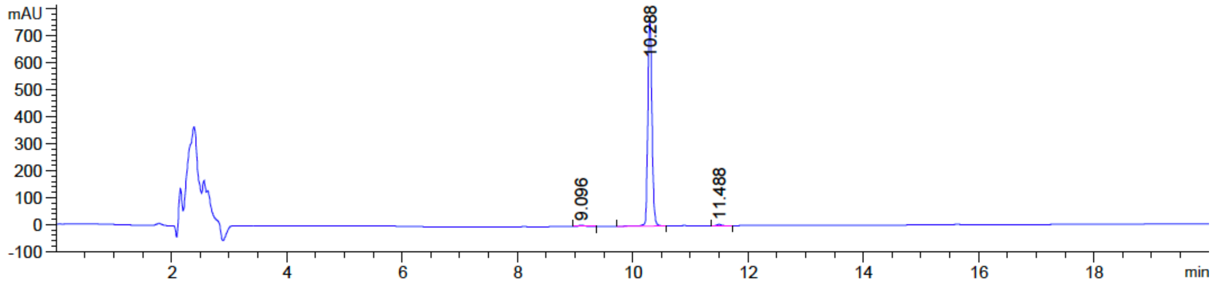

Signal 1: DAD1 A, Sig=254,4 Ref=off

Peak #	RetTime [min]	Type	Width [min]	Area [mAU*s]	Height [mAU]	Area %
1	9.096	BB	0.1030	35.88100	5.17085	0.9274
2	10.288	VB R	0.0747	3807.14722	779.84143	98.4018
3	11.488	BB	0.0789	25.95119	4.96788	0.6708

PF2:

Detection at 220 nm: 99% purity

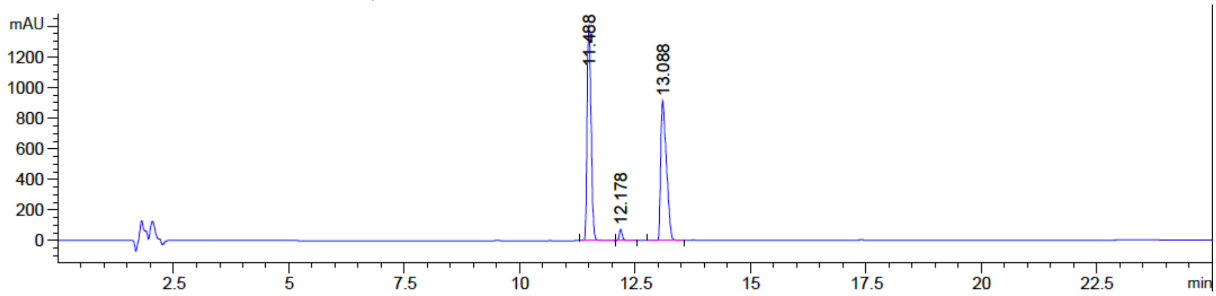

Signal 2: DAD1 B, Sig=220,4 Ref=off

Peak #	RetTime [min]	Type	Width [min]	Area [mAU*s]	Height [mAU]	Area %
1	11.488	VV R	0.0939	8630.08594	1435.03577	22.2835
2	12.178	BV R	0.0697	185.58174	40.29873	0.4792
3	13.091	VV R	0.1446	2.99129e4	3267.29736	77.2373

*Peak 1 and Peak 3 are *cis* and *trans* isomers of compound **PF2**, respectively.

Detection at 254 nm: 98% purity

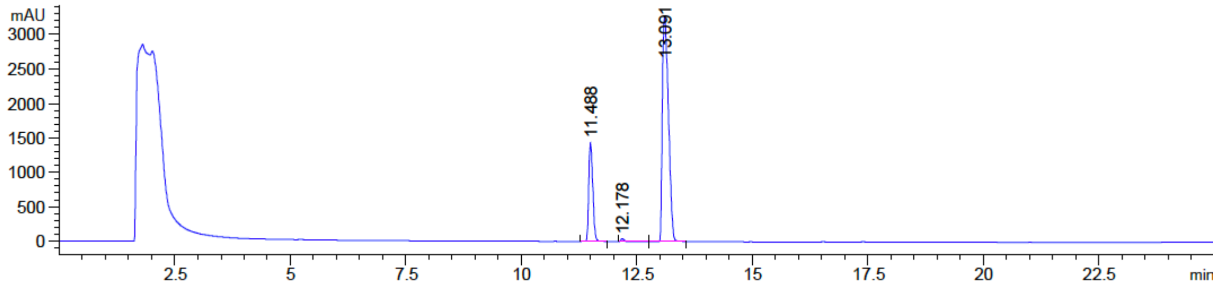

Signal 1: DAD1 A, Sig=254,4 Ref=off

Peak #	RetTime [min]	Type	Width [min]	Area [mAU*s]	Height [mAU]	Area %
1	11.488	VV R	0.0937	8408.31250	1404.99841	50.4837
2	12.178	BV R	0.0699	340.14166	74.24352	2.0422
3	13.088	VV R	0.1267	7907.04053	913.79034	47.4741

*Peak 1 and Peak 3 are *cis* and *trans* isomers of compound **PF2**, respectively.

3. Supplementary Photophysical Information

3.1. General Information

For determination of thermal equilibrium and isomer spectra, as well as determination of cycle performance and thermal half-life, UV/Vis absorption spectroscopy was employed. UV/Vis absorption spectroscopy was performed using Agilent 8453 spectrometer or Agilent Varian Cary 50 spectrometer, in 10 mm quartz cuvettes. To determine PSS values at the respective isosbestic points of compounds (50 μM), analytical HPLC was performed using Agilent 1220 Infinity LC System (column: Phenomenex Luna, 3 μM C18(2), 100 Å 150 x 2.0 mm; flow rate of 0.3 mL/min at 20 °C; solvent A: Milli-Q water with 0.05 wt% TFA; solvent B: MeCN). LED light sources for irradiation: λ=265 nm (Nikkiso, VPC131, 350 mA, 6.3 V), λ=285 nm (Nikkiso, VPS173, 500 mA, 6.0 V), λ=365 nm (Seoul Viosys, CUN66A1B, 700 mA, 3.6 V), λ=400 nm (Luxeon LHUV-0400-0450, SZ-01-S2, 1000 mA, 3.2 V), λ=420 nm (Mouser, L1F3-U410200012000, 700 mA, 3.4 V), λ=451 nm (LEDTECH, Oslon SSL 80, LDCQ7P-2U3U, 700 mA, 3.2 V), λ=528 nm (LEDTECH, Oslon SSL 80, LDCQ7P-2U3U, 700 mA, 3.5 V), λ=645 nm (LEDTECH, Oslon SSL 80, LHCP7P-2T3T, 700 mA, 2.6 V). The details of these light sources are based on the supplier specifications upon purchase.

3.2. UV/Vis Absorption Spectroscopy, Cycle Performance and Thermal Stabilities

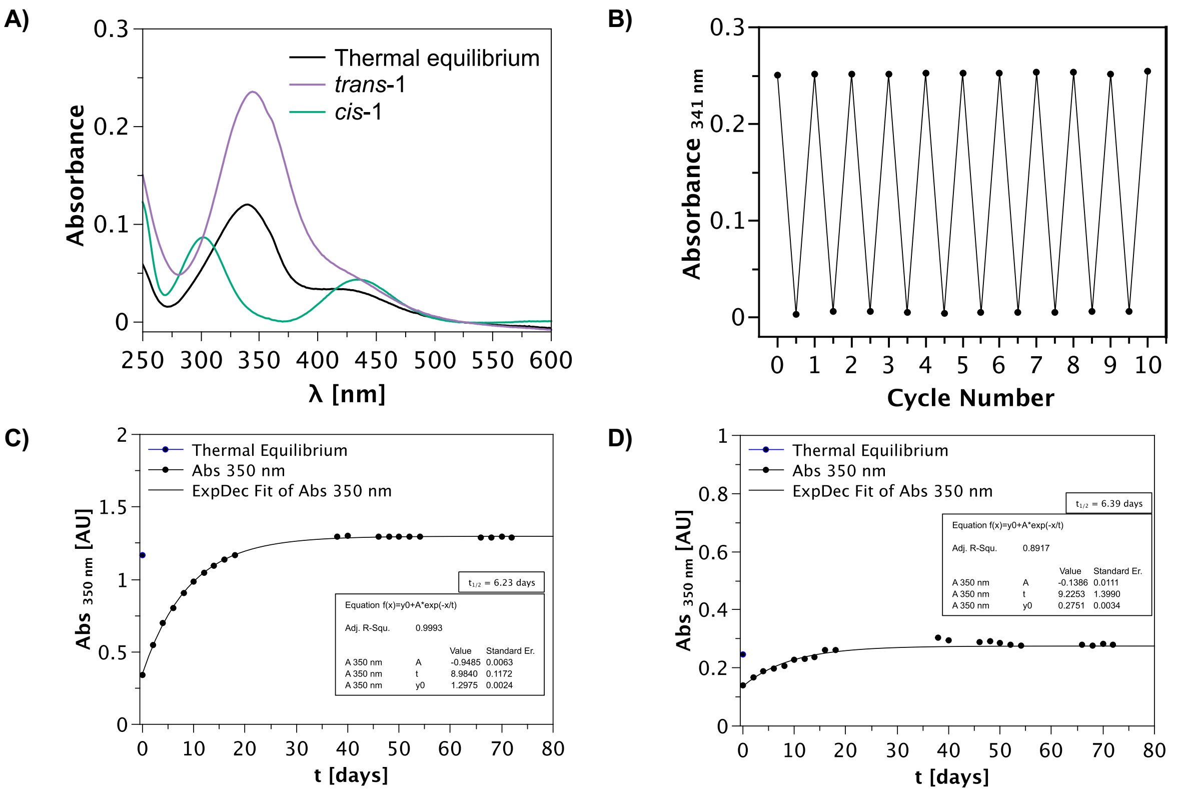

SI Figure 1. Compound **1** (20 µM) in buffer solution (TrisHCl Buffer, pH 7.5) + 0.2% DMSO at 25 ºC. A) UV/Vis absorption spectra data of thermal equilibrium, *trans* isomer and *cis* isomer. The *cis* isomer was accessed via irradiation with 365 nm, while the *trans* isomer was obtained with 528 nm irradiation. B) Cycle performance upon alternating irradiation of 365 nm and 528 nm. Data points were recorded at the absorbance maximum of the *trans* isomer (341 nm). C) Thermal half-life of compound **1** (150 µM) measured at 27 °C in DMSO. D) Thermal half-life of compound **1** (50 µM) measured at 27 °C in buffer solution (TrisHCl Buffer, pH 7.5) + 0.5% DMSO.

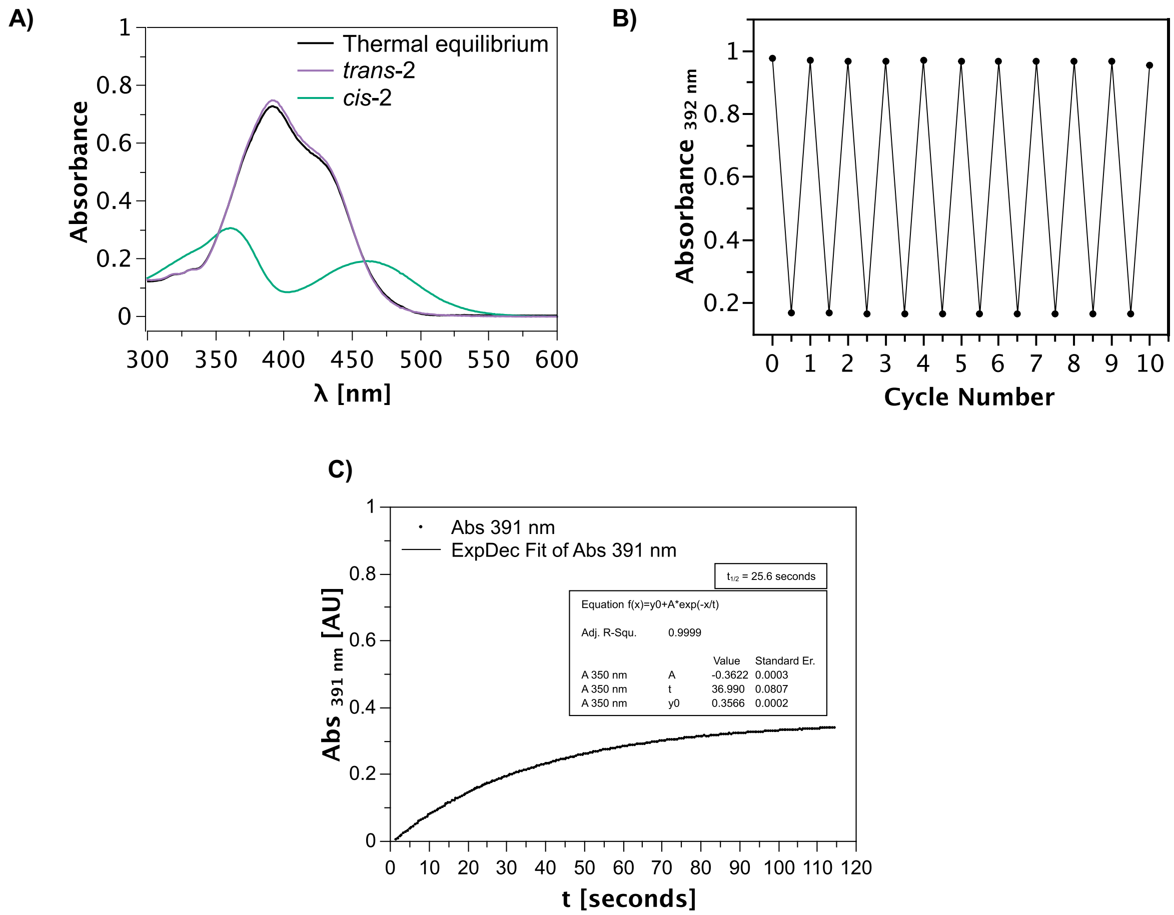

SI Figure 2. Compound **2** (50 µM) in DMSO at 25 ºC. A) UV/Vis absorption spectra data of thermal equilibrium, *trans* isomer and *cis* isomer. The *cis* isomer was accessed via continuous irradiation with 400 nm, while the *trans* isomer was obtained via irradiation with 528 nm. B) Cycle performance upon alternating irradiation of 365 nm and darkness. Data points were recorded at the absorbance maximum of the *trans* isomer (392 nm). C) Thermal half-life of compound **1** (50 µM) measured at 27 °C in DMSO. UV/Vis trace obtained every 0.5 seconds.

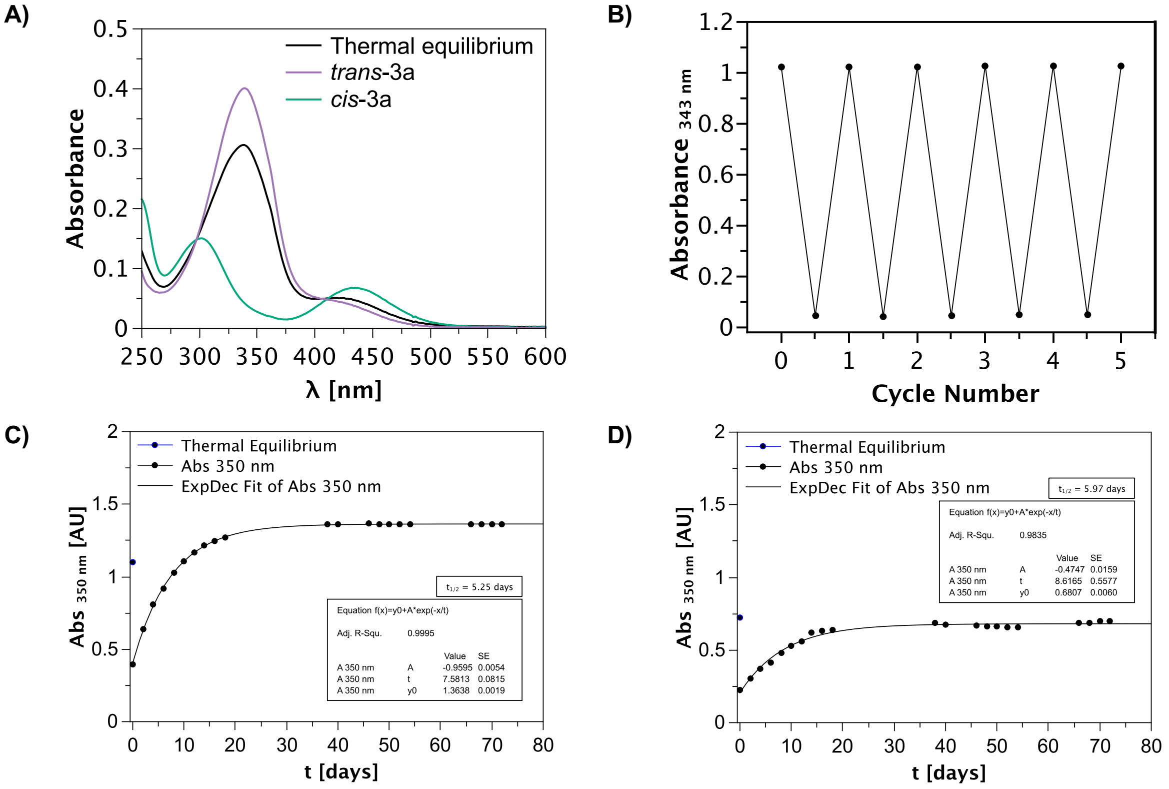

SI Figure 3. Compound **3a** (20 µM) in buffer solution (TrisHCl Buffer, pH 7.5) + 0.2% DMSO at 25 ºC. A) UV/Vis absorption spectra data of thermal equilibrium, *trans* isomer and *cis* isomer. The *cis* isomer was accessed via irradiation with 365 nm, while the *trans* isomer was obtained with 528 nm irradiation. B) Cycle performance upon alternating irradiation of 365 nm and 528 nm. Data points were recorded at the absorbance maximum of the *trans* isomer (343 nm). C) Thermal half-life of compound **1** (150 µM) measured at 27 °C in DMSO. D) Thermal half-life of compound **1** (150 µM) measured at 27 °C in buffer solution (TrisHCl Buffer, pH 7.5) + 0.5% DMSO.

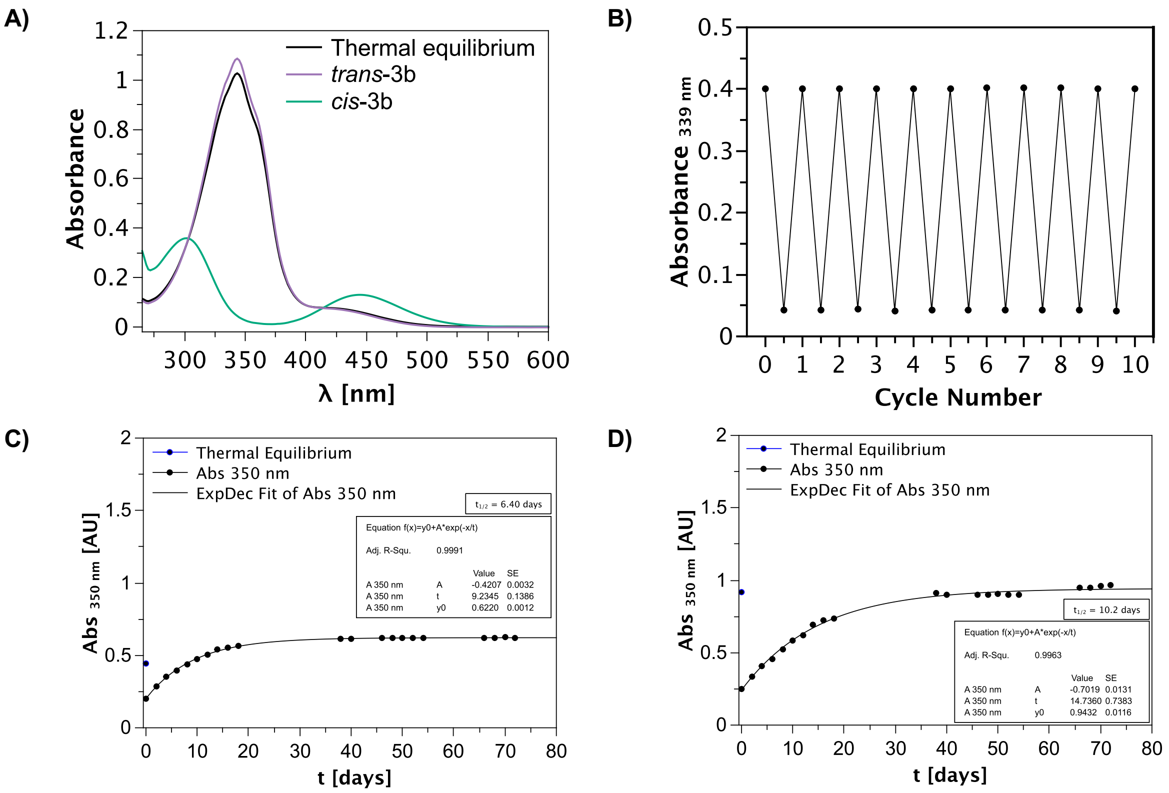

SI Figure 4. Compound **3b** (50 µM) in buffer solution (TrisHCl Buffer, pH 7.5) + 0.2% DMSO at 25 ºC. A) UV/Vis absorption spectra data of thermal equilibrium, *trans* isomer and *cis* isomer. The *cis* isomer was accessed via irradiation with 365 nm, while the *trans* isomer was obtained with 528 nm irradiation. B) Cycle performance upon alternating irradiation of 365 nm and 528 nm. Data points were recorded at the absorbance maximum of the *trans* isomer (339 nm). C) Thermal half-life of compound **1** (50 µM) measured at 27 °C in DMSO. D) Thermal half-life of compound **1** (150 µM) measured at 27 °C in buffer solution (TrisHCl Buffer, pH 7.5) + 0.5% DMSO.

A)

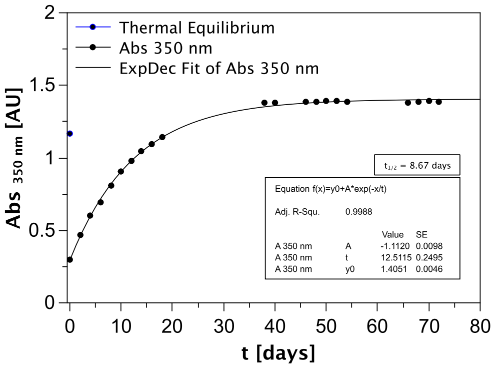

B)

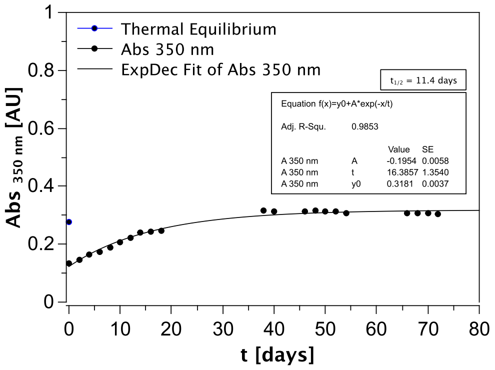

SI Figure 5. Compound **1** (20 µM) in buffer solution (TrisHCl Buffer, pH 7.5) + 0.2% DMSO at 25 ºC. A) UV/Vis absorption spectra data of thermal equilibrium, *trans* isomer and *cis* isomer. The *cis* isomer was accessed via irradiation with 365 nm, while the *trans* isomer was obtained with 528 nm irradiation. B) Cycle performance upon alternating irradiation of 365 nm and 528 nm. Data points were recorded at the absorbance maximum of the *trans* isomer (341 nm). C) Thermal half-life of compound **1** (150 µM) measured at 27 °C in DMSO. D) Thermal half-life of compound **1** (50 µM) measured at 27 °C in buffer solution (TrisHCl Buffer, pH 7.5) + 0.5% DMSO.

3.3. Analytical HPLC Chromatogram for PSS Determination

Compound 1 (50 µM solution in TrisHCl Buffer + 0.5% DMSO, pH 7.5, injection volume 10 µL):

Irradiation with λ = 528 nm for conversion from cis isomer to trans isomer.
Detection at 297 nm: t_R *cis* isomer = 11.018 min (1%), t_R *trans* isomer = 12.176 min (99%).

Figures of analytical HPLC trace, solvent gradient (acetonitrile/H2O + 0.01% TFA), pressure profile and data analysis:

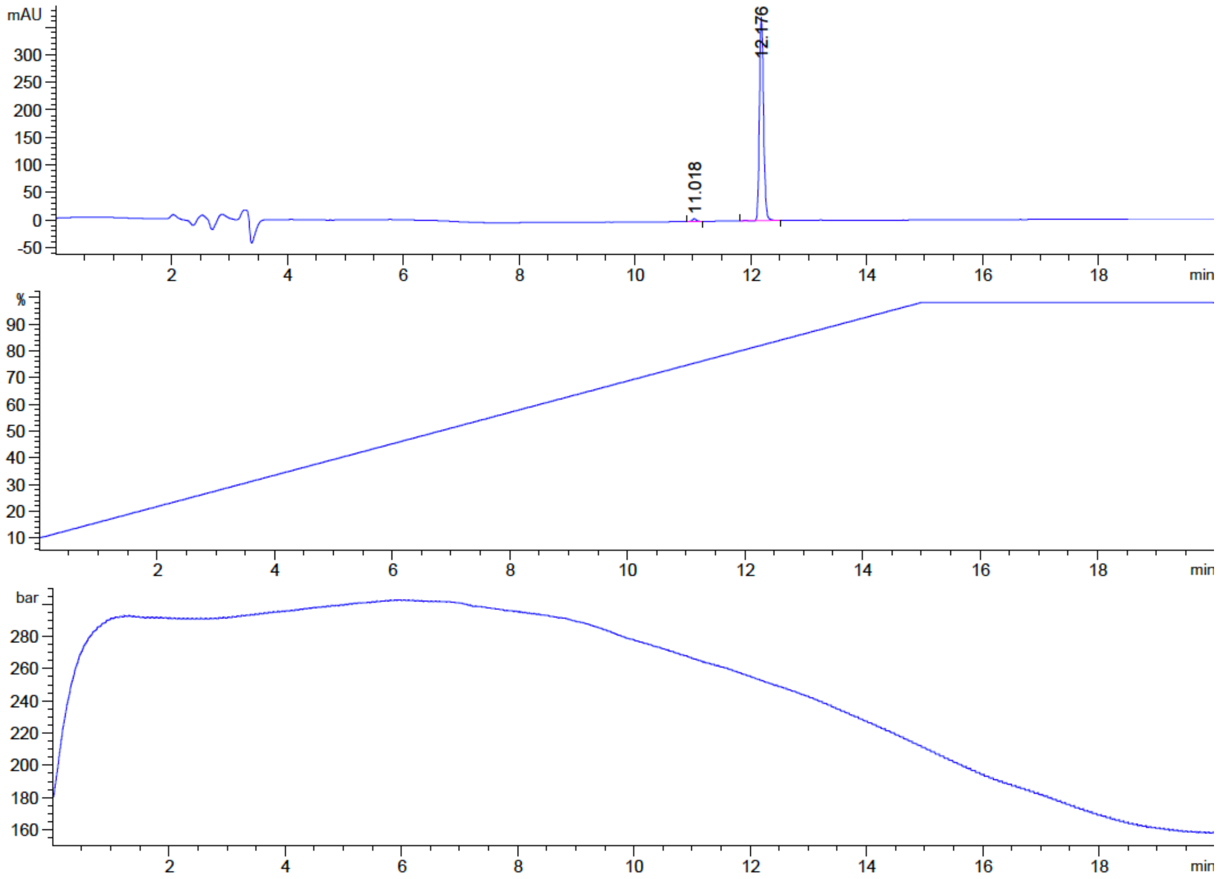

Signal 1: DAD1 A, Sig=297,4 Ref=off

Peak #	RetTime [min]	Type	Width [min]	Area [mAU*s]	Height [mAU]	Area %
1	11.018	BB	0.0767	21.72623	4.46688	1.1498
2	12.176	VB R	0.0787	1867.78650	370.98001	98.8502

Irradiation with λ = 365 nm for conversion from *trans* isomer to *cis* isomer.
Detection at 297 nm: t_R *cis* isomer = 8.511 min (93%), t_R *trans* isomer = 9.694 min (7%).

Figures of analytical HPLC trace, solvent gradient (acetonitrile/H_2O + 0.01% TFA), pressure profile and data analysis:

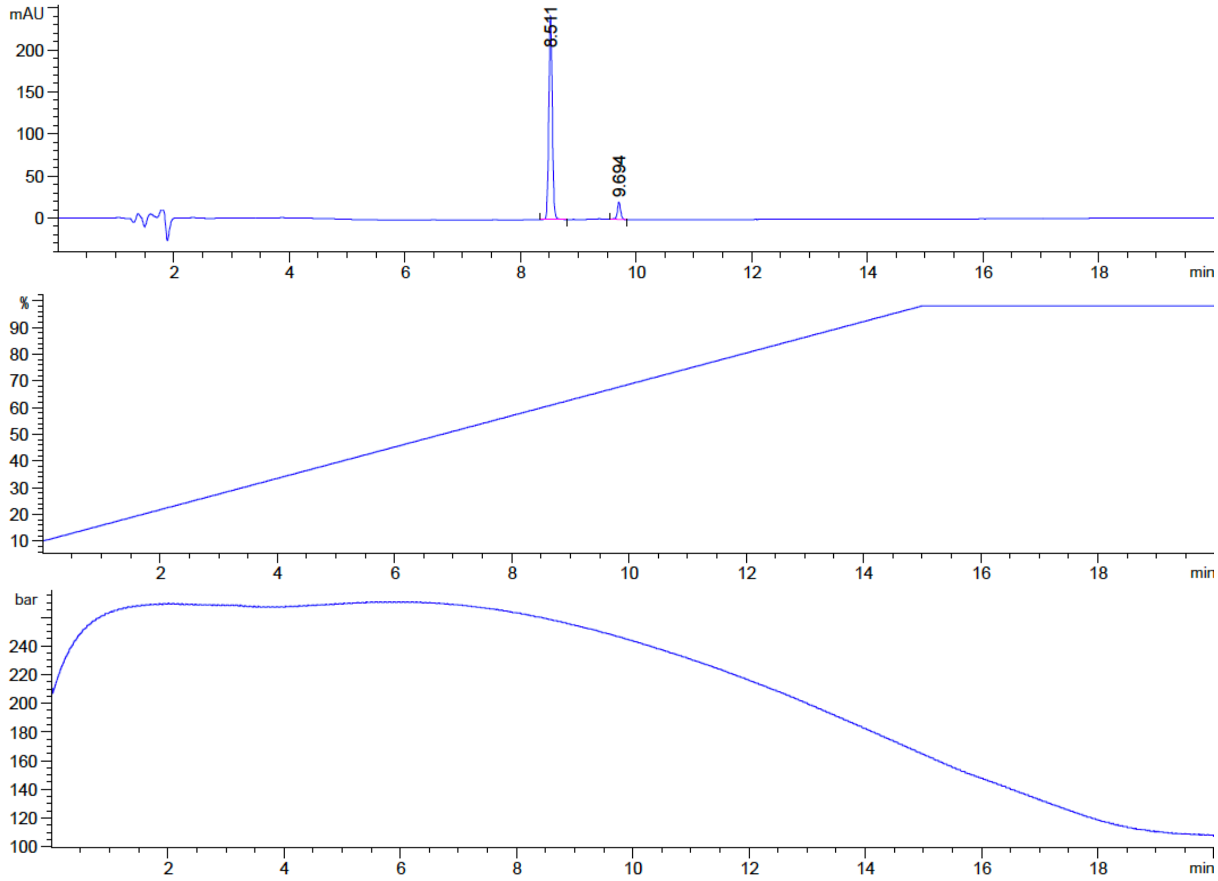

Signal 1: DAD1 A, Sig=297,4 Ref=off

Peak #	RetTime [min]	Type	Width [min]	Area [mAU*s]	Height [mAU]	Area %
1	8.511	BB	0.0652	1025.34265	243.28827	92.9756
2	9.694	BB	0.0592	77.46544	19.98244	7.0244

Compound 1 (50 µM solution in DMSO, injection volume 10 µL):

Irradiation with λ = 528 nm for conversion from *cis* isomer to *trans* isomer.
Detection at 297 nm: t_R *cis* isomer = 10.771 min (6%), t_R *trans* isomer = 11.946 min (94%).

Figures of analytical HPLC trace, solvent gradient (acetonitrile/H_2O + 0.01% TFA), pressure profile and data analysis:

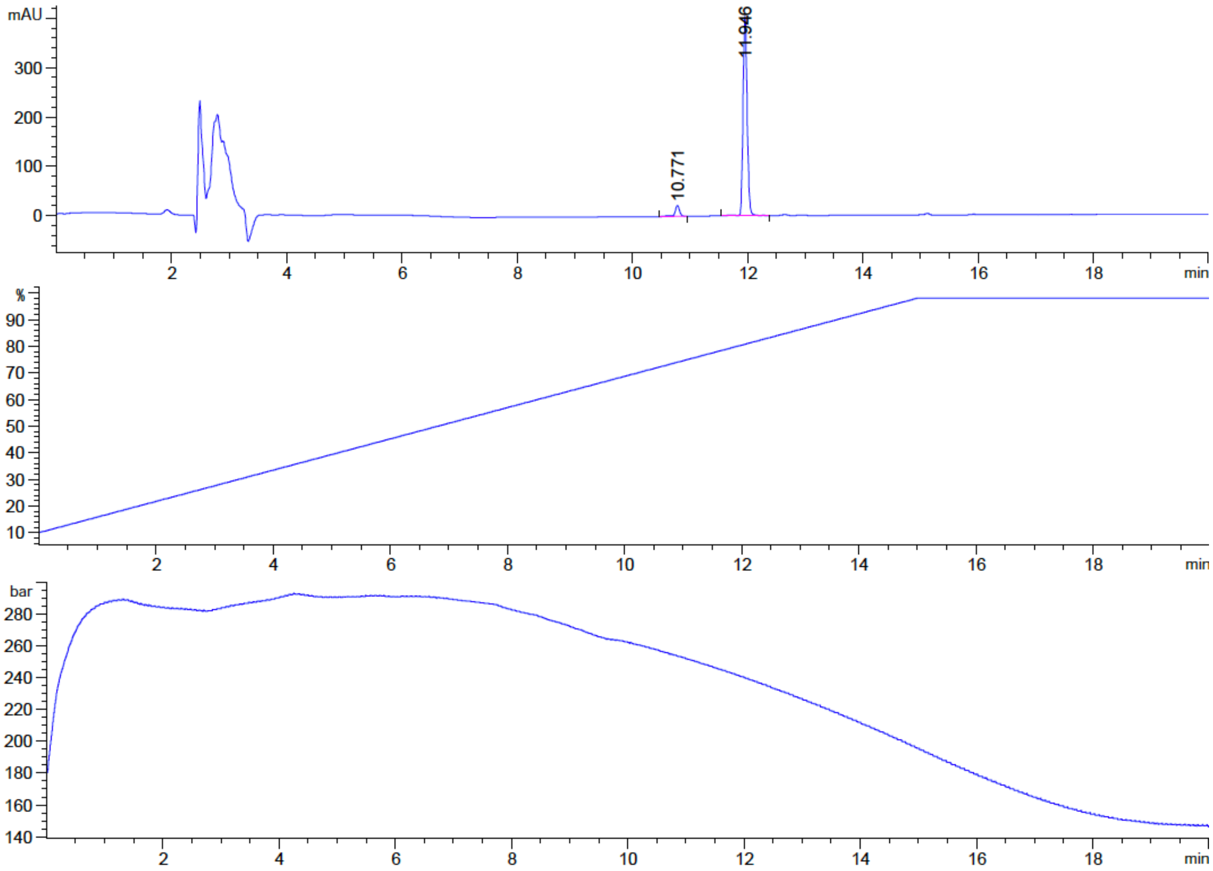

Signal 1: DAD1 A, Sig=297,4 Ref=off

Peak #	RetTime [min]	Type	Width [min]	Area [mAU*s]	Height [mAU]	Area %
1	10.771	VB R	0.0743	118.93340	21.96607	5.6250
2	11.946	VV R	0.0754	1995.42334	403.11578	94.3750

Irradiation with λ = 365 nm for conversion from *trans* isomer to *cis* isomer.
Detection at 297 nm: t_R *cis* isomer = 9.053 min (94%), t_R *trans* isomer = 10.230 min (6%).

Figures of analytical HPLC trace, solvent gradient (acetonitrile/H_2O + 0.01% TFA), pressure profile and data analysis:

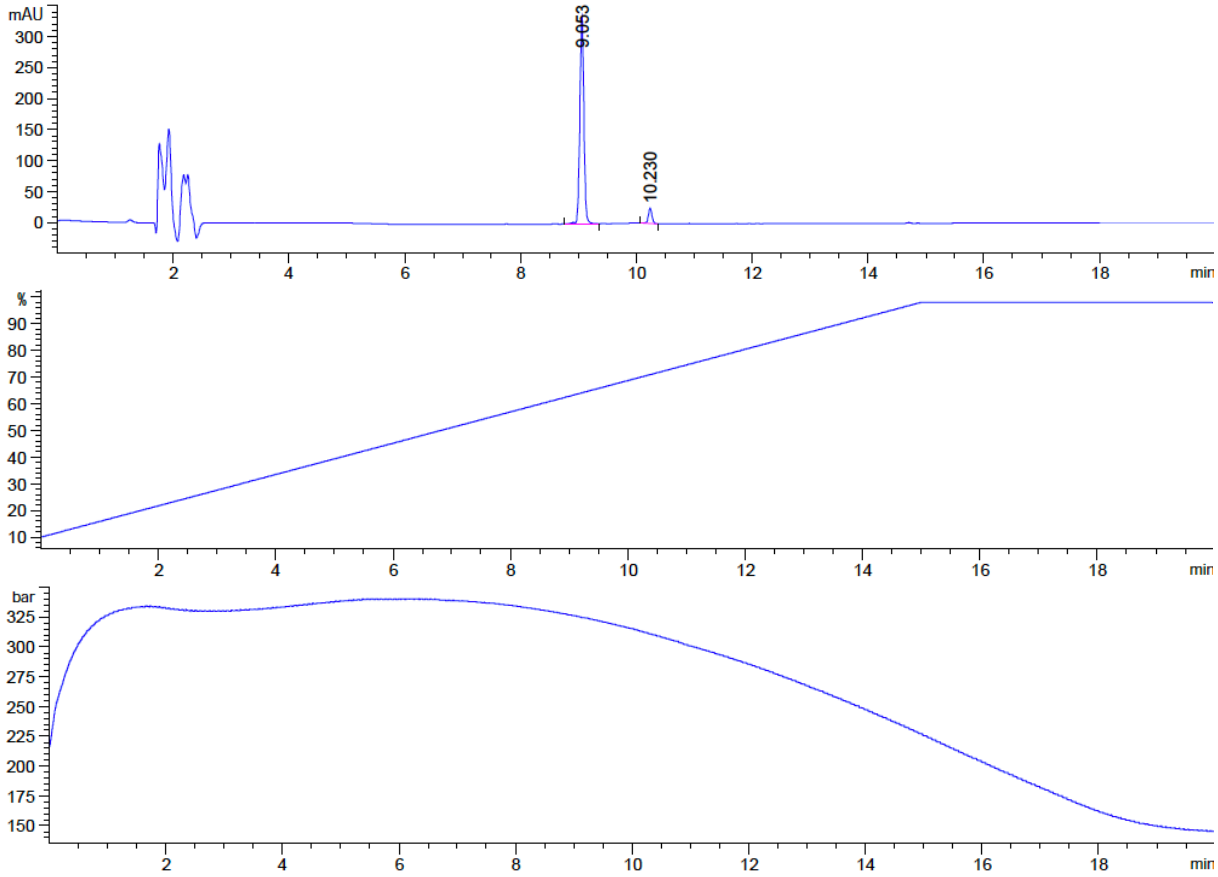

Signal 1: DAD1 A, Sig=297,4 Ref=off

Peak #	RetTime [min]	Type	Width [min]	Area [mAU*s]	Height [mAU]	Area %
1	9.053	VB R	0.0747	1643.58459	335.45016	93.8331
2	10.230	BB	0.0666	108.01983	24.92130	6.1669

Compound 3a (50 µM solution in TrisHCl Buffer + 0.5% DMSO, pH 7.5, injection volume 10 µL):

Irradiation with λ = 528 nm for conversion from *cis* isomer to *trans* isomer.
Detection at 290 nm: t_R *cis* isomer = 9.941 min (7%), t_R *trans* isomer = 11.099 min (93%).

Figures of analytical HPLC trace, solvent gradient (acetonitrile/H_2O + 0.01% TFA), pressure profile and data analysis:

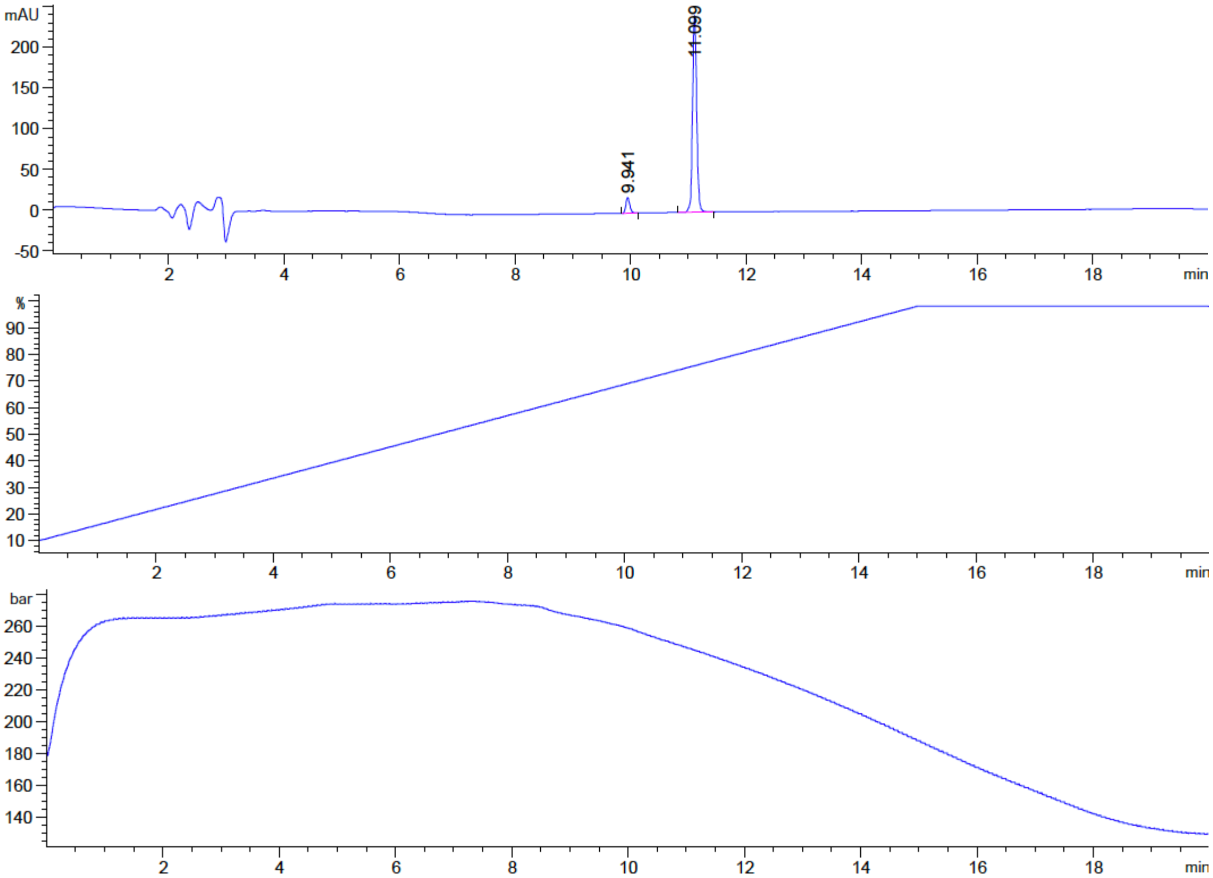

Signal 3: DAD1 C, Sig=290,4 Ref=off

Peak #	RetTime [min]	Type	Width [min]	Area [mAU*s]	Height [mAU]	Area %
1	9.941	BB	0.0730	91.82278	19.46816	7.1141
2	11.099	BB	0.0780	1198.88672	240.98196	92.8859

Irradiation with λ = 365 nm for conversion from *trans* isomer to *cis* isomer.
Detection at 290 nm: t_R *cis* isomer = 7.743 min (94%), t_R *trans* isomer = 8.977 min (6%).

Figures of analytical HPLC trace, solvent gradient (acetonitrile/H_2O + 0.01% TFA), pressure profile and data analysis:

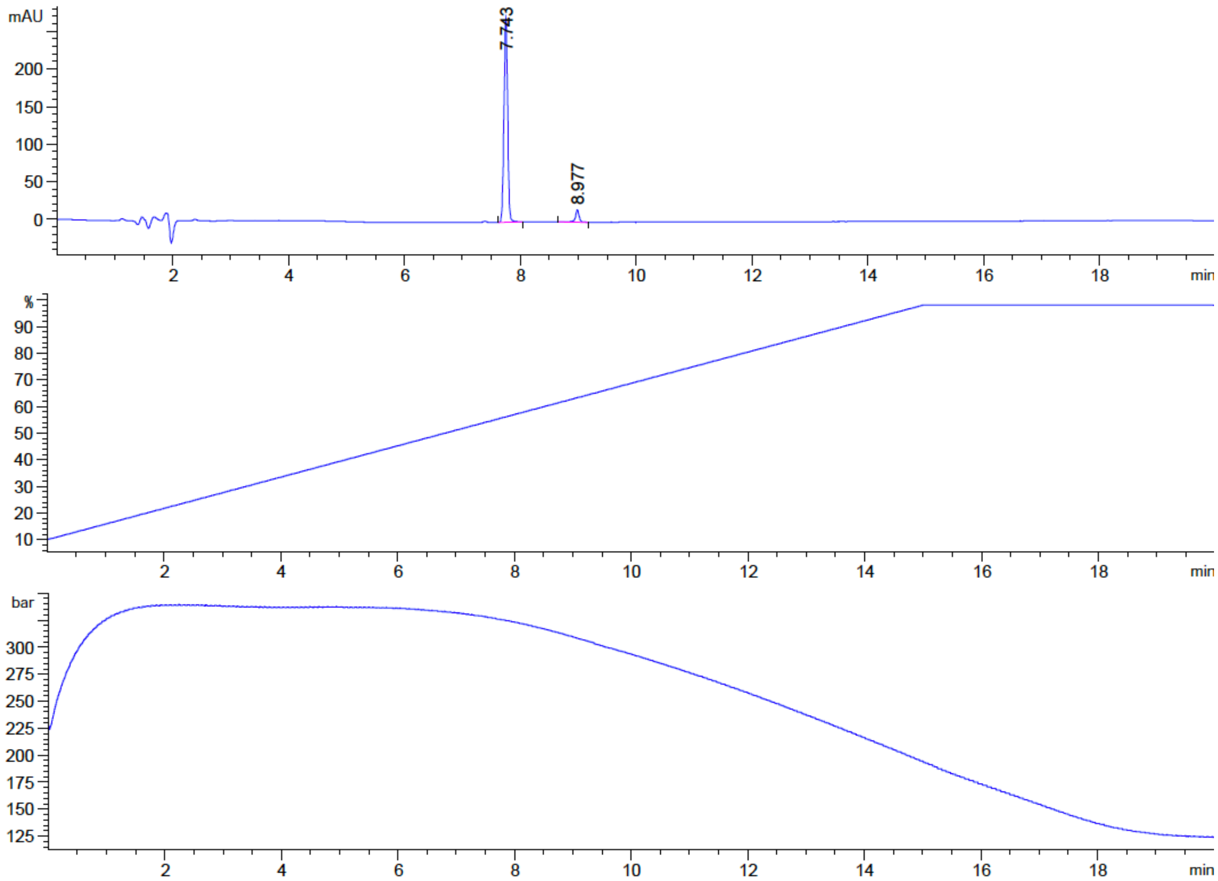

Signal 3: DAD1 C, Sig=290,4 Ref=off

Peak #	RetTime [min]	Type	Width [min]	Area [mAU*s]	Height [mAU]	Area %
1	7.743	BB	0.0669	1186.94019	271.91513	94.2678
2	8.977	BB	0.0653	72.17484	16.41975	5.7322

Compound 3a (50 µM solution in DMSO, injection volume 10 µL):

Irradiation with λ = 528 nm for conversion from *cis* isomer to *trans* isomer.
Detection at 300 nm: t_R *cis* isomer = 9.961 min (5%), t_R *trans* isomer = 11.140 min (95%).

Figures of analytical HPLC trace, solvent gradient (acetonitrile/H_2O + 0.01% TFA), pressure profile and data analysis:

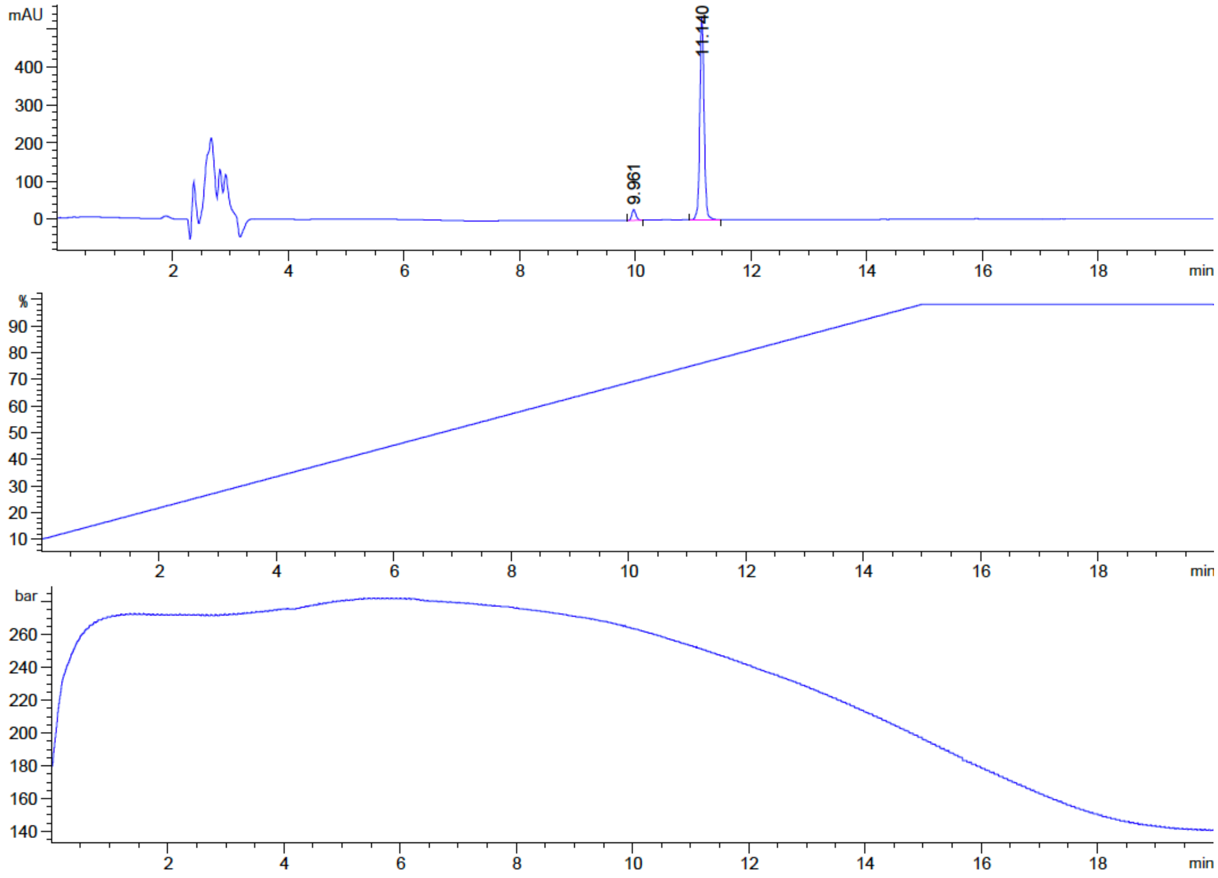

Signal 7: DAD1 G, Sig=300,4 Ref=off

Peak #	RetTime [min]	Type	Width [min]	Area [mAU*s]	Height [mAU]	Area %
1	9.961	BB	0.0763	135.44861	28.06516	4.7216
2	11.140	BB	0.0797	2733.22607	534.48822	95.2784

Irradiation with λ = 365 nm for conversion from *trans* isomer to *cis* isomer.
Detection at 300 nm: t_R *cis* isomer = 8.228 min (93%), t_R *trans* isomer = 9.432 min (7%).

Figures of analytical HPLC trace, solvent gradient (acetonitrile/H_2O + 0.01% TFA), pressure profile and data analysis:

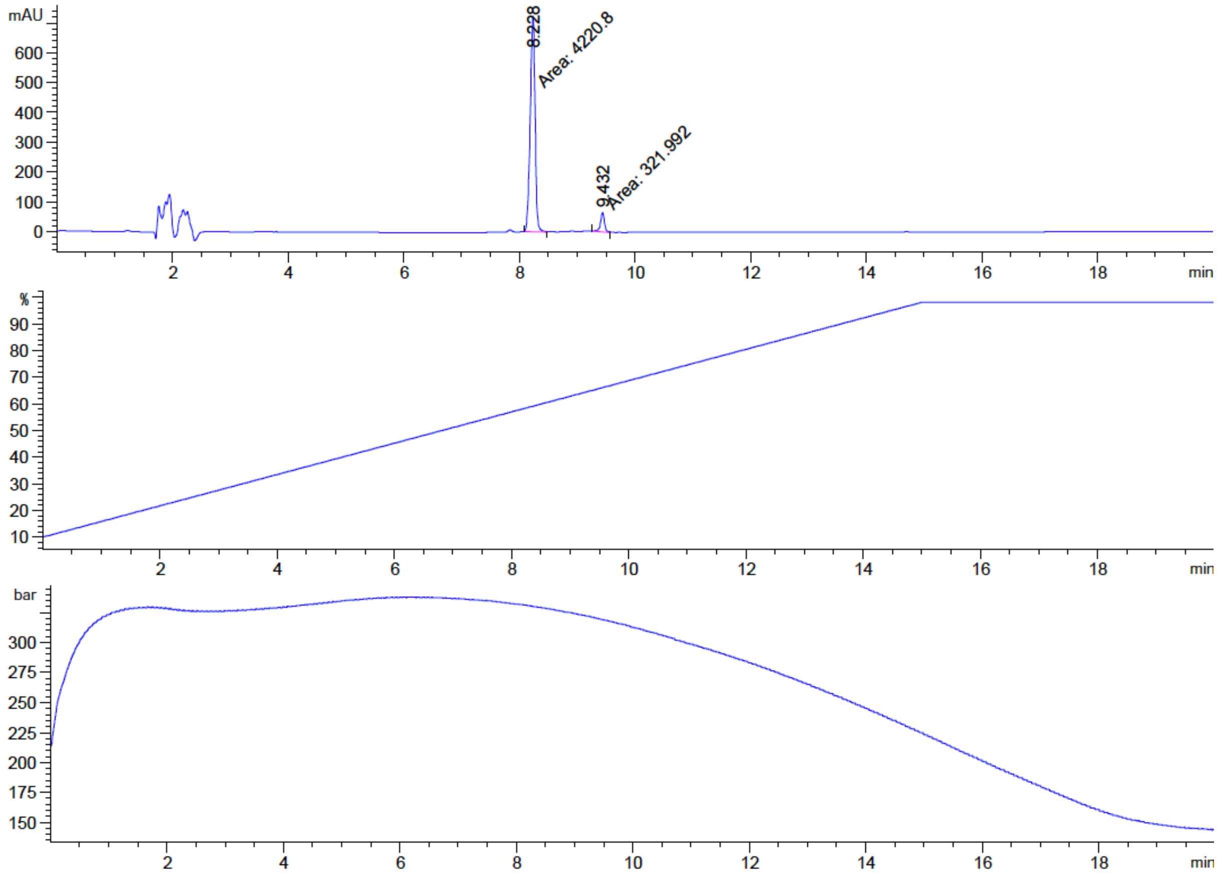


```
Signal 7: DAD1 G, Sig=300,4 Ref=off

Peak RetTime Type  Width     Area       Height     Area
  #    [min]        [min]    [mAU*s]    [mAU]        %
----|-------|----|-------|----------|----------|--------|
   1    8.228 MM    0.0969 4220.80273  725.84991  92.9120
   2    9.432 MM    0.0824  321.99194   65.15233   7.0880
```

Compound 3b (50 µM solution in TrisHCl Buffer + 0.5% DMSO, pH 7.5, injection volume 10 µL):

Irradiation with λ = 528 nm for conversion from *cis* isomer to *trans* isomer.
Detection at 297 nm: t_R *cis* isomer = 7.821 min (7%), t_R *trans* isomer = 8.823 min (93%).

Figures of analytical HPLC trace, solvent gradient (acetonitrile/H_2O + 0.01% TFA), pressure profile and data analysis:

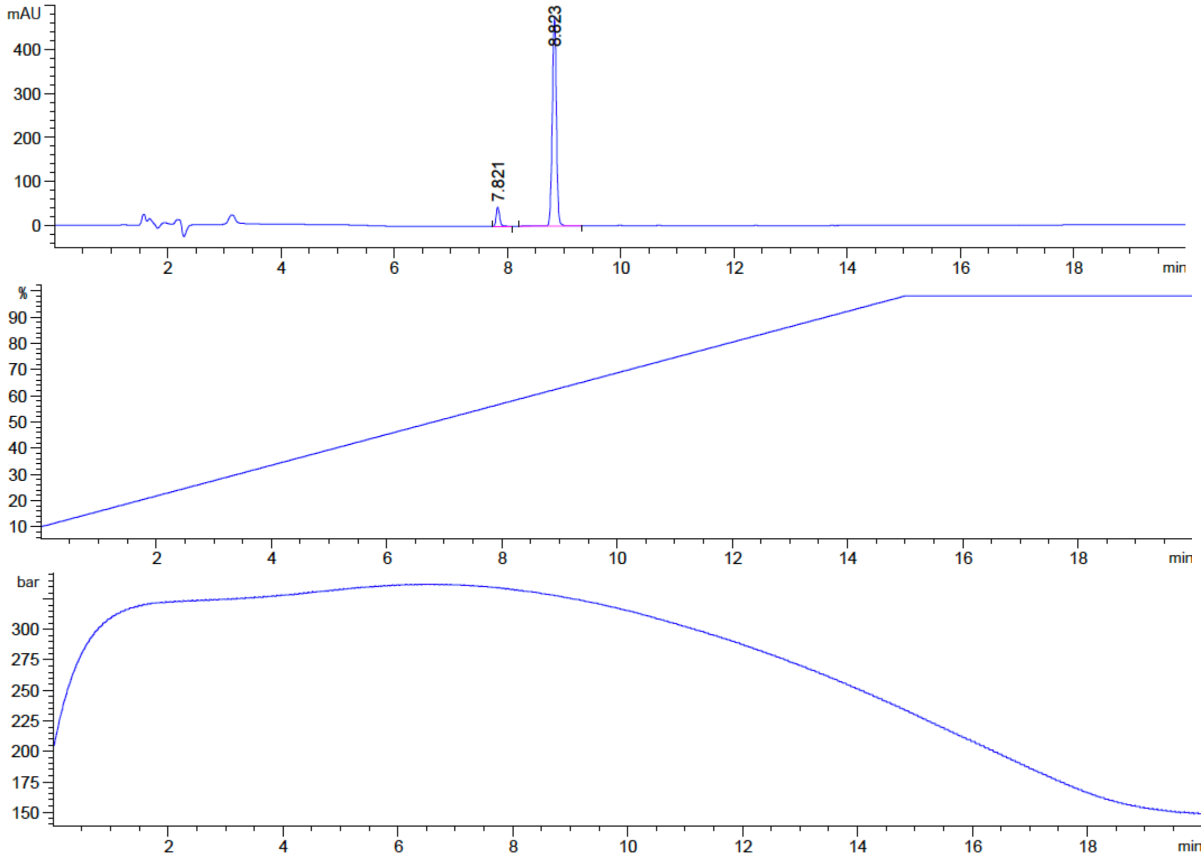

Signal 3: DAD1 C, Sig=297,4 Ref=off

Peak #	RetTime [min]	Type	Width [min]	Area [mAU*s]	Height [mAU]	Area %
1	7.821	BB	0.0634	179.10533	44.08815	6.8857
2	8.823	VV R	0.0768	2422.00635	479.68219	93.1143

Irradiation with λ = 365 nm for conversion from *trans* isomer to *cis* isomer.
Detection at 297 nm: t_R *cis* isomer = 7.897 min (93%), t_R *trans* isomer = 8.768 min (7%).

Figures of analytical HPLC trace, solvent gradient (acetonitrile/H_2O + 0.01% TFA), pressure profile and data analysis:

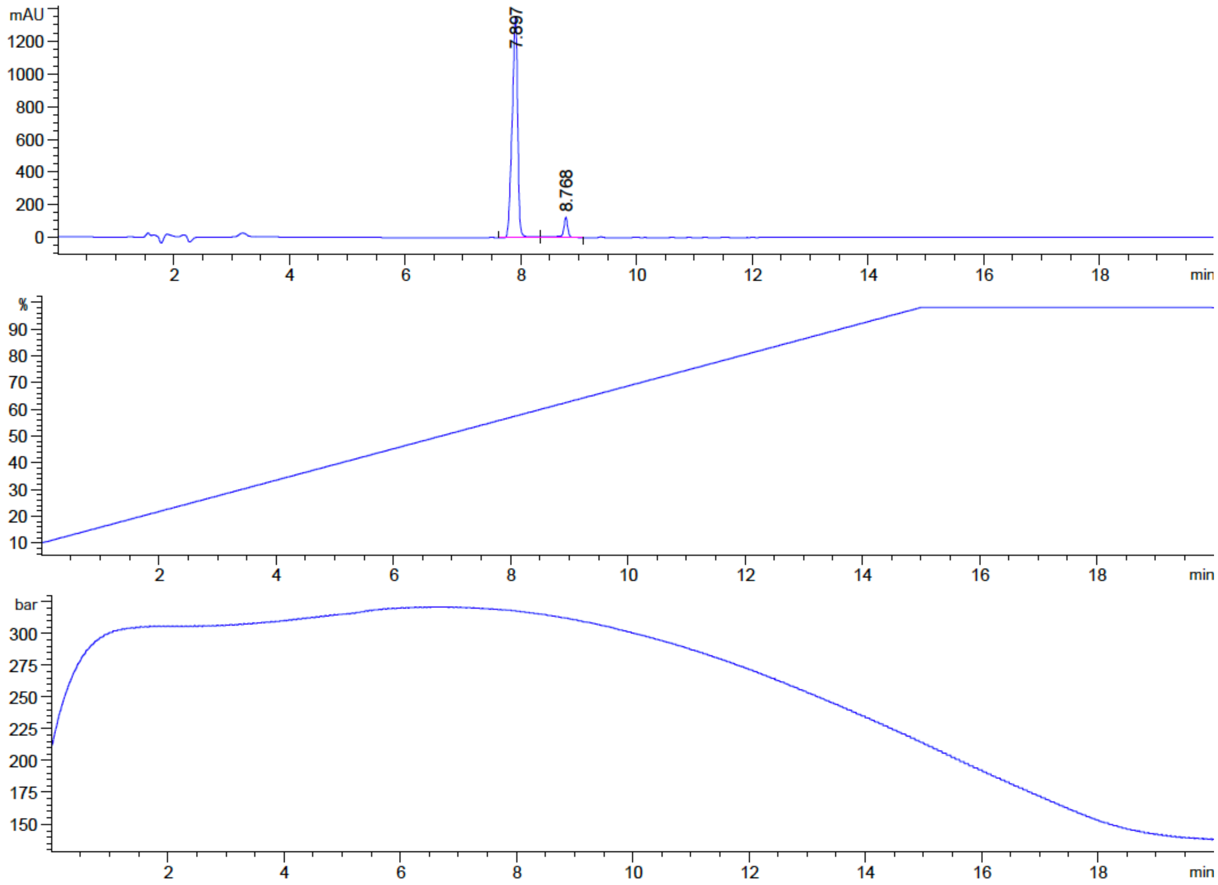

Signal 3: DAD1 C, Sig=297,4 Ref=off

Peak #	RetTime [min]	Type	Width [min]	Area [mAU*s]	Height [mAU]	Area %
1	7.897	BV R	0.0970	8982.23438	1351.64453	92.7435
2	8.768	VV R	0.0833	702.79816	124.06384	7.2565

Compound 3b (50 µM solution in DMSO, injection volume 10 µL):

Irradiation with λ = 528 nm for conversion from *cis* isomer to *trans* isomer.
Detection at 301 nm: t_R *cis* isomer = 7.822 min (4%), t_R *trans* isomer = 8.845 min (96%).

Figures of analytical HPLC trace, solvent gradient (acetonitrile/H_2O + 0.01% TFA), pressure profile and data analysis:

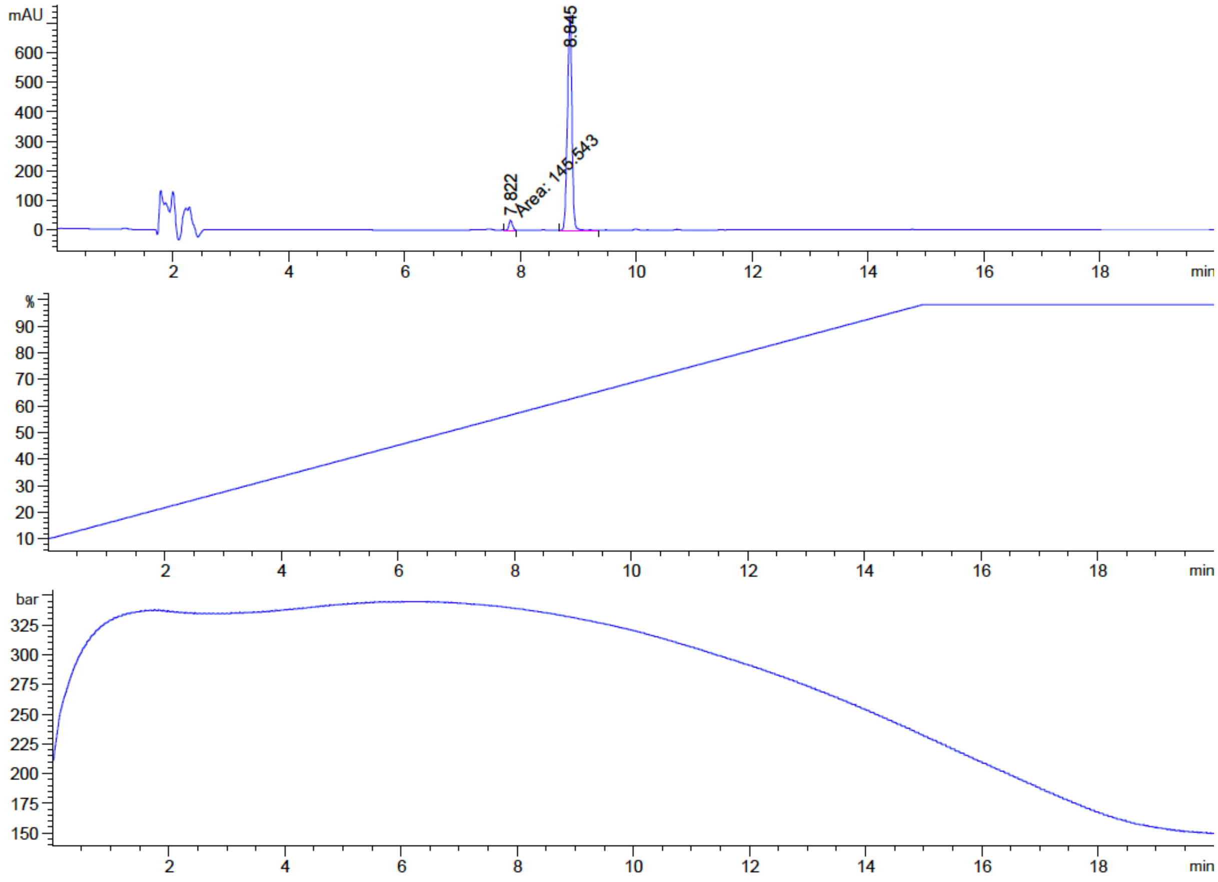

Signal 7: DAD1 G, Sig=301,4 Ref=off

Peak #	RetTime [min]	Type	Width [min]	Area [mAU*s]	Height [mAU]	Area %
1	7.822	MM	0.0720	145.54337	33.69057	3.5492
2	8.845	BV R	0.0834	3955.23389	728.08710	96.4508

Irradiation with λ = 365 nm for conversion from *trans* isomer to *cis* isomer.
Detection at 301 nm: t_R *cis* isomer = 7.830 min (93%), t_R *trans* isomer = 8.753 min (7%).

Figures of analytical HPLC trace, solvent gradient (acetonitrile/H_2O + 0.01% TFA), pressure profile and data analysis:

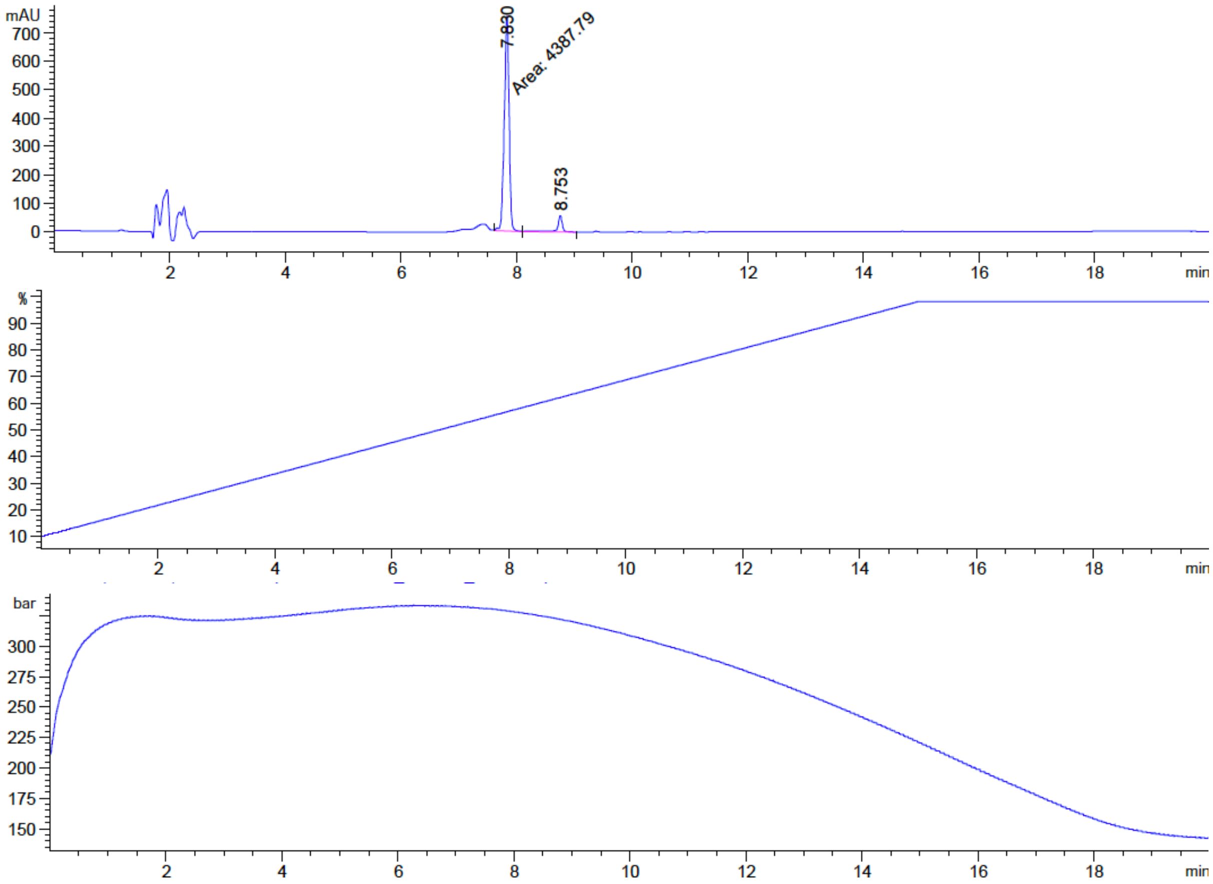

Signal 7: DAD1 G, Sig=301,4 Ref=off

Peak #	RetTime [min]	Type	Width [min]	Area [mAU*s]	Height [mAU]	Area %
1	7.830	MM	0.0964	4387.78564	758.81024	93.0113
2	8.753	VV R	0.0800	329.69113	57.61179	6.9887

Compound 3c (20 µM solution in TrisHCl Buffer + 0.2% DMSO, pH 7.5, injection volume 10 µL):

Irradiation with λ = 528 nm for conversion from *cis* isomer to *trans* isomer.
Detection at 291 nm: t_R *cis* isomer = 9.630 min (7%), t_R *trans* isomer = 10.909 min (93%).

Figures of analytical HPLC trace, solvent gradient (acetonitrile/H_2O + 0.01% TFA), pressure profile and data analysis:

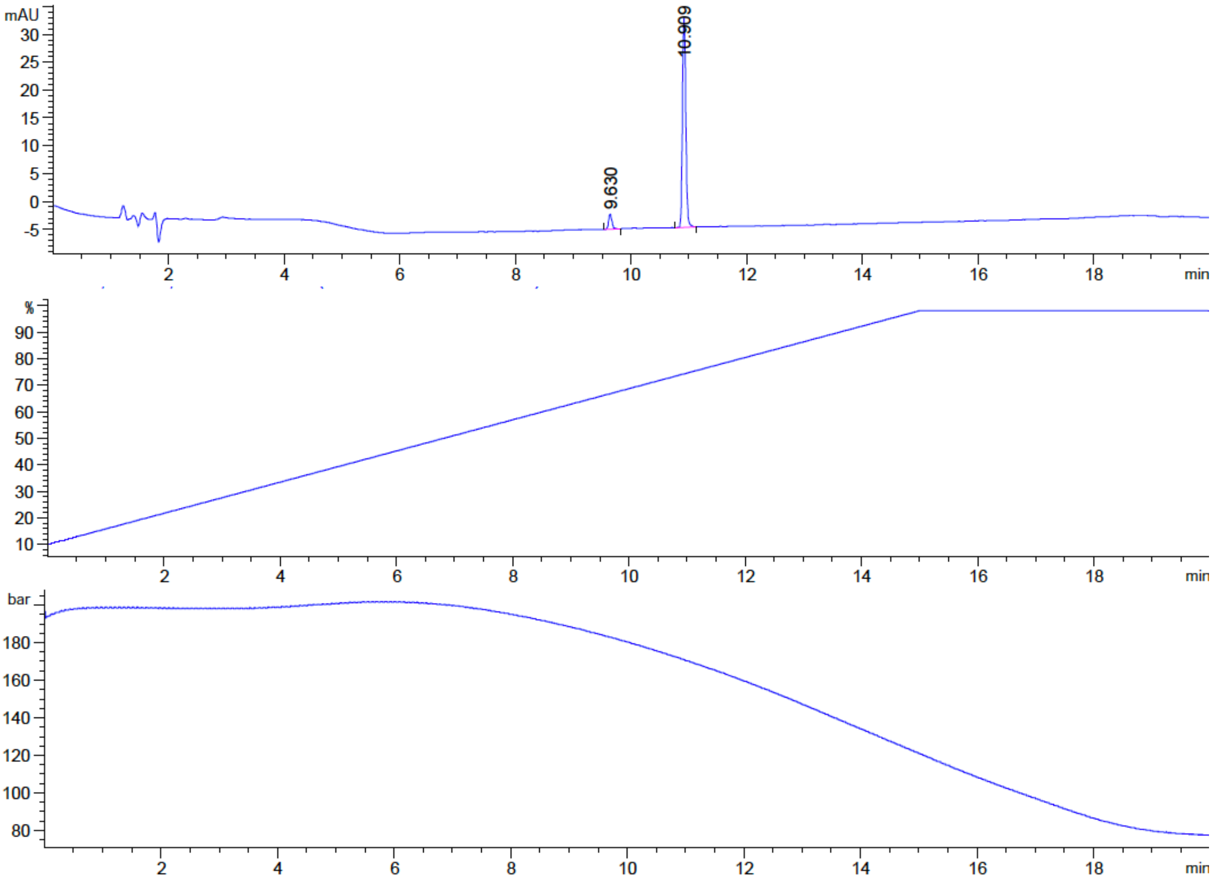

Signal 3: DAD1 C, Sig=291,4 Ref=off

Peak #	RetTime [min]	Type	Width [min]	Area [mAU*s]	Height [mAU]	Area %
1	9.630	BB	0.0631	10.74787	2.66116	6.5235
2	10.909	BB	0.0633	154.00816	37.97816	93.4765

Irradiation with λ = 365 nm for conversion from *trans* isomer to *cis* isomer.
Detection at 291 nm: t_R *cis* isomer = 9.632 min (91%), t_R *trans* isomer = 10.901 min (9%).

Figures of analytical HPLC trace, solvent gradient (acetonitrile/H_2O + 0.01% TFA), pressure profile and data analysis:

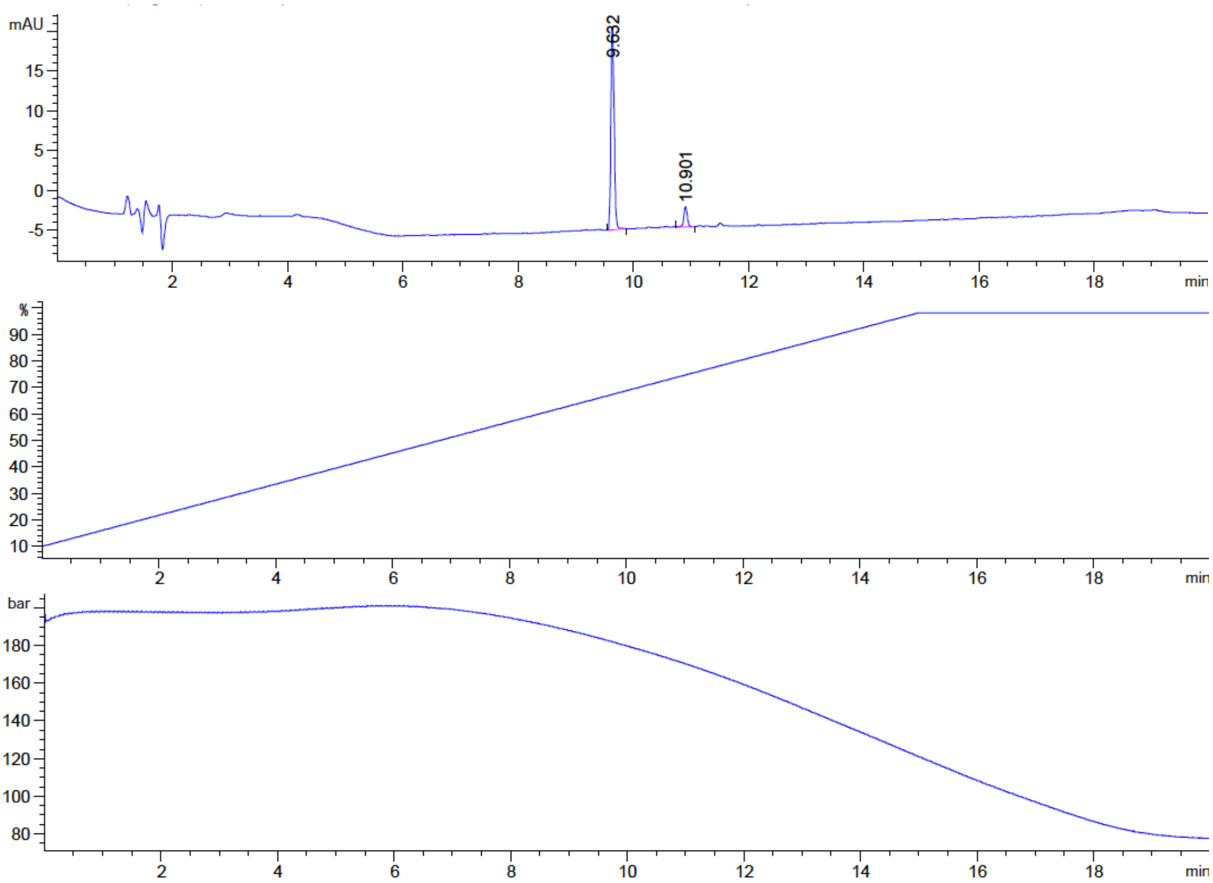

Signal 3: DAD1 C, Sig=291,4 Ref=off

Peak #	RetTime [min]	Type	Width [min]	Area [mAU*s]	Height [mAU]	Area %
1	9.632	BB	0.0623	101.88867	25.64798	90.6958
2	10.901	BB	0.0647	10.45243	2.50319	9.3042

Compound 3c (50 µM solution in DMSO, injection volume 10 µL):

Irradiation with λ = 528 nm for conversion from *cis* isomer to *trans* isomer.
Detection at 303 nm: t_R *cis* isomer = 10.049 min (6%), t_R *trans* isomer = 11.236 min (94%).

Figures of analytical HPLC trace, solvent gradient (acetonitrile/H_2O + 0.01% TFA), pressure profile and data analysis:

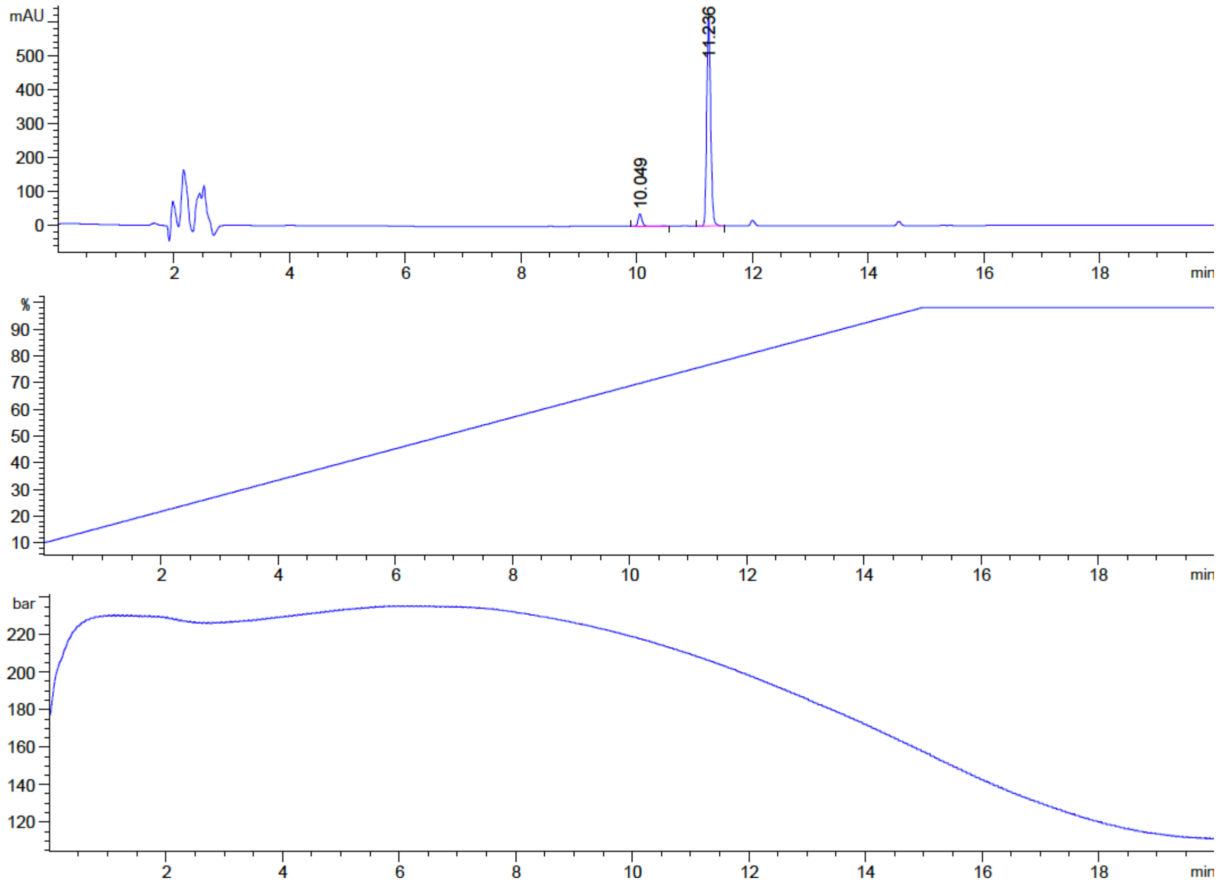

Signal 7: DAD1 G, Sig=303,4 Ref=off

Peak #	RetTime [min]	Type	Width [min]	Area [mAU*s]	Height [mAU]	Area %
1	10.049	VV R	0.0719	172.46210	35.95505	5.5812
2	11.236	BB	0.0734	2917.61401	614.87427	94.4188

Irradiation with λ = 365 nm for conversion from *trans* isomer to *cis* isomer.
Detection at 303 nm: t_R *cis* isomer = 10.288 min (96%), t_R *trans* isomer = 11.487 min (4%).

Figures of analytical HPLC trace, solvent gradient (acetonitrile/H_2O + 0.01% TFA), pressure profile and data analysis:

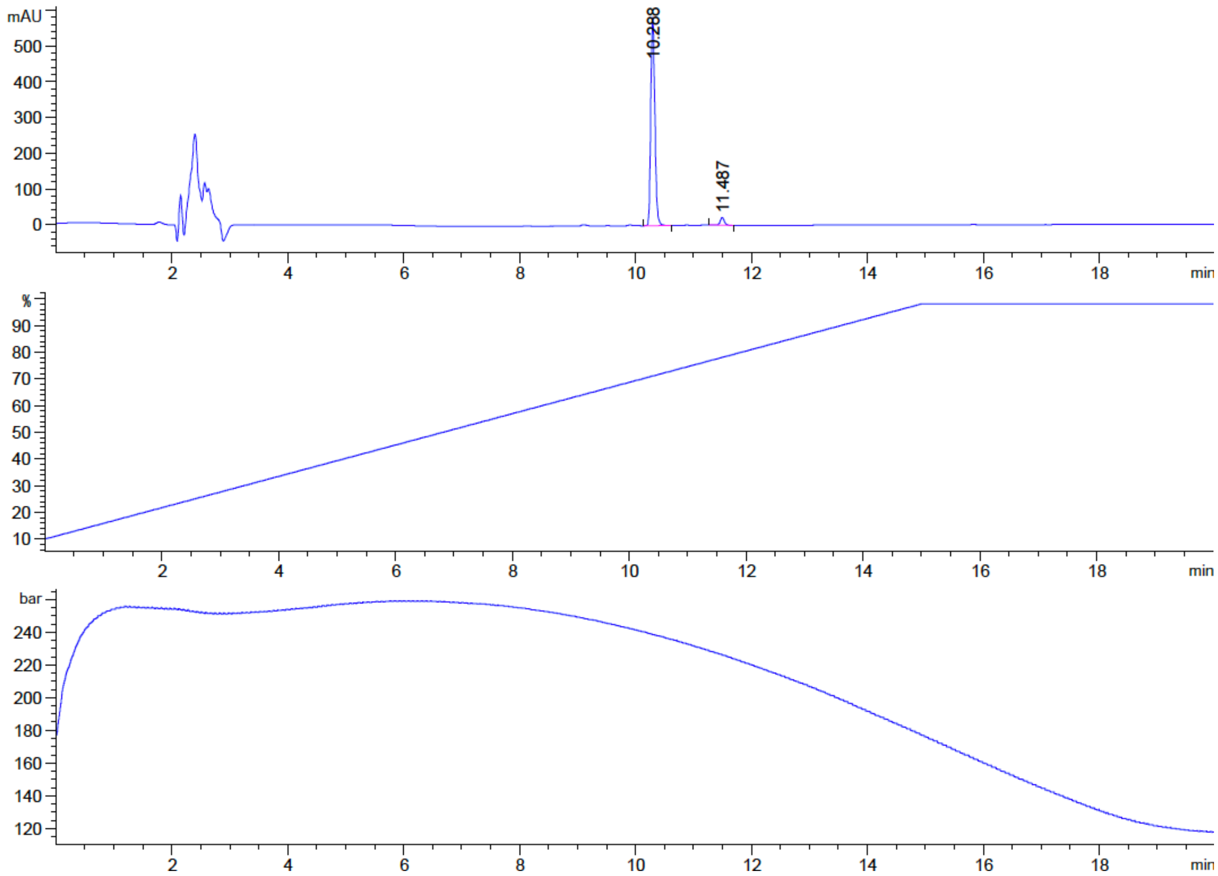

Signal 7: DAD1 G, Sig=303,4 Ref=off

Peak #	RetTime [min]	Type	Width [min]	Area [mAU*s]	Height [mAU]	Area %
1	10.288	VB	0.0745	2821.02271	582.58197	96.3605
2	11.487	BB	0.0785	106.55003	21.23673	3.6395

3.4. Degradation of Compound 4 Upon Exposure to UV Irradiation

3.4.1. UV/Vis Spectroscopy to Monitor Effects of Light Exposure.

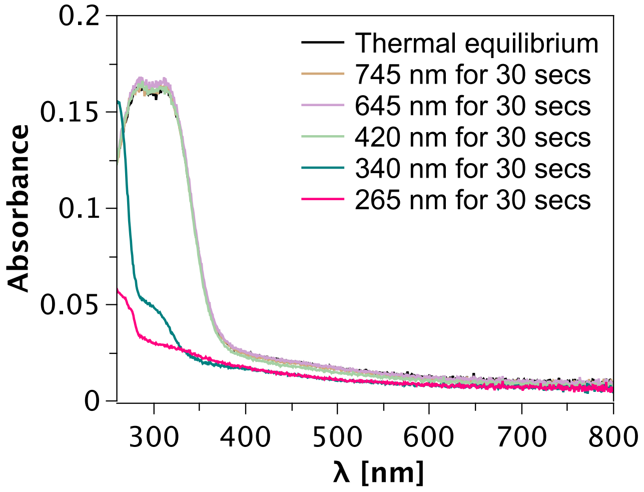

SI Figure 6. UV/Vis absorbance spectra of compound **4** upon light exposure with various wavelengths. Irradiation with wavelengths of 745, 645, 528 and 420 nm did not significantly change UV/Vis spectral absorbance, when compared to thermal equilibrium. However, irradiation of 340 and 265 nm significantly diminished absorbance. Compound **4** (20 µM) was evaluated in buffer solution (TrisHCl Buffer, pH 7.5) + 0.5% DMSO at 25 ºC.

3.4.2. Analytical HPLC to Monitor Effects of Light Exposure.

Compound **4** (50 µM solution in TrisHCl buffer, pH 7.4 + 0.2% DMSO) in dark conditions:

Detection at 280 nm: tR = 19.952 min.

Figures of analytical HPLC trace, solvent gradient (acetonitrile/H_2O + 0.01% TFA), pressure profile and data analysis:

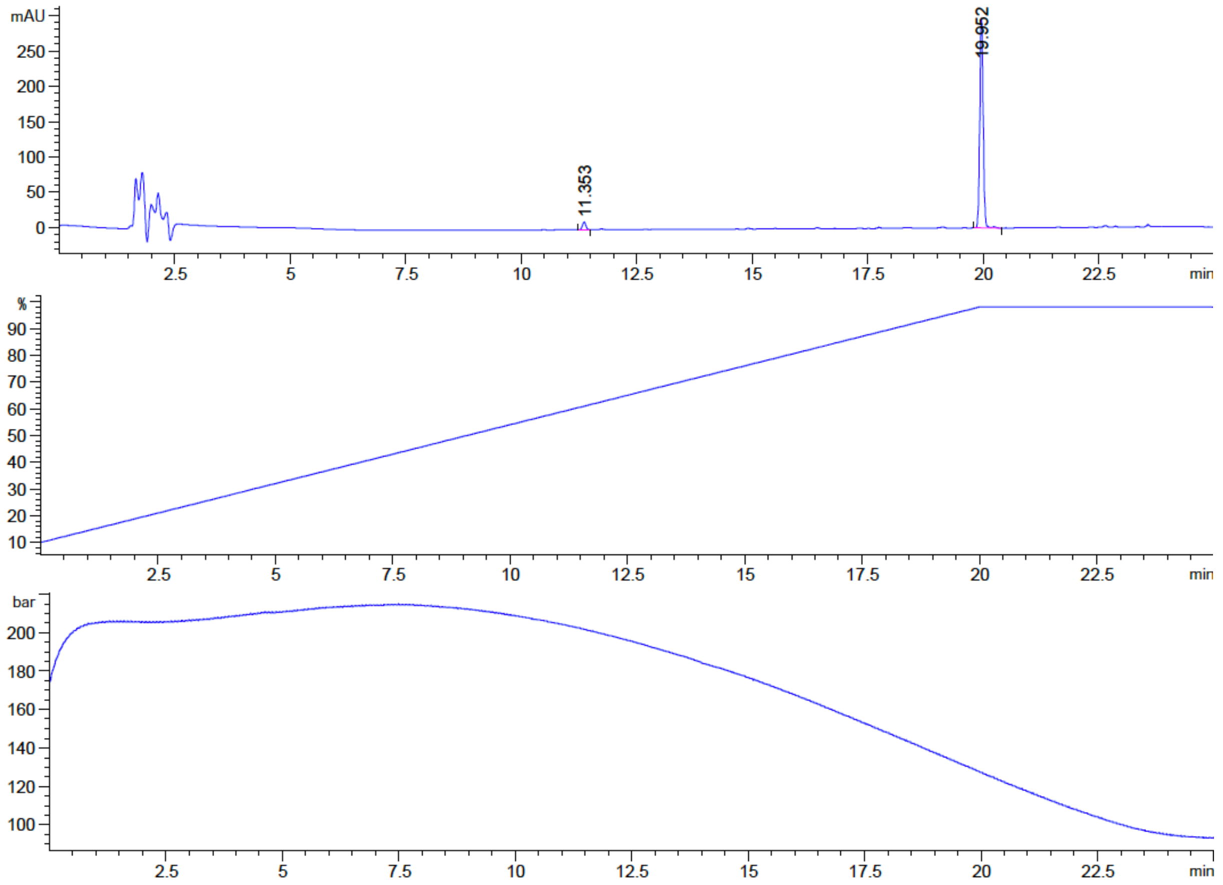

Signal 3: DAD1 C, Sig=280,4 Ref=off

Peak #	RetTime [min]	Type	Width [min]	Area [mAU*s]	Height [mAU]	Area %
1	11.353	BB	0.0803	57.70715	11.15608	3.6514
2	19.952	BV R	0.0795	1522.69250	297.17880	96.3486

The same sample of compound **4** after irradiation with λ = 285 nm for 20 seconds, with the same HPLC instrument, set-up and conditions.

Detection at 280 nm: tR = 21.157 min. A significant reduction in absorbance height (mAU) can be observed.

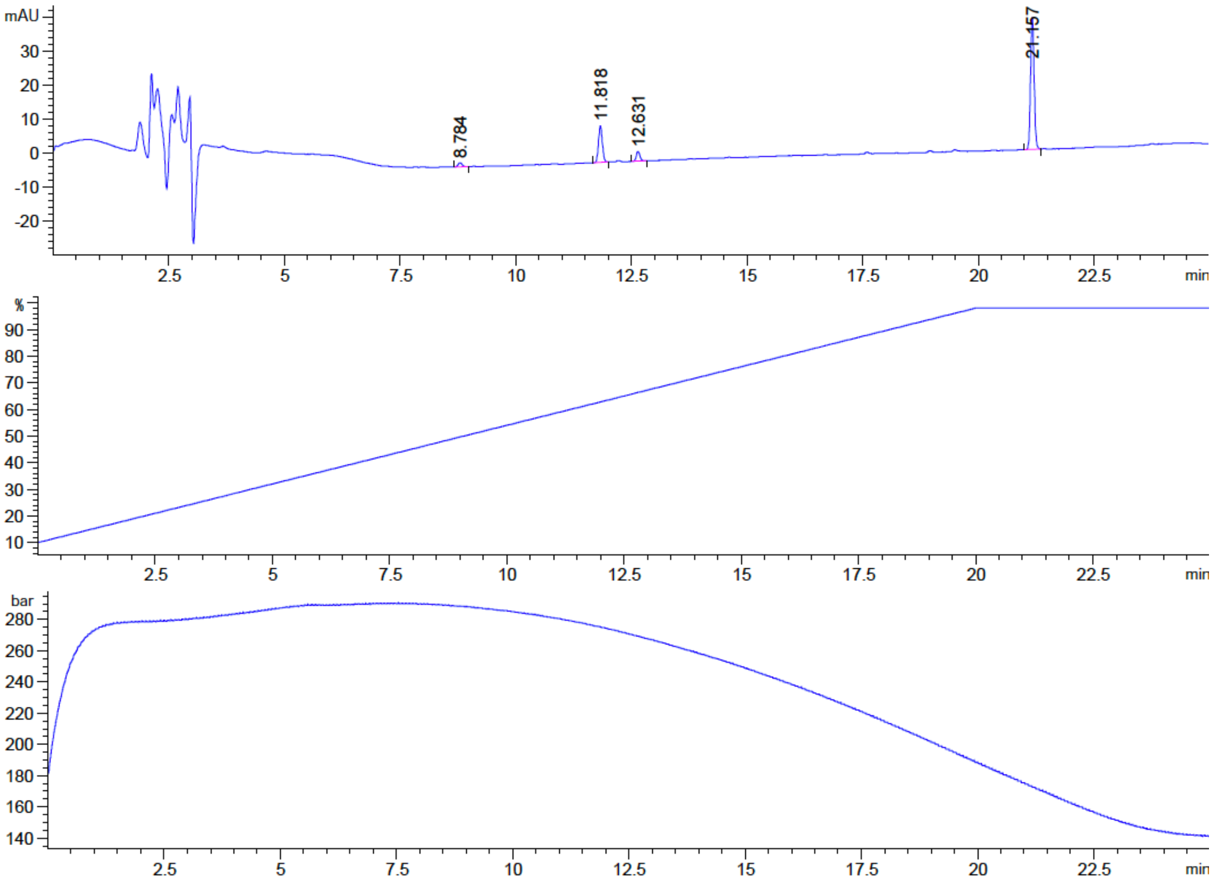

Signal 3: DAD1 C, Sig=280,4 Ref=off

Peak #	RetTime [min]	Type	Width [min]	Area [mAU*s]	Height [mAU]	Area %
1	8.784	BB	0.0960	7.59672	1.23146	2.5574
2	11.818	BB	0.0913	62.59029	10.85392	21.0706
3	12.631	BB	0.0855	16.27894	2.89927	5.4802
4	21.157	BB	0.0828	210.58388	39.12384	70.8918

4. Supplementary Biochemical Information

4.1. General Information

Radioligand Binding

Binding affinities towards the human opioid receptor μOR were determined as described previously.[7] In brief, membranes were prepared from HEK293T cells transiently transfected with the cDNA for μOR (gift from the Ernest Gallo Clinic and Research Center, UCSF, CA) and incubated with the radioligand [3H]diprenorphine (specific activity 31 Ci/mmol; PerkinElmer, Rodgau, Germany) at concentrations of 0.2 to 0.3 nM. Homogenates expressing μOR with a Bmax of 2200 ± 360 fmol/mg protein and a KD of 0.089 ± 0.01 nM were incubated at an amount of protein of 3-10 μg/well with radioligand and varying concentrations of test compound (in the range of 10 pM - 100 μM) for 60 min in binding buffer (50 mM TRIS, pH 7.4) and filtered on glass fiber mats presoaked with 0.3% PEI solution. Trapped radioactivity was measured with a microplate reader (Microbeta Trilux, Perkin Elmer) by scintillation counting. To measure the photoswitchable compounds 200-300 μL of a working solution was irradiated with a single LED at 528 nm for 180 sec to switch to the *trans*-isomer and at 365 nm for 20 sec to obtain the *cis*-isomer. For switching trans-PF2 irradiation was done at 420 nm for 120 sec. Unspecific binding was determined in the presence of 10 μM of naloxone. Protein concentration was determined employing the method of Lowry with bovine serum albumin as standard.[8]

The resulting competition curves of the receptor binding experiments were analyzed by nonlinear regression using the algorithms in PRISM 9.0 (GraphPad Software, San Diego, USA). The data were initially fit using a sigmoid model to provide IC_{50} values which were subsequently transformed to Ki values according to the equation of Cheng and Prusoff.[9]

IP Accumulation Assay for Receptor Activation

The determination of receptor mediated G-protein signalling by μOR activation was performed applying an IP accumulation assay (IP-One HTRF®, PerkinElkmer-Cisbio, Rodgau, Germany) according to the manufacturer's protocol and in analogy to previously described protocols.[10] In brief, HEK 293T cells were co-transfected with the cDNA for μOR and the hybrid G-protein Gαqi5HA (Gαq protein with the last five amino acids at the C-terminus replaced by the corresponding sequence of Gαi (gift from The J. David Gladstone Institutes, San Francisco, CA), respectively and transferred into 384 well micro plates. Cells were incubated with test compound for 120 min and accumulation of second messenger was stopped by adding detection reagents (IP1-d2 conjugate and Anti-IP1cryptate TB conjugate). After 60 min, TR-FRET was measured with a Clariostar plate reader. FRET emission was measured at 620 nm and 665 nm, the corresponding ratio (emission at 665 nm/emission at 620 nm) was calculated and normalized to vehicle (0%) and the maximum effect of the reference DAMGO (100%). Each single experiment, performed in duplicate, was analyzed applying the algorithms for four parameter non-linear regression implemented in Prism 9.0 to get dose-response curves representing EC_{50} and E_{max} values and was repeated to get 3 to 11 independent values.

Photoisomerization experiments were performed by measuring IP accumulation as described above. In detail, 10,000 cells transiently co-transfected with μOR and Gαqi5HA were seeded in each well of a 96 well microplate (Greiner Bio-One, Frickenhausen, Germany). On the day of experiment, medium was removed by stimulation buffer supplemented with 30 μM of *trans/cis*-**3c** or 1 μM of DAMGO as a reference. Incubation was initiated by irradiation at 365 nm (for 20 sec) or 528 nm (for 180 sec) with LEDs directly placed upon the wells. As a reference, buffer and DAMGO were irradiated in parallel to the photoswitch ligand **3c** for each irradiation condition. The second irradiation step was performed similarly. Accumulation of IP was determined by TR-FRET measurement and normalized to the effect of buffer (0%) and DAMGO (100%), which resulted after an incubation of 120 min. Each experiment was performed in quadruplicate and was repeated to get 6 to 11 individual values.

4.2. Supplementary Figures and Tables

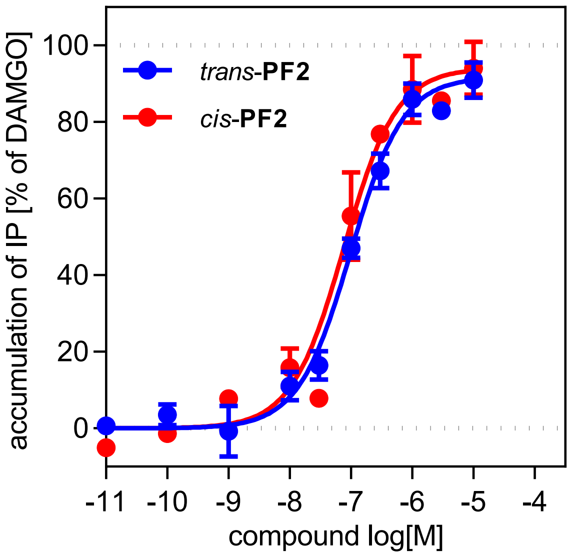

SI Figure 7. Activation of the µOR by *trans*-**PF2** and *cis*-**PF2**, measured by applying the IP-One® accumulation assay in HEK293T cells transiently co-transfected with µOR and the hybrid G-protein $G\alpha_{qi5HA}$. Both *trans*-**PF2** and *cis*-**PF2** show full agonist properties with E_{max} values of 93% and 95%, respectively, and similar EC_{50} potencies of 96 nM and 85 nM, respectively. Graphs show mean curves (± S.E.M.) of 3-4 single experiments, each performed in duplicate.

SI Table 1: Photoisomerization of *trans*/*cis*-**3c** during cell incubation, indicated by µOR-stimulated IP accumulation.

	**IP accumulation** [a]	
	E_{max} [% ± S.E.M.] [b]	(n) [c]
**A) *trans*-3c**		
**120 min**	76 ± 1	11
**30 min**	25 ± 5	6
**B) *cis*-3c**		
**120 min**	26 ± 2	11
**30 min**	10 ± 2	6
**C) *switching***		
***trans*- 30 min / *cis*- 90 min**	38 ± 4	6
***cis*- 30 min / *trans*- 90 min**	71 ± 2	6

[a] IP accumulation was determined by applying the IP-One® assay (Cisbio/PerkinElmer) with HEK293T cells transiently co-transfected with the human µOR and the hybrid G-protein $G\alpha_{qi5HA}$. [b] Mean EC_{50} value [% ± S.E.M.] normalized to the full effect of DAMGO after an incubation time of 120 min. [c] Number of individual experiments, each performed in duplicate.

5. ^{1}H and ^{13}C NMR Spectra

^{1}H spectrum of **6** (400 MHz, $CDCl_3$):

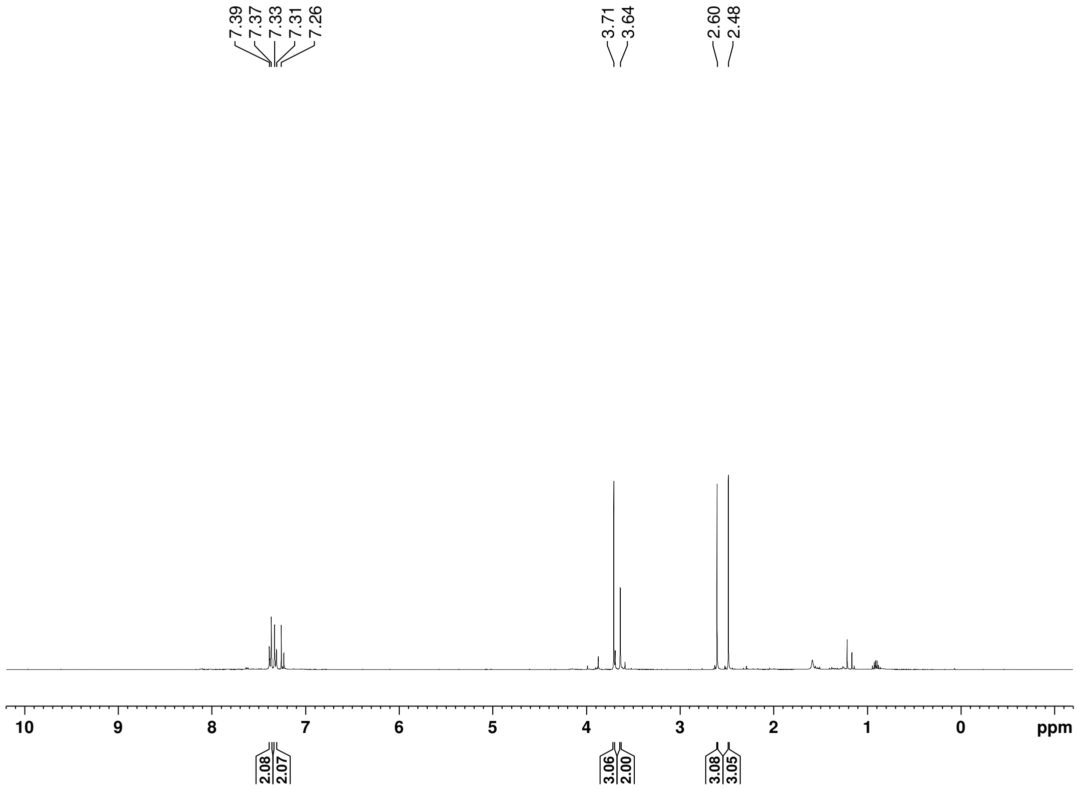

^{13}C spectrum of **6** (101 MHz, $CDCl_3$):

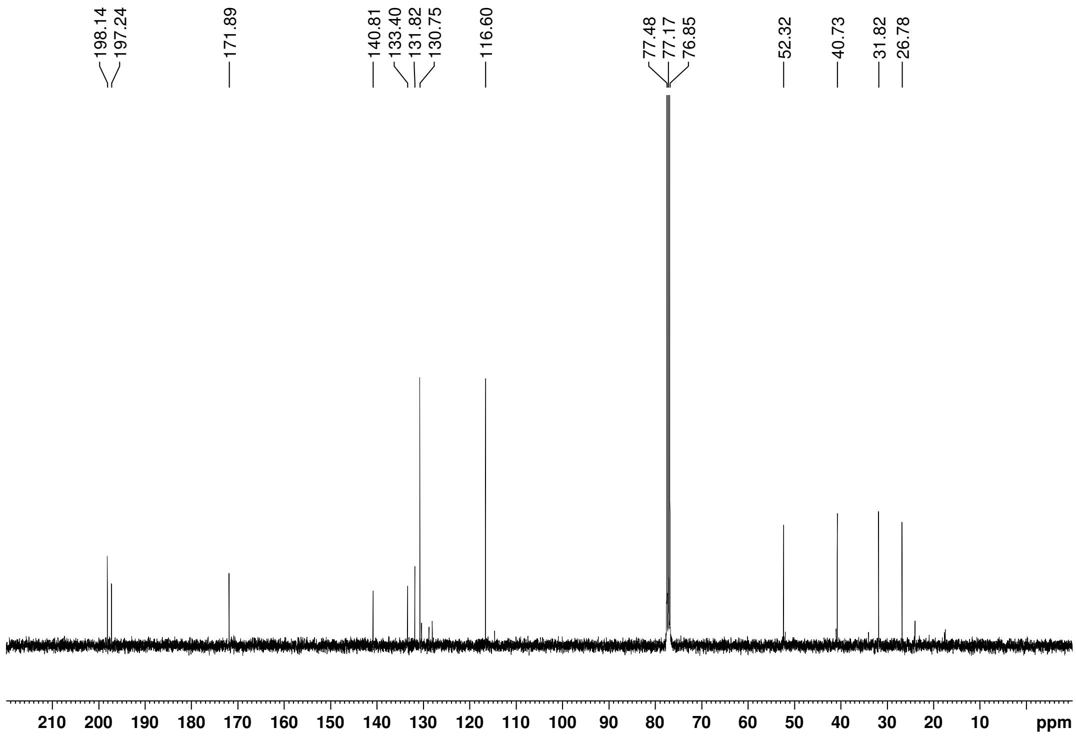

^{1}H spectrum of **7** (400 MHz, $CDCl_3$):

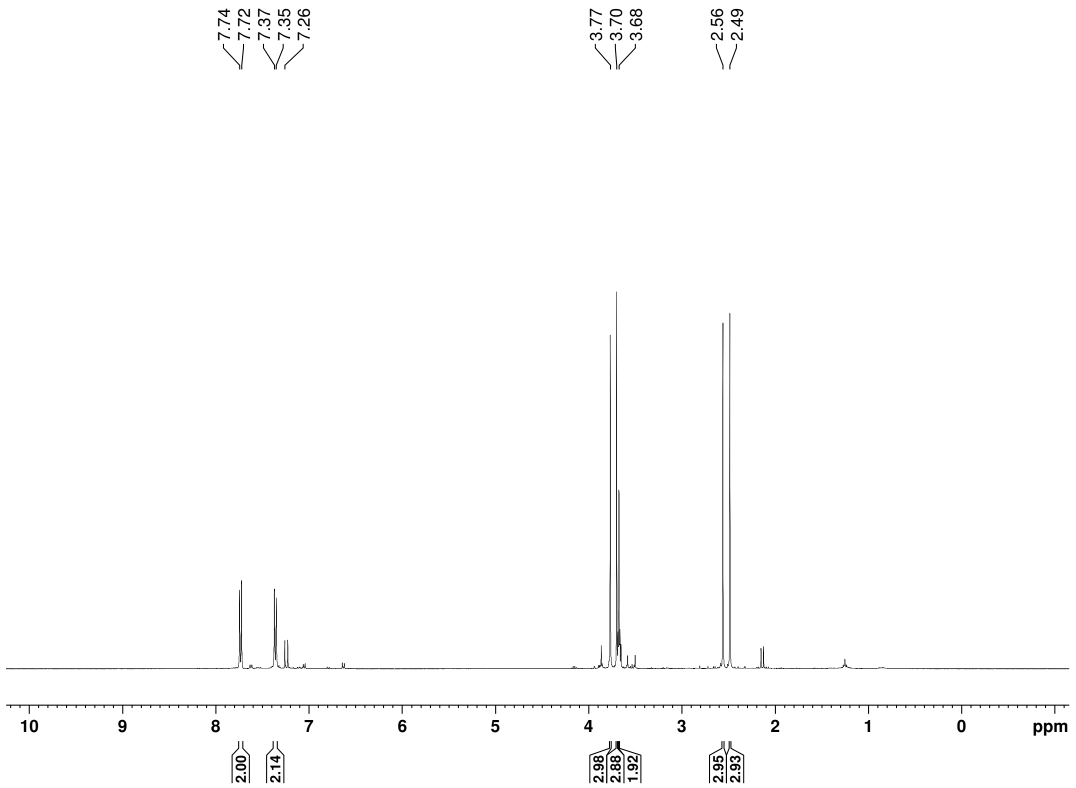

^{13}C spectrum of **7** (101 MHz, $CDCl_3$):

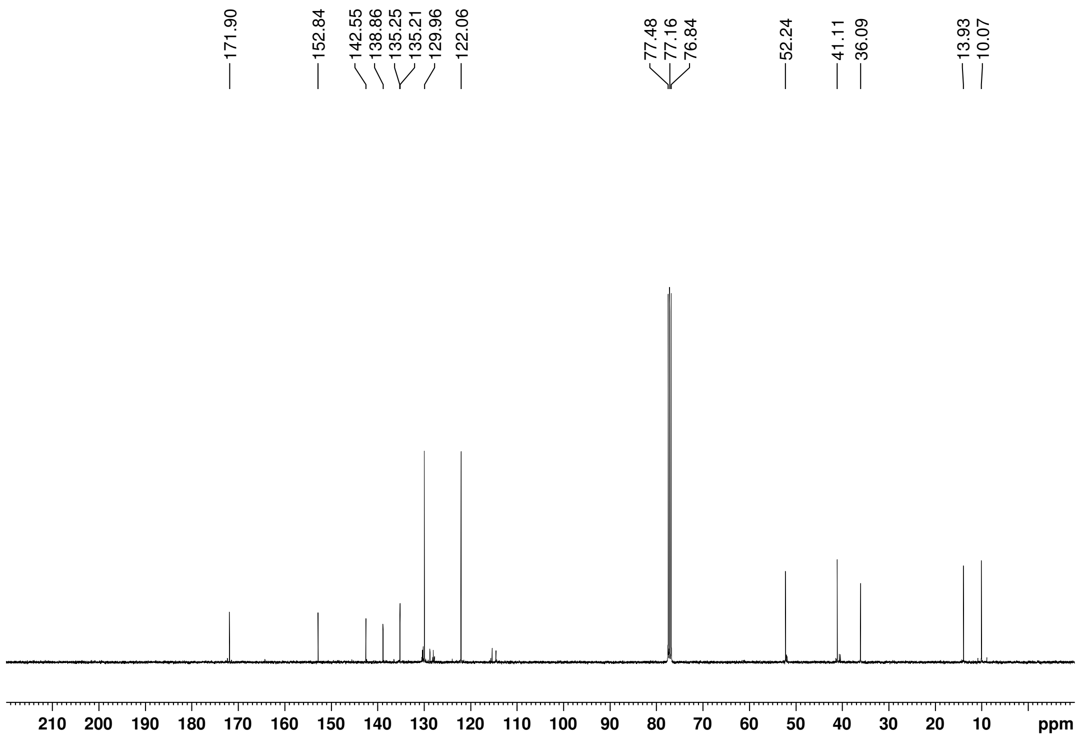

^{1}H spectrum of **8** (400 MHz, MeOD):

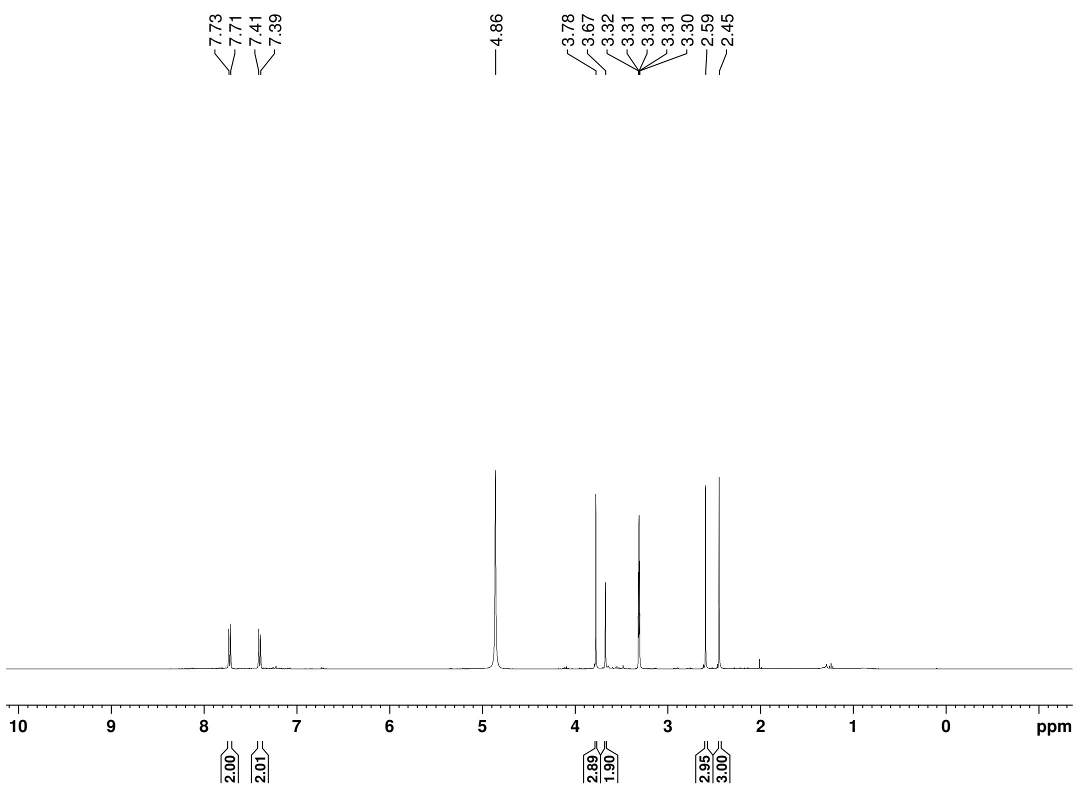

^{13}C spectrum of **8** (101 MHz, MeOD):

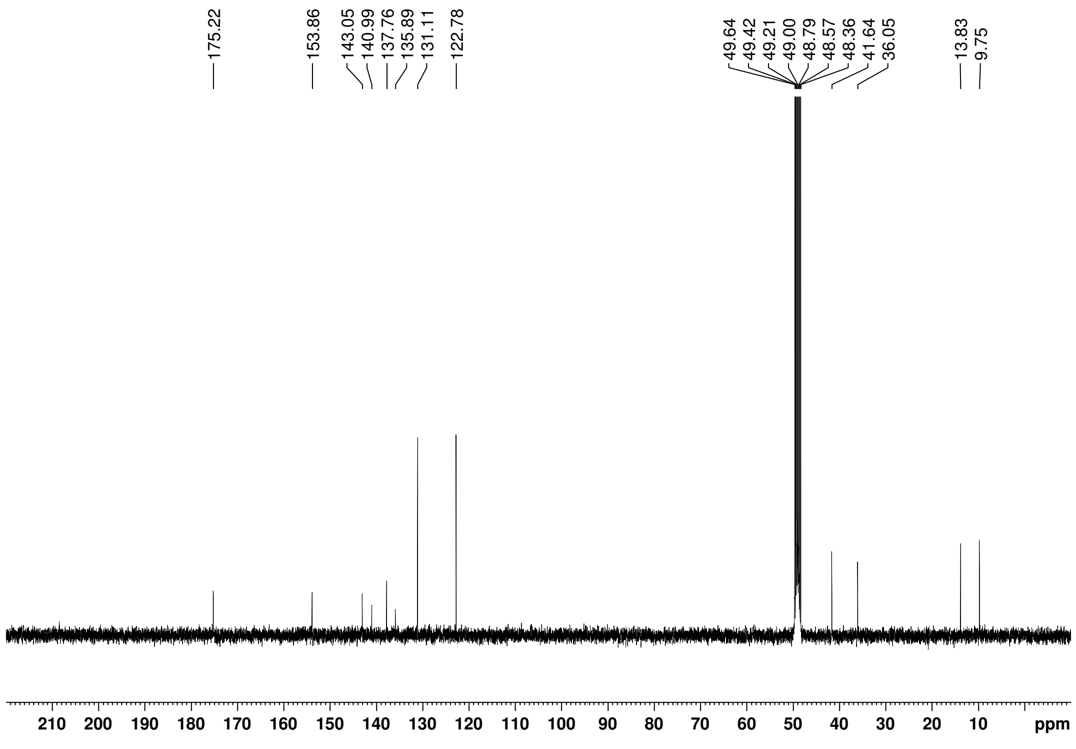

^{1}H spectrum of **9** (400 MHz, $CDCl_3$):

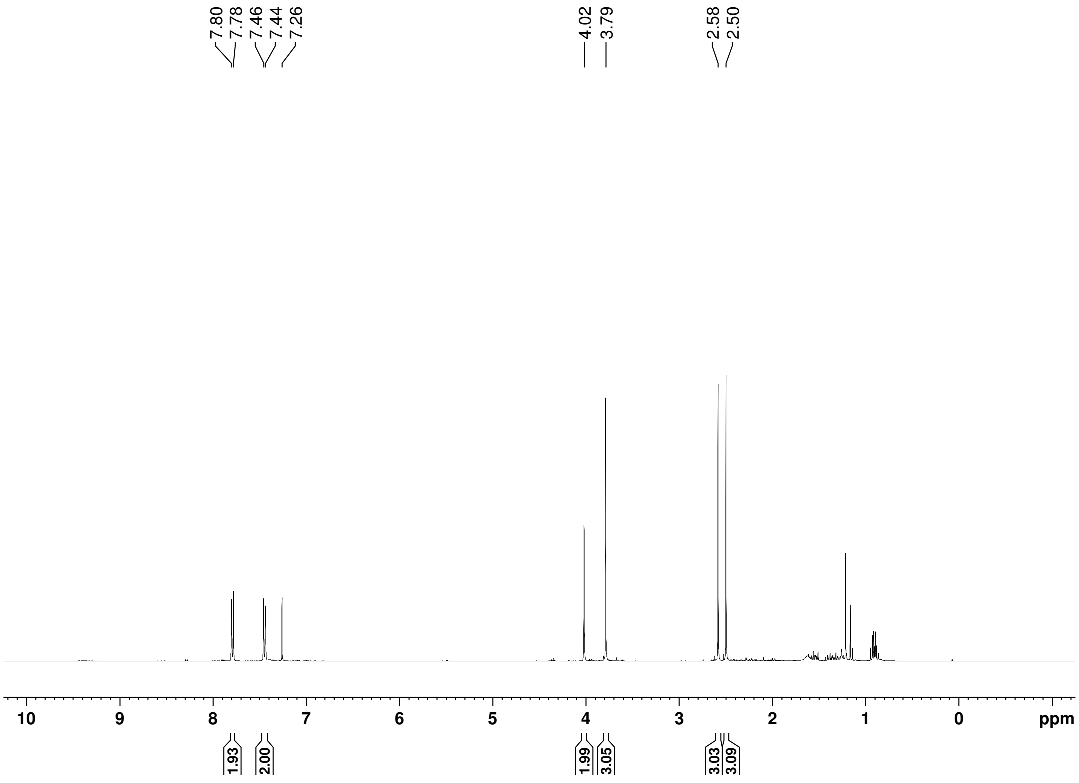

*Contains grease.

^{13}C spectrum of **9** (101 MHz, $CDCl_3$):

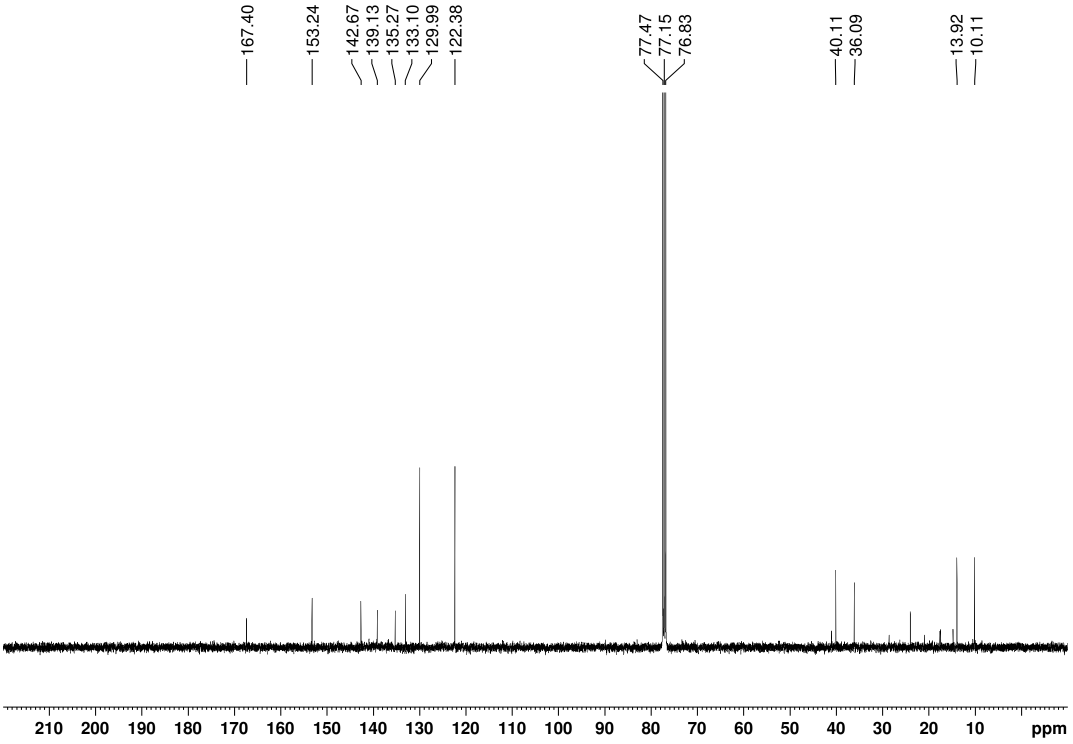

*Contains grease.

^{19}F spectrum of **9** (377 MHz, $CDCl_3$):

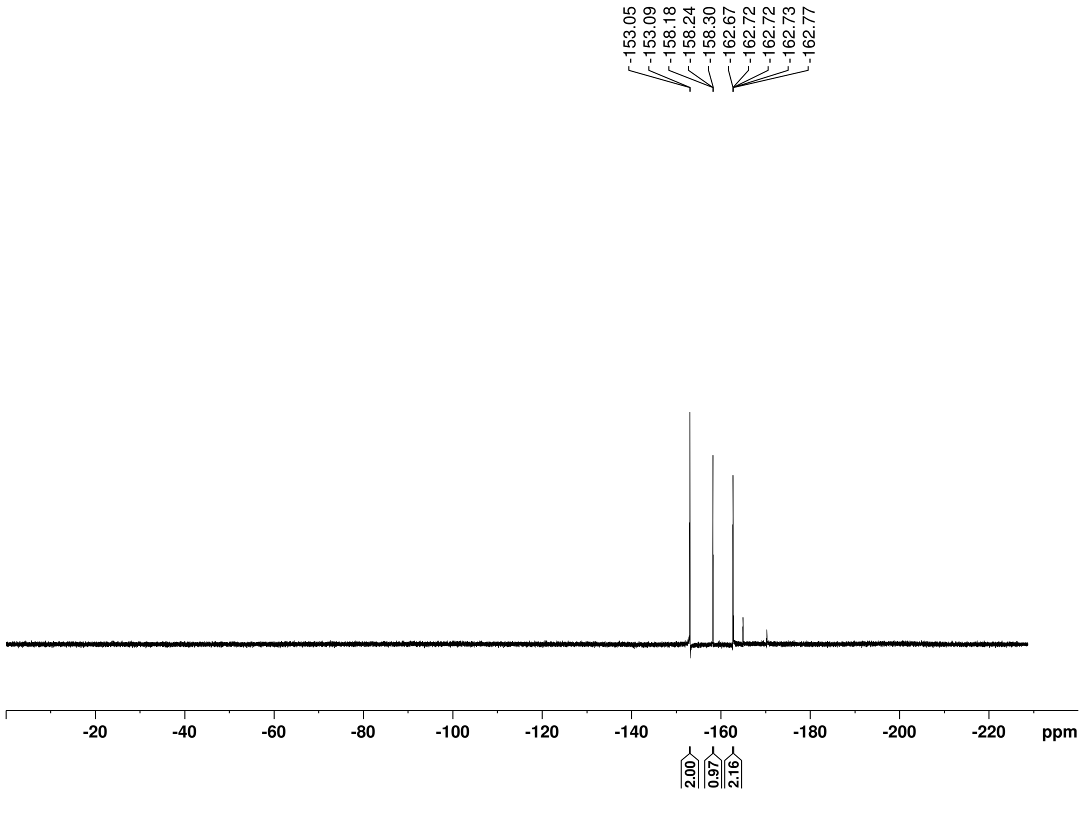

^{1}H spectrum of **1** (400 MHz, MeOD):

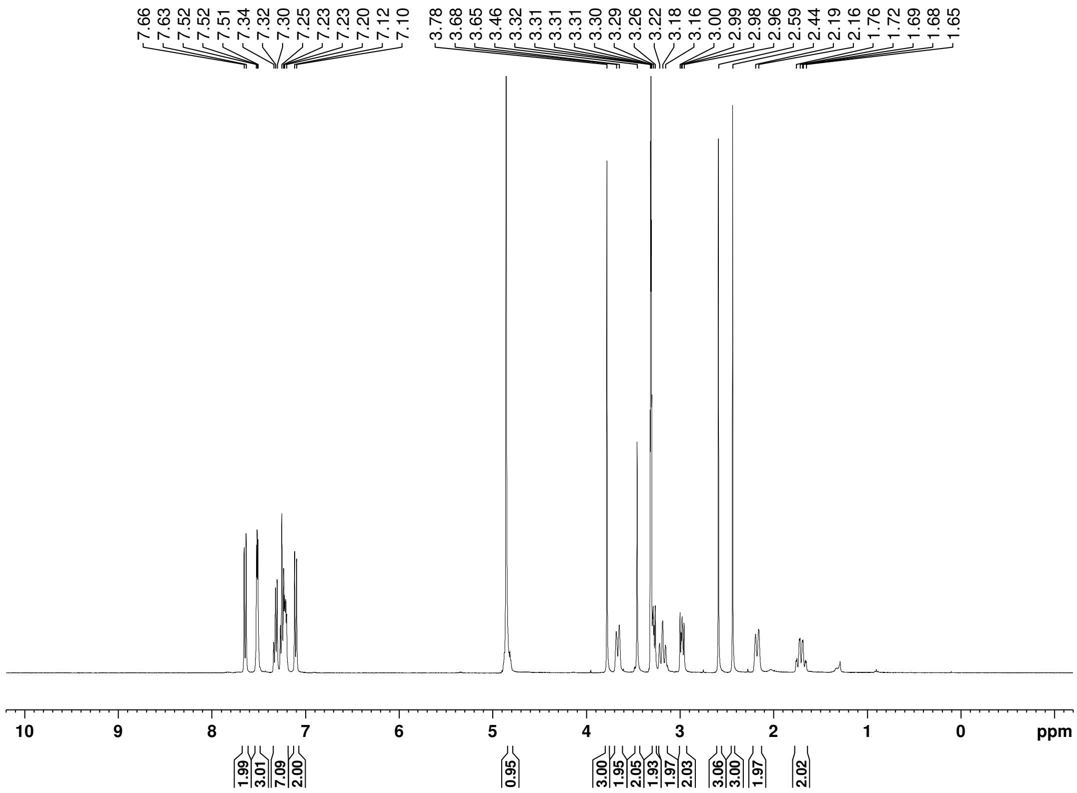

*Contains water present in MeOD.

^{13}C spectrum of **1** (101 MHz, MeOD):

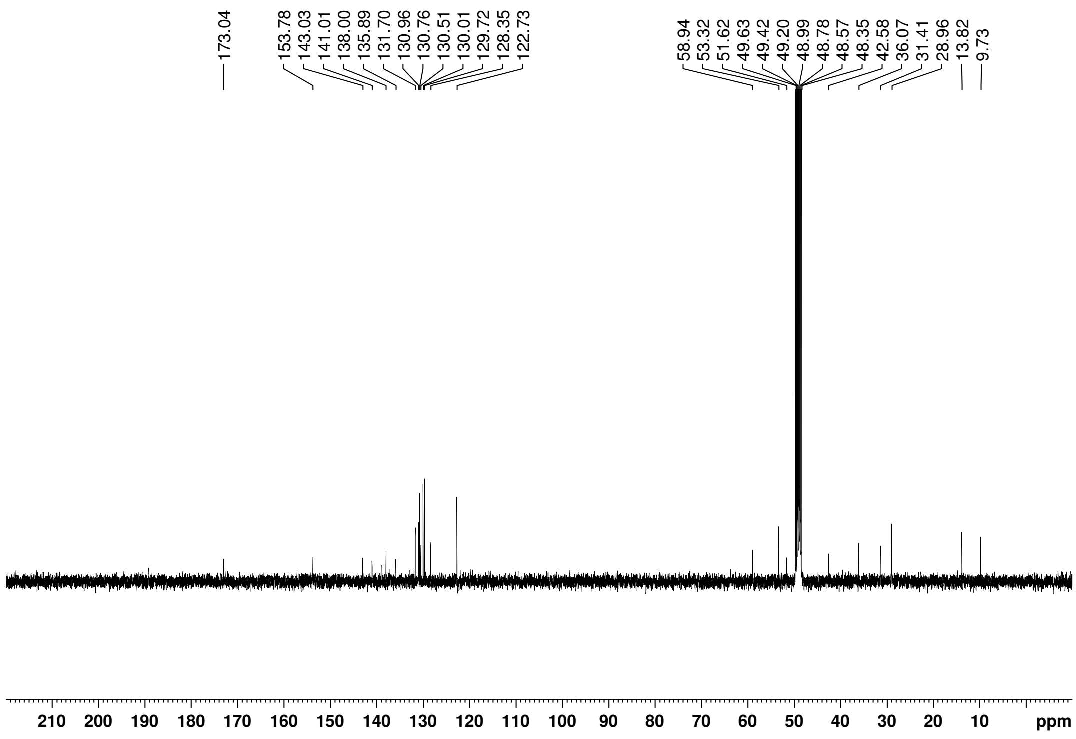

^{1}H spectrum of **11** (400 MHz, $CDCl_3$):

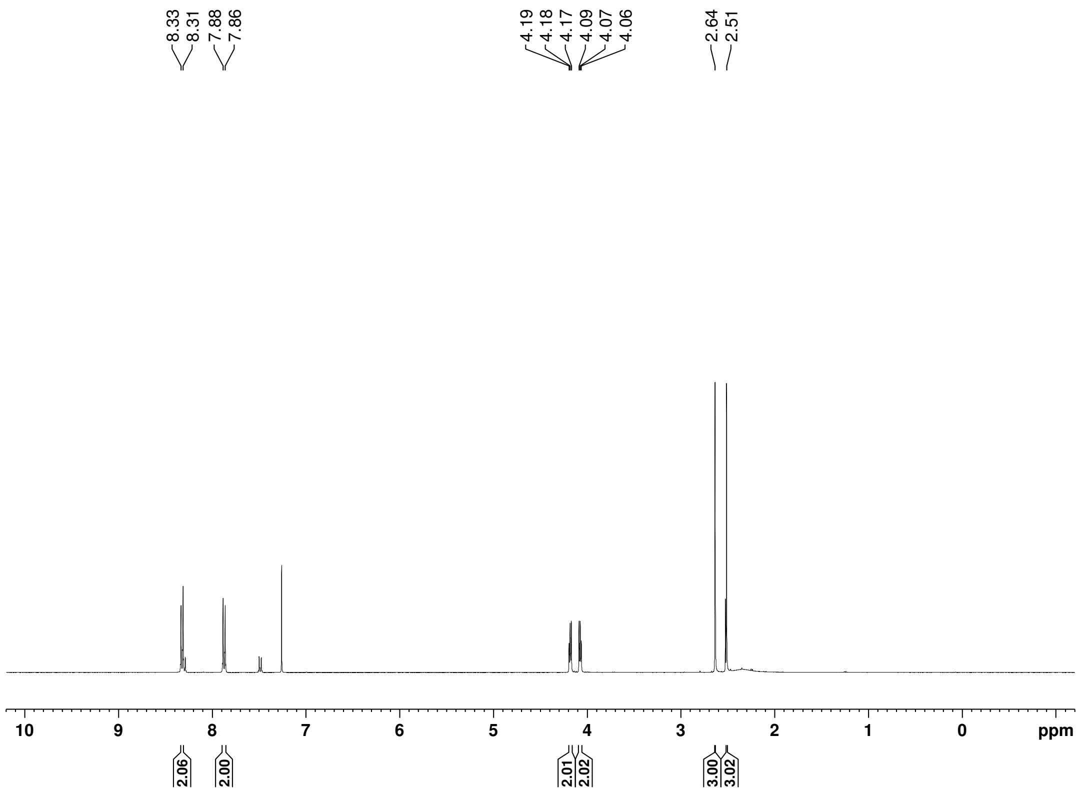

^{13}C spectrum of **11** (101 MHz, $CDCl_3$):

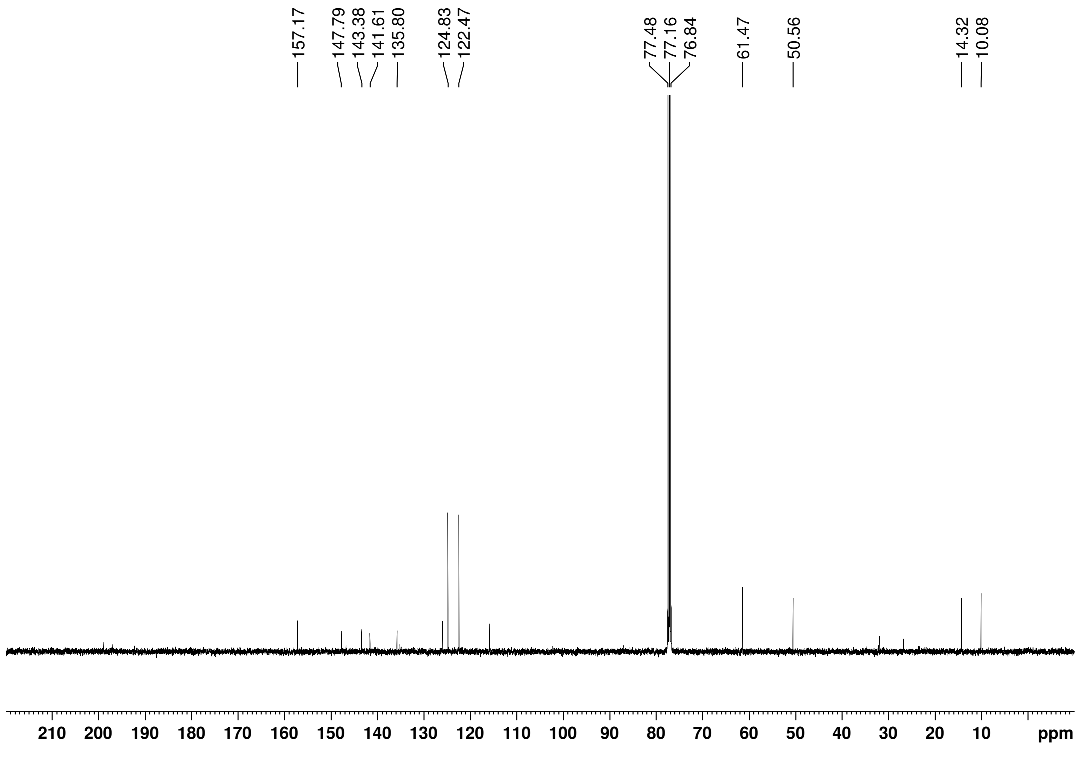

^{1}H spectrum of **12** (400 MHz, DMSO-D_6):

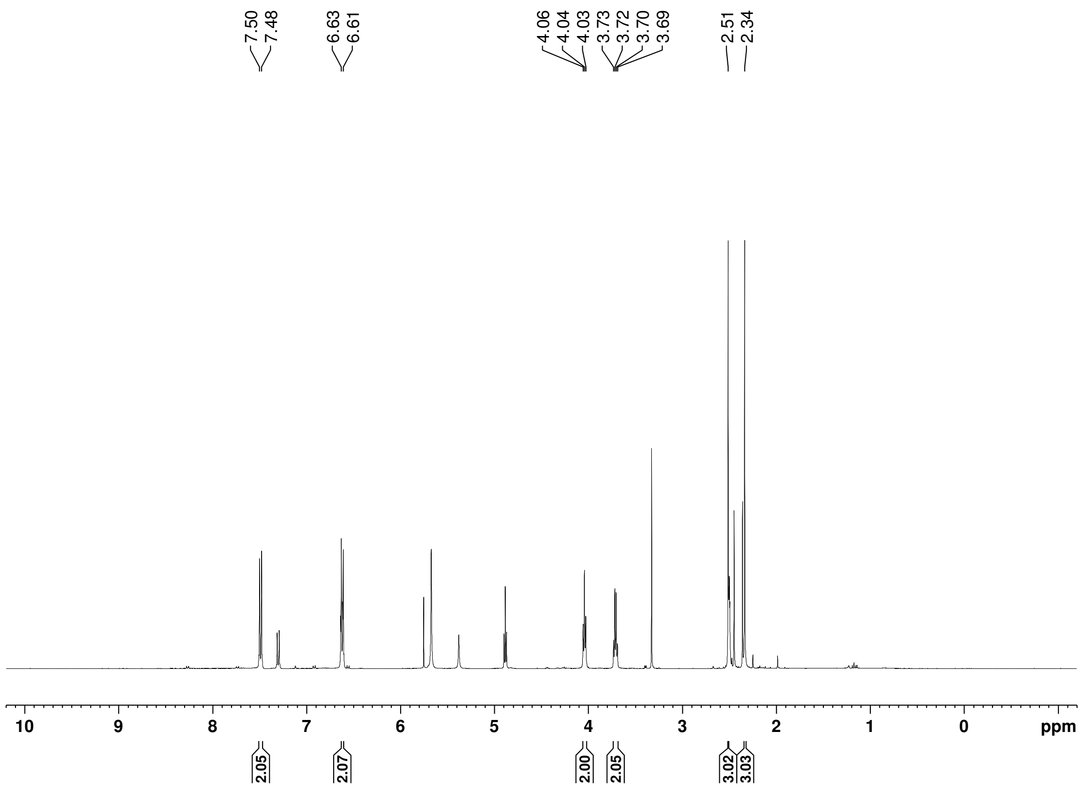

^{13}C spectrum of **12** (101 MHz, DMSO-D_6):

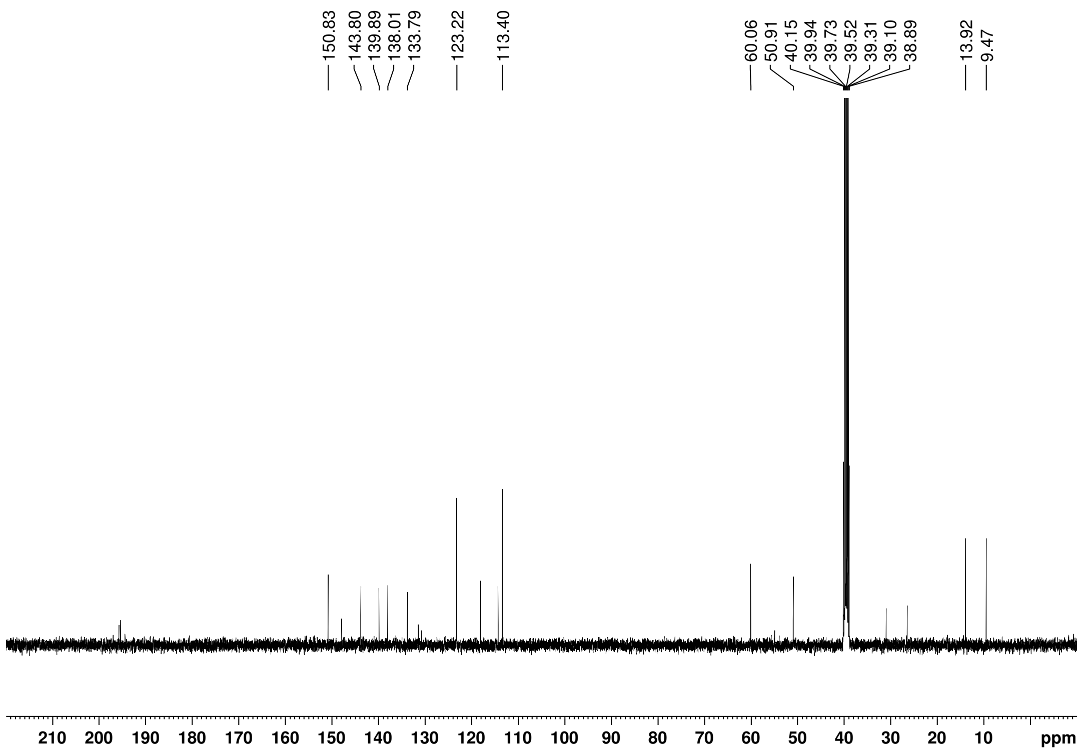

^{1}H spectrum of **13** (400 MHz, DMSO-D$_6$):

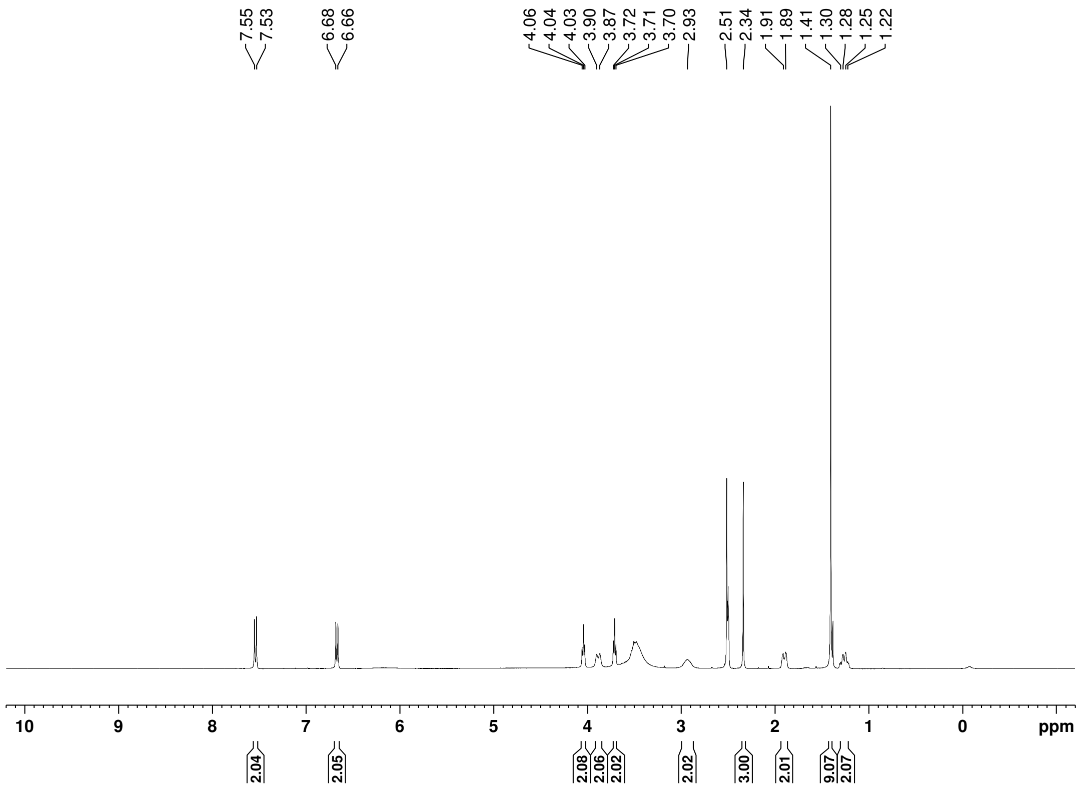

^{13}C spectrum of **13** (101 MHz, DMSO-D$_6$):

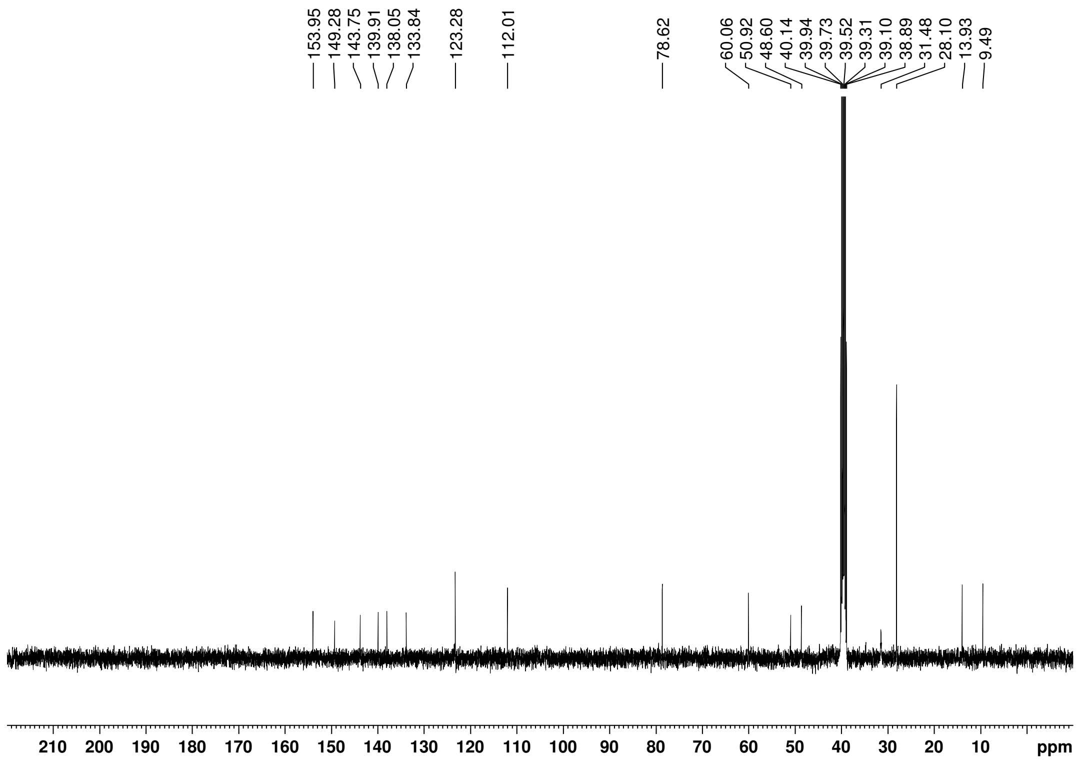

^{1}H spectrum of **14** (400 MHz, $CDCl_3$):

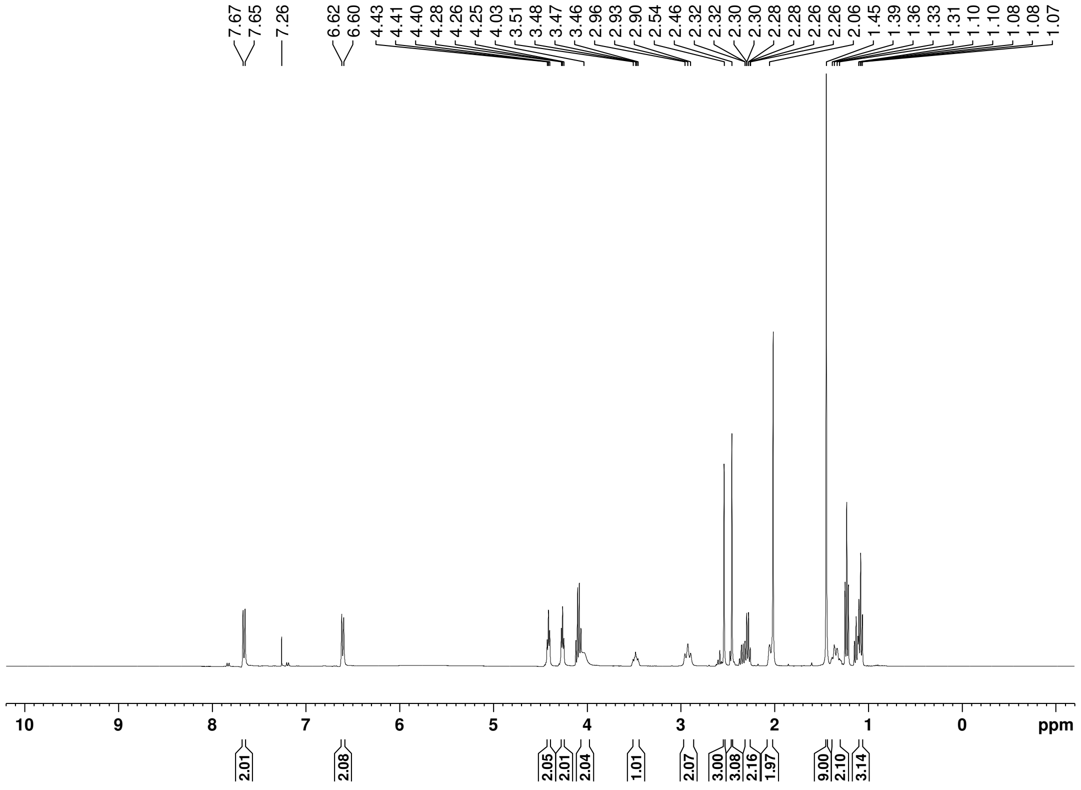

*Contains ethyl acetate

^{13}C spectrum of **14** (101 MHz, $CDCl_3$):

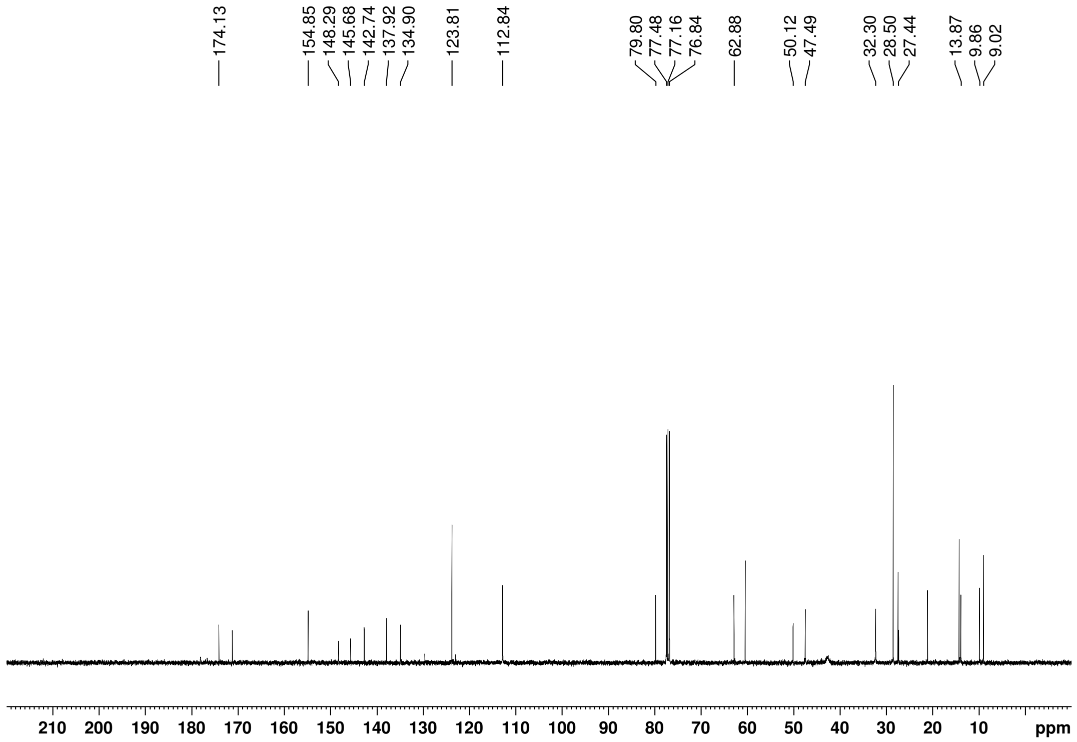

*Contains ethyl acetate

^{1}H spectrum of **15** (400 MHz, DMSO-D6):

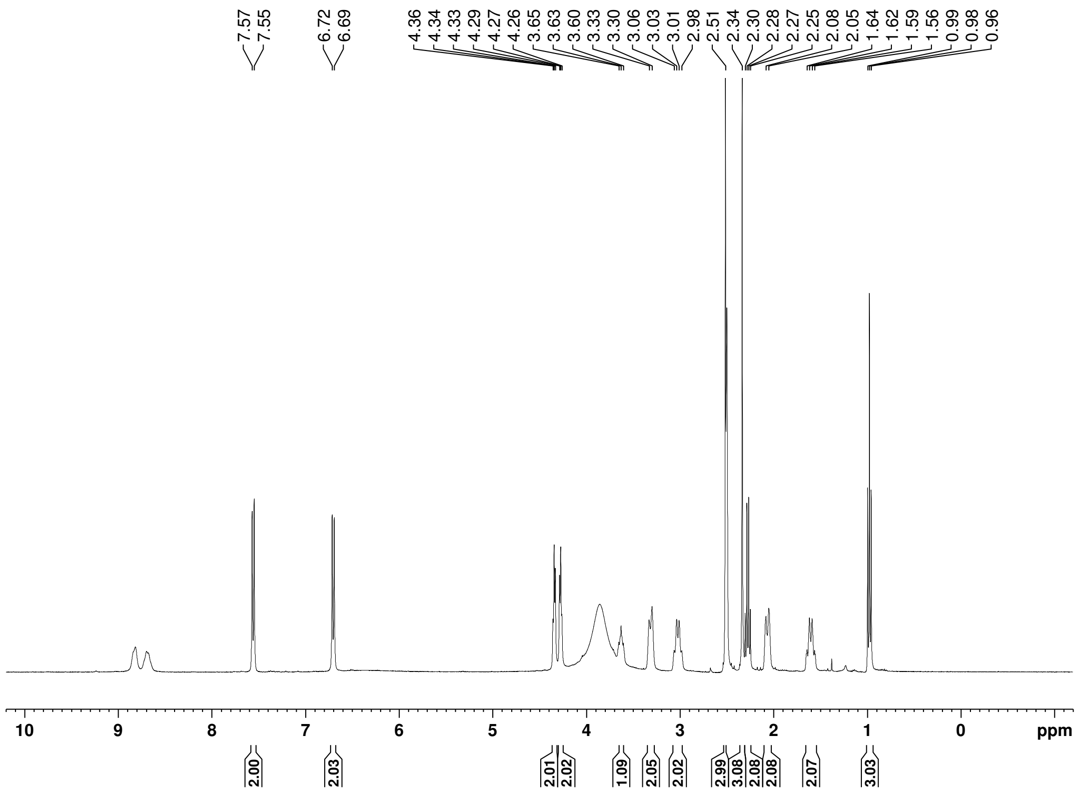

^{13}C spectrum of **15** (101 MHz, DMSO-D6):

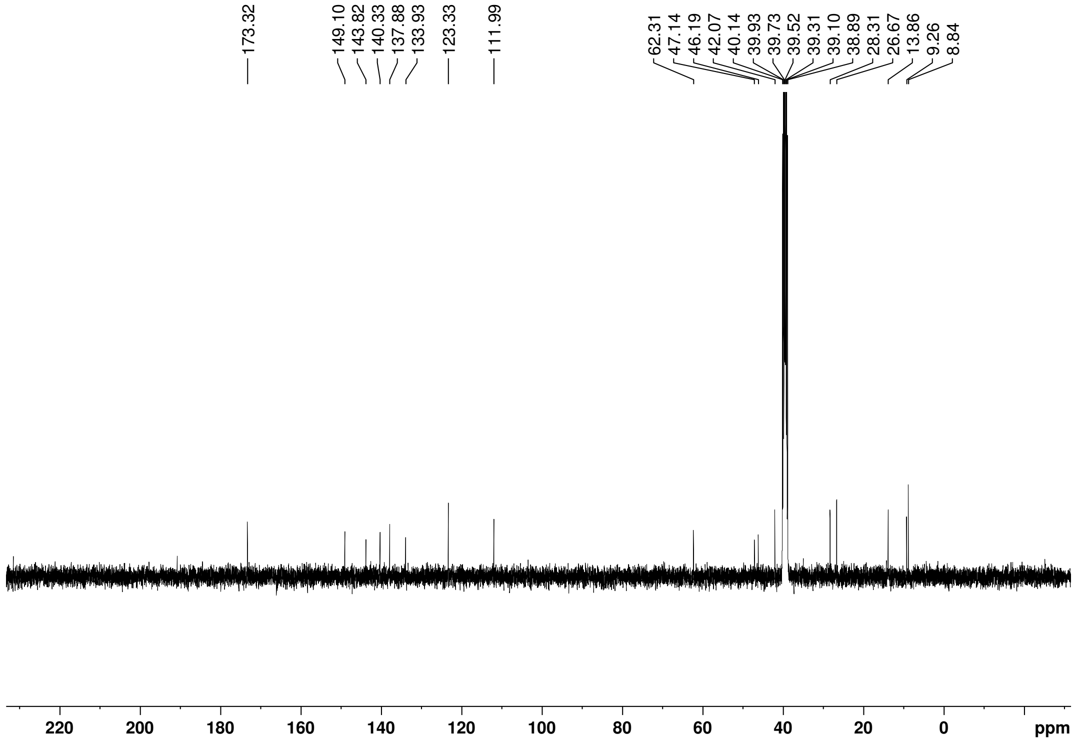

^{1}H spectrum of **2** (400 MHz, MeOD):

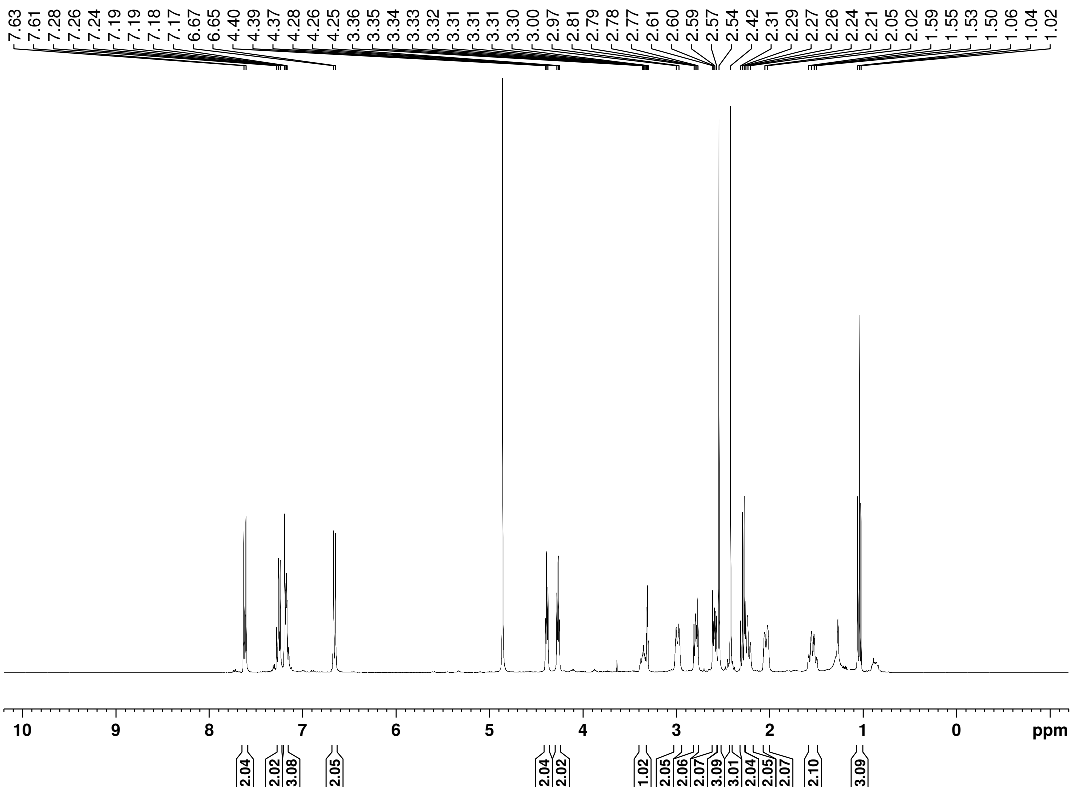

*Contains water that was present in MeOD and trace amounts of grease.

^{13}C spectrum of **2** (101 MHz, MeOD):

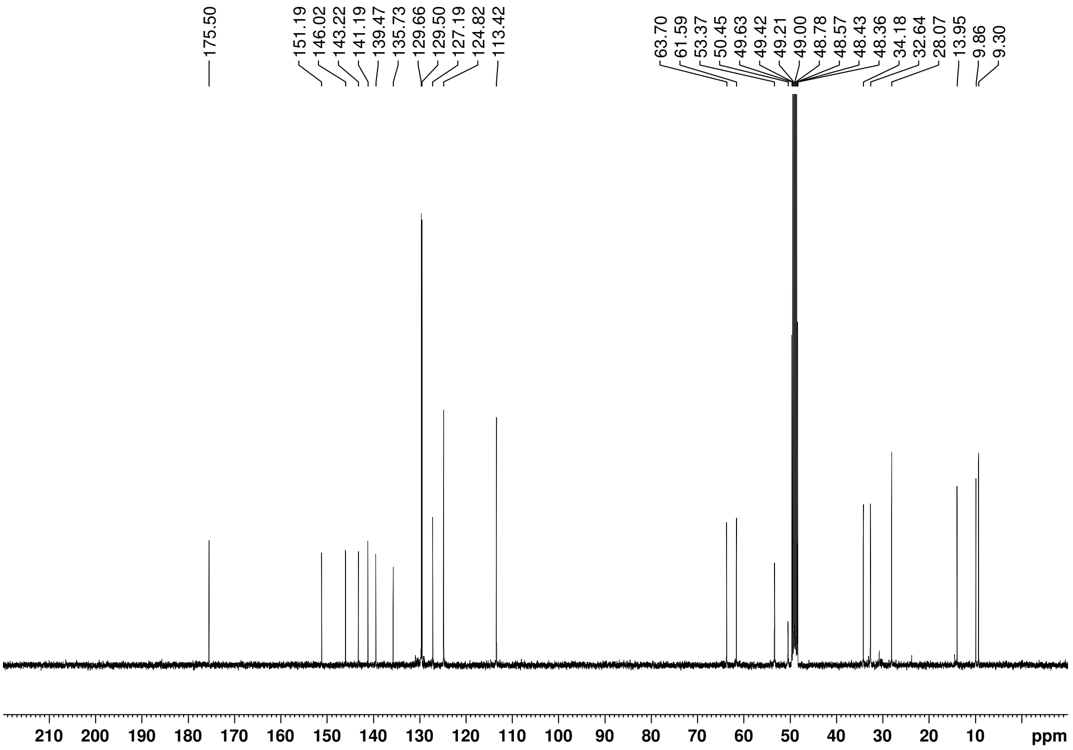

^{1}H spectrum of **17** (400 MHz, $CDCl_3$):

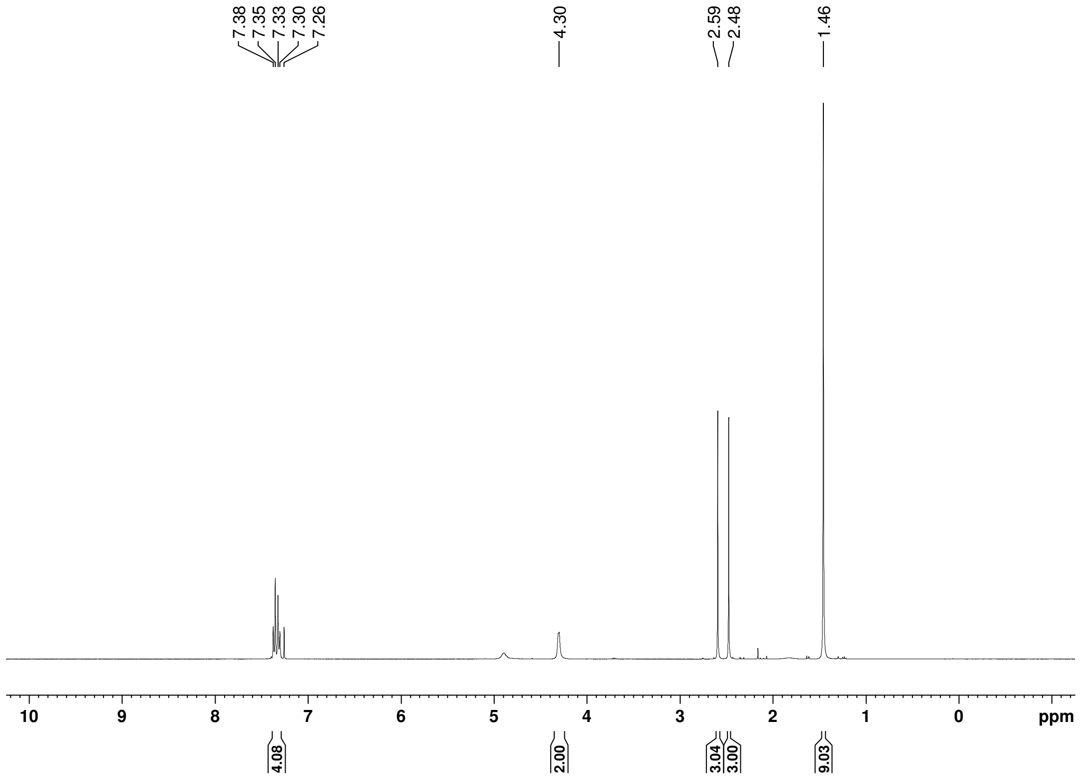

^{13}C spectrum of **17** (101 MHz, $CDCl_3$):

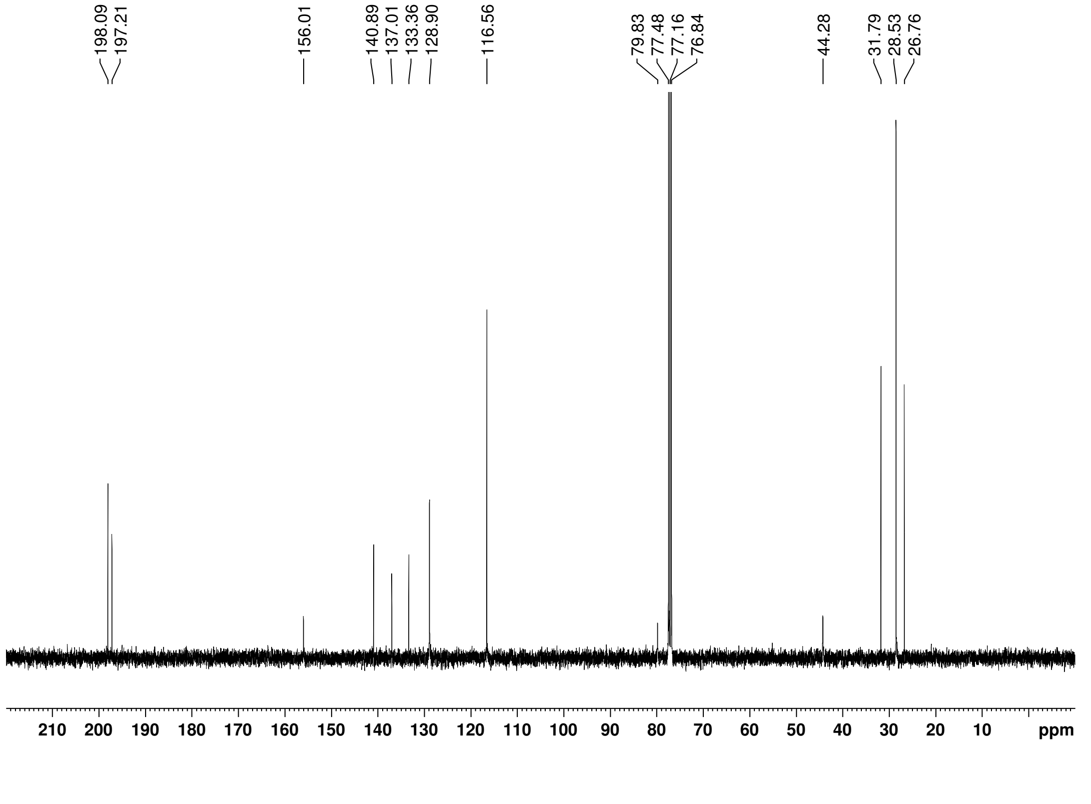

^{1}H spectrum of **18** (400 MHz, $CDCl_3$):

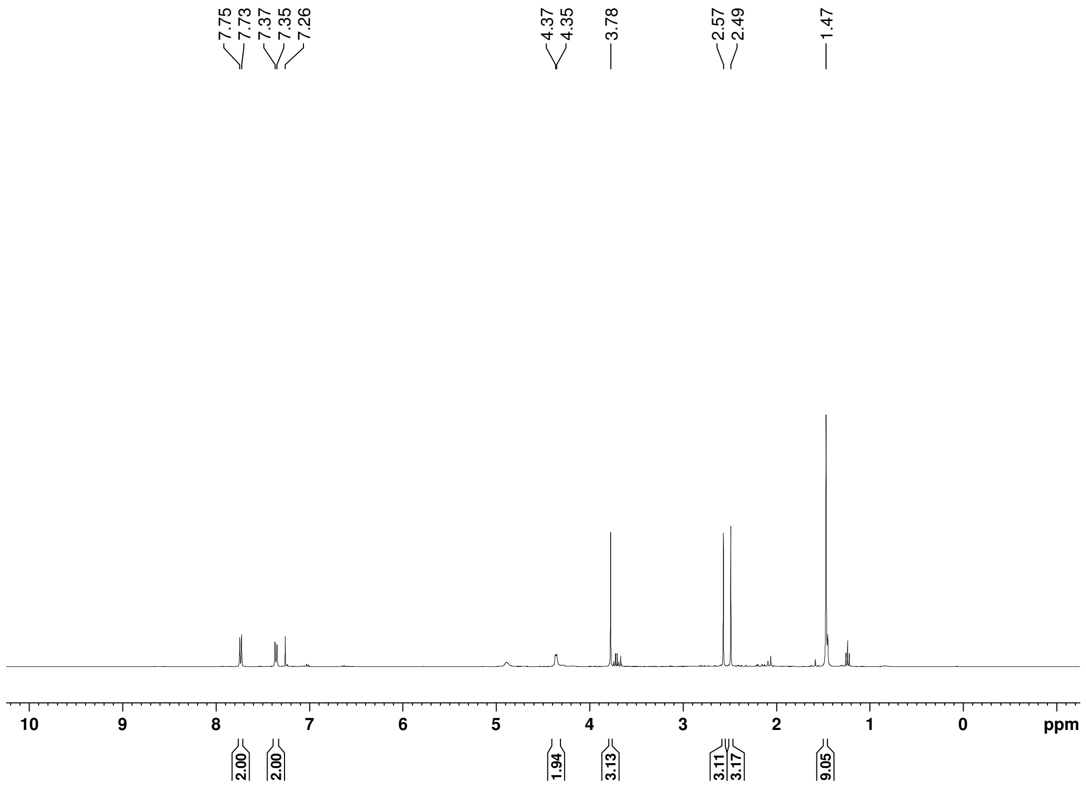

*Contains ethanol. Yield was obtained after further drying.

^{13}C spectrum of **18** (101 MHz, $CDCl_3$):

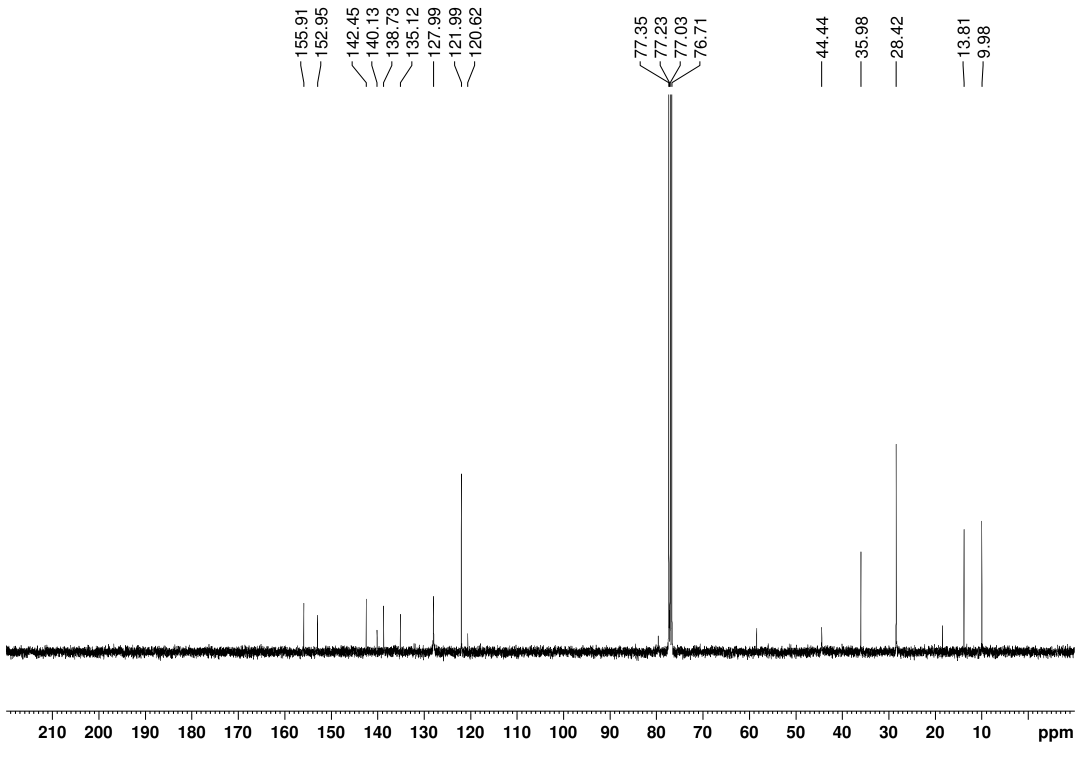

*Contains ethanol. Yield was obtained after further drying.

^{1}H spectrum of **19** (400 MHz, MeOD):

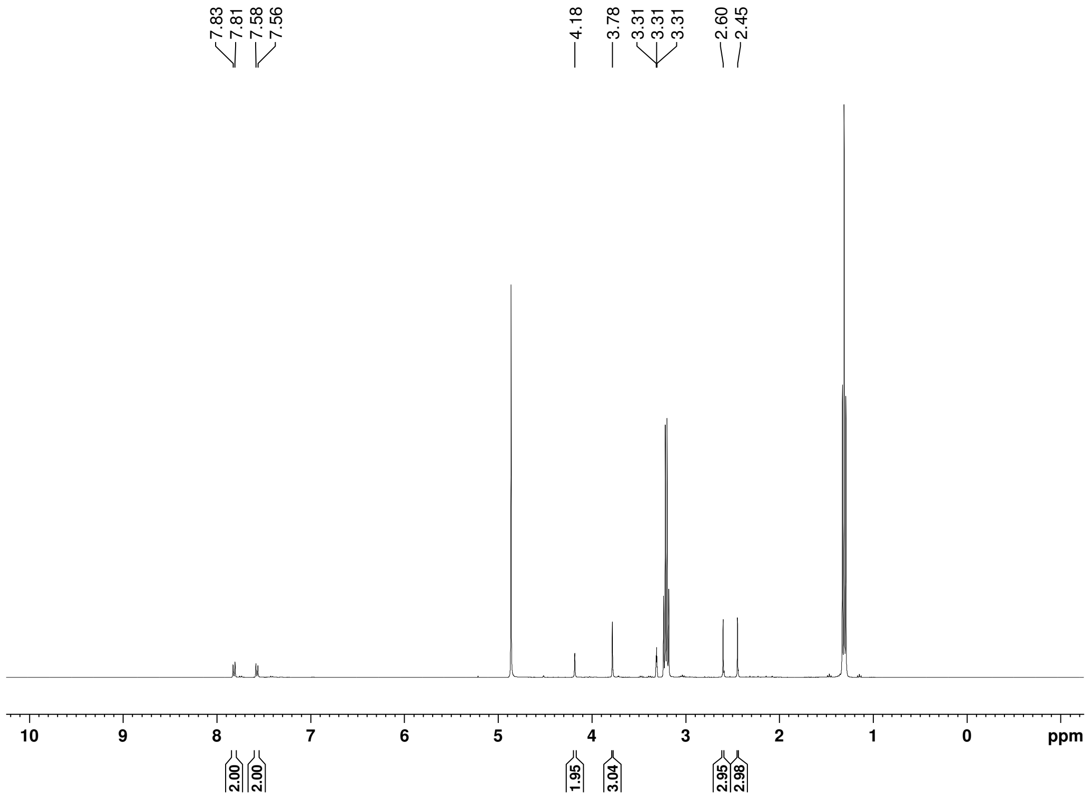

*Contains water and diethyl ether. Yield was obtained after further drying.

^{13}C spectrum of **19** (101 MHz, MeOD):

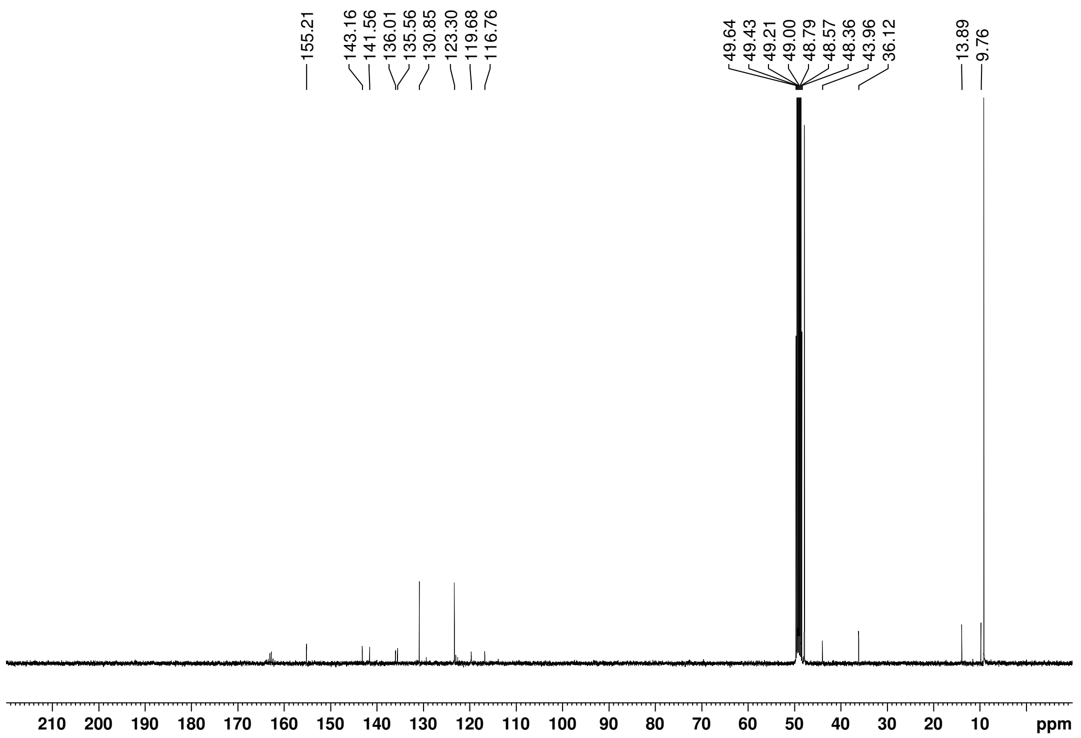

*Contains diethyl ether. Yield was obtained after further drying.

^{1}H spectrum of **20** (400 MHz, $CDCl_3$):

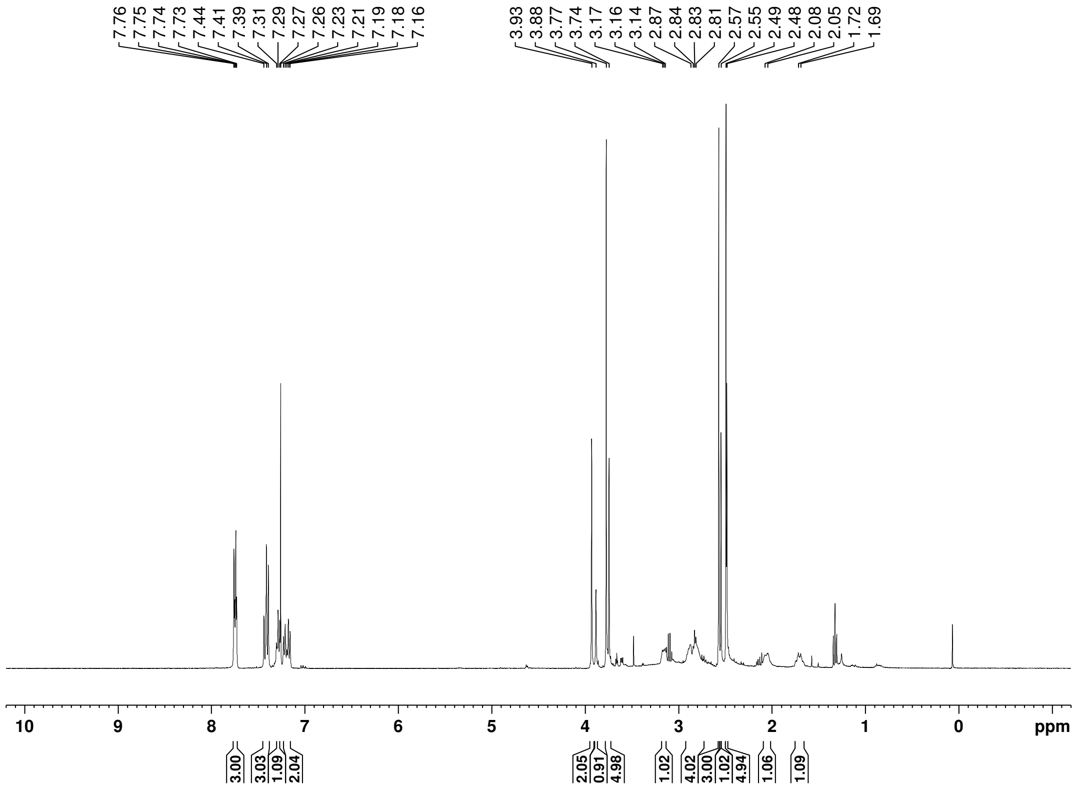

*Contains trace amount of grease and solvents that were used during synthesis procedure.

^{13}C spectrum of **20** (101 MHz, MeOD):

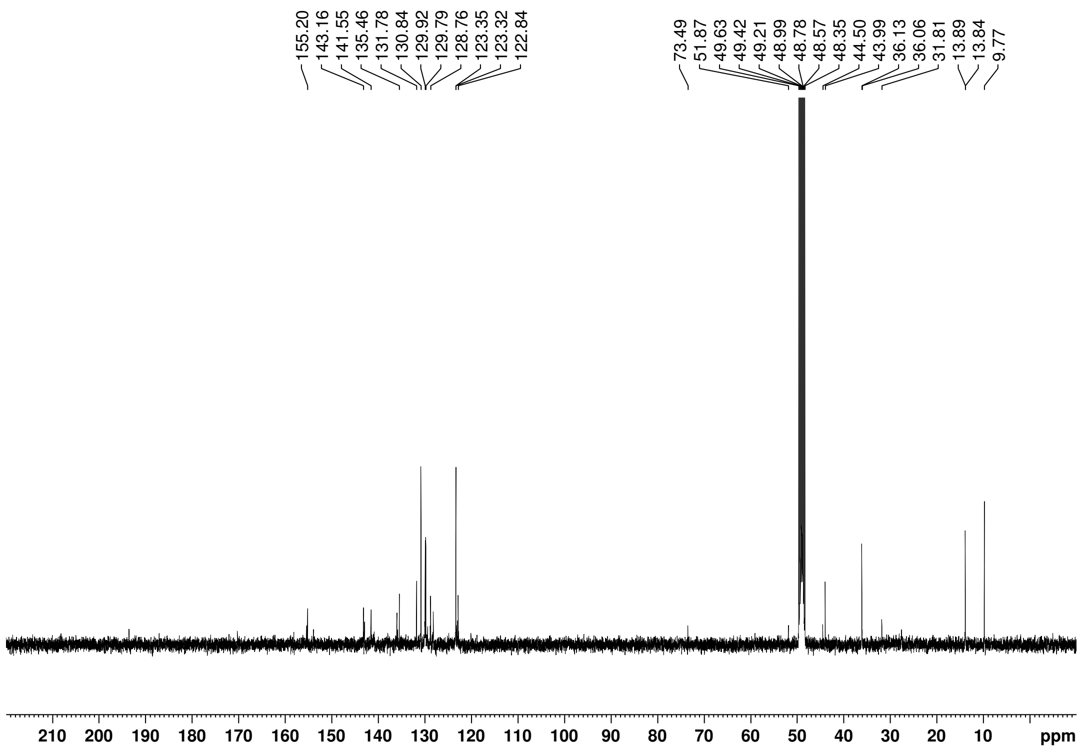

^{1}H spectrum of **3a** (400 MHz, MeOD):

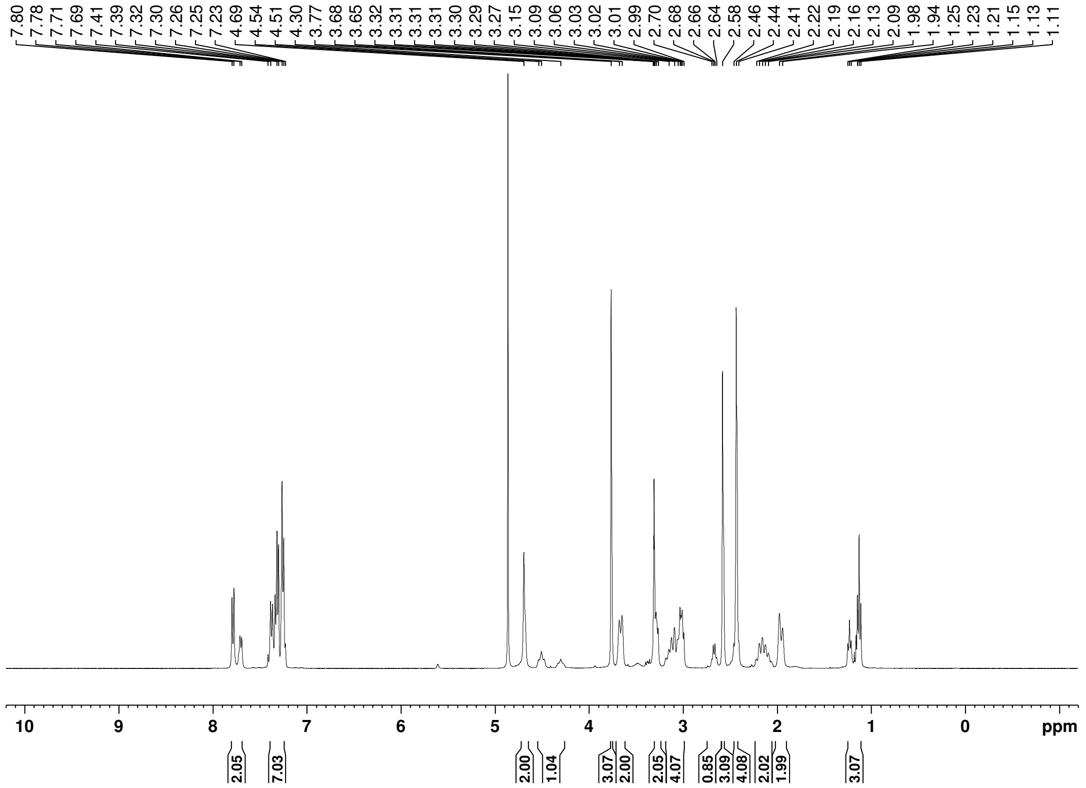

^{13}C spectrum of **3a** (101 MHz, MeOD):

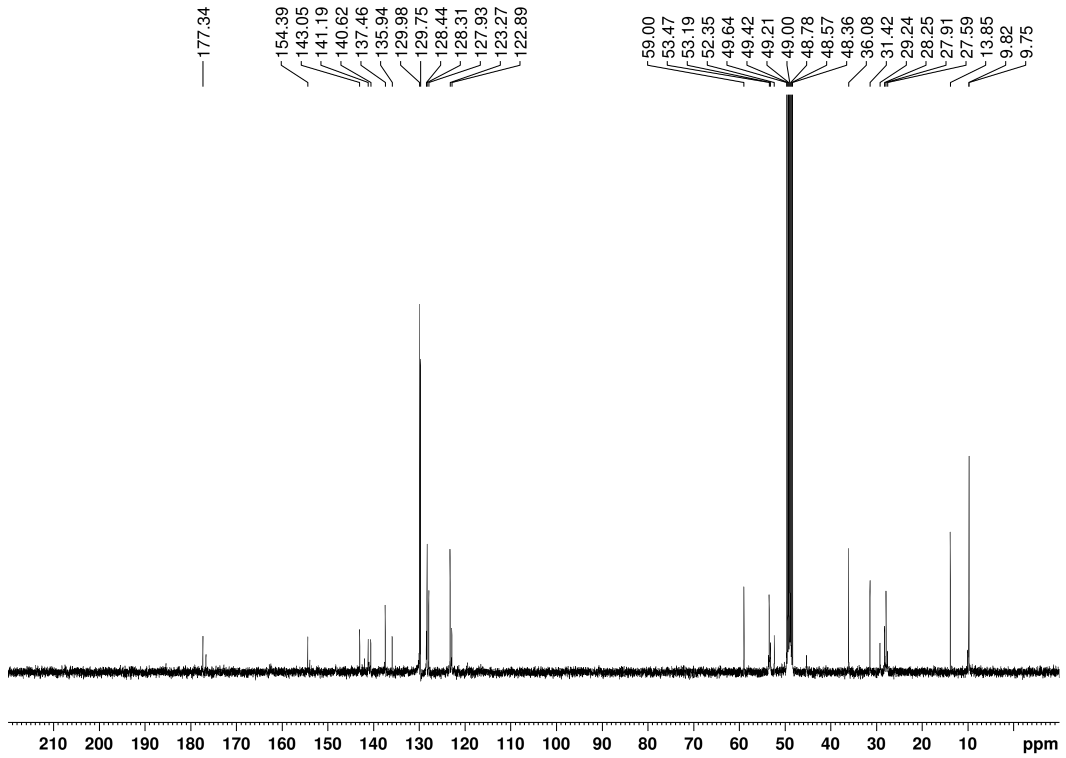

^{1}H spectrum of **21** (400 MHz, $CDCl_3$):

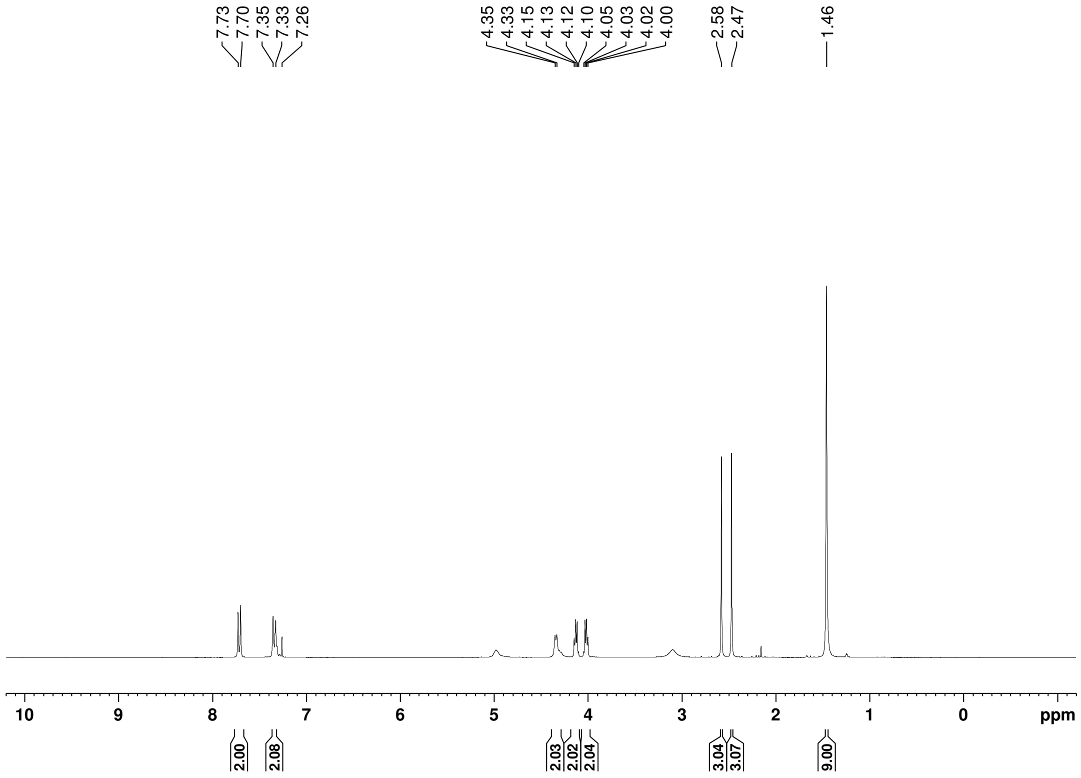

^{13}C spectrum of **21** (101 MHz, $CDCl_3$):

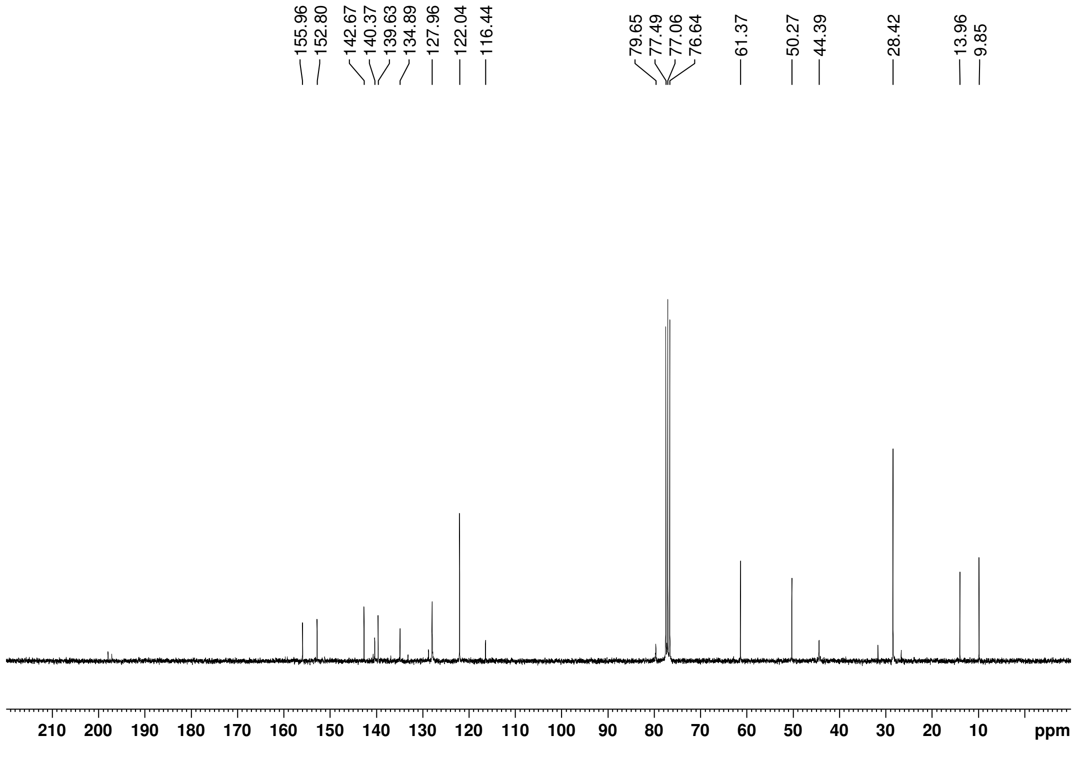

^{1}H spectrum of **22** (400 MHz, $CDCl_3$):

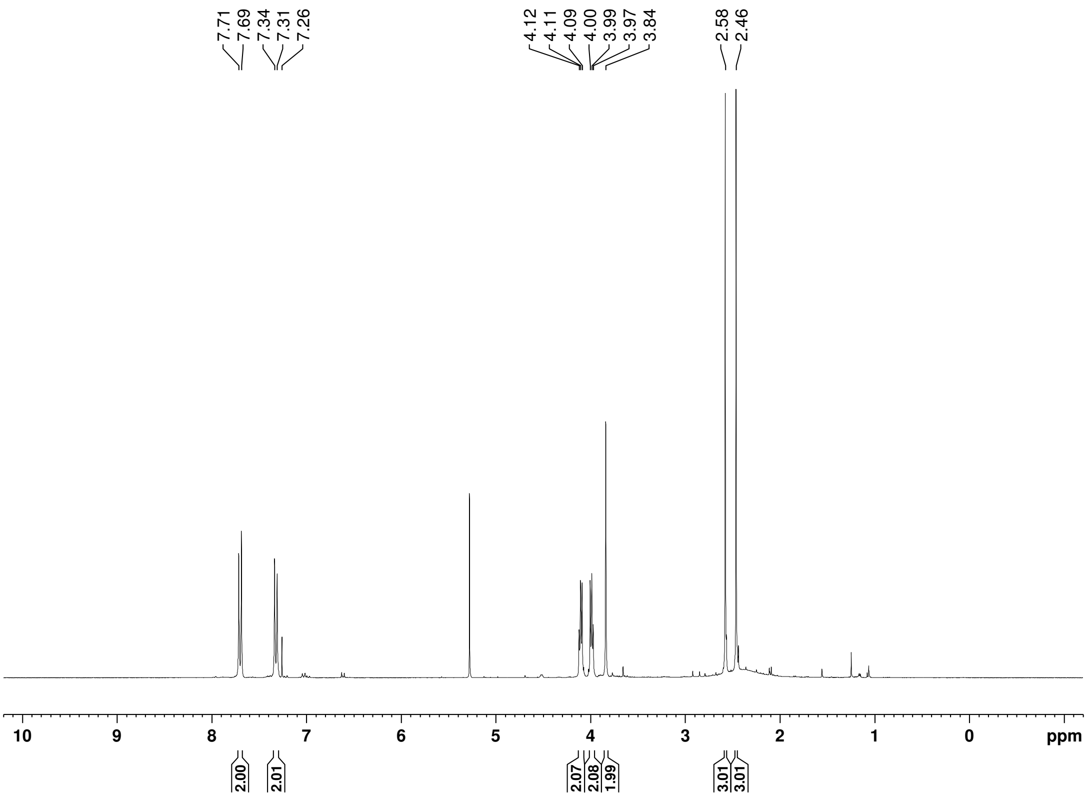

*Contains traces of DCM

^{13}C spectrum of **22** (101 MHz, $CDCl_3$):

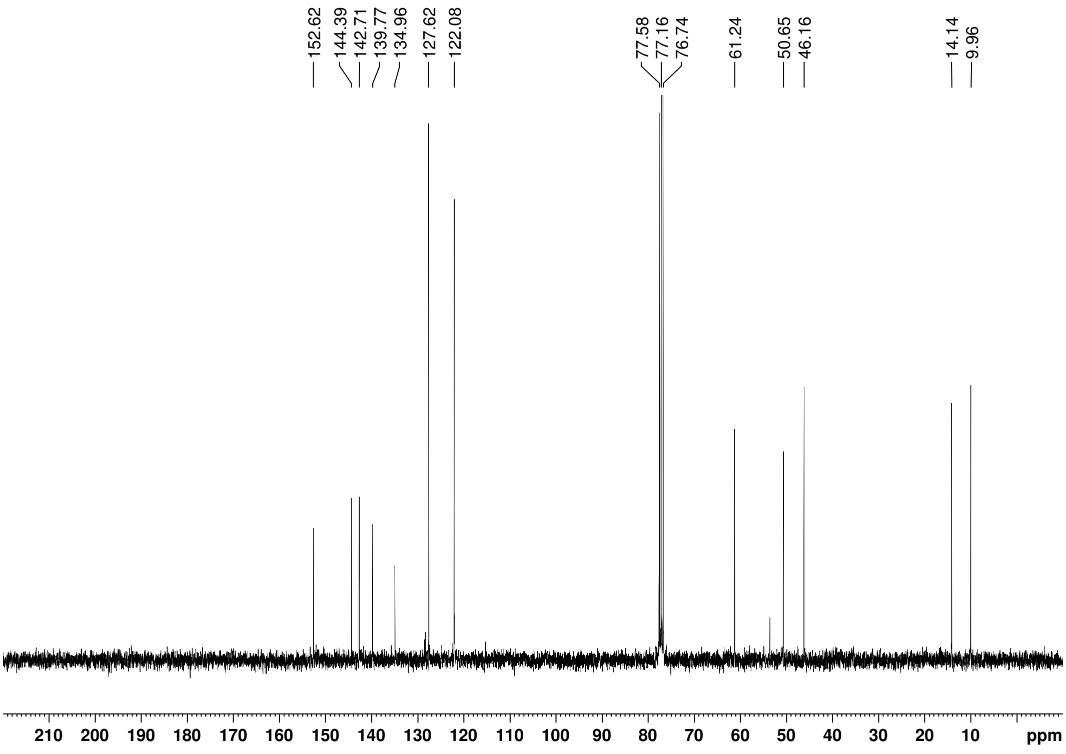

*Contains traces of DCM

^1H spectrum of **23** (400 MHz, $CDCl_3$):

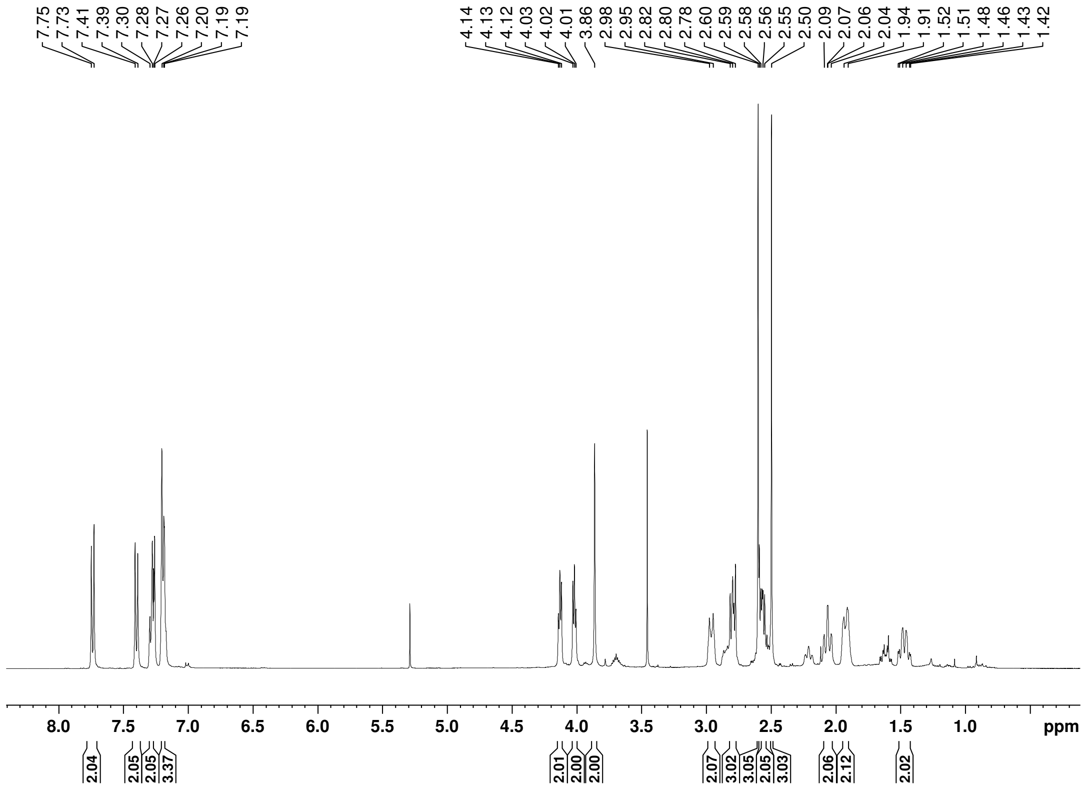

^{13}C spectrum of **23** (101 MHz, $CDCl_3$):

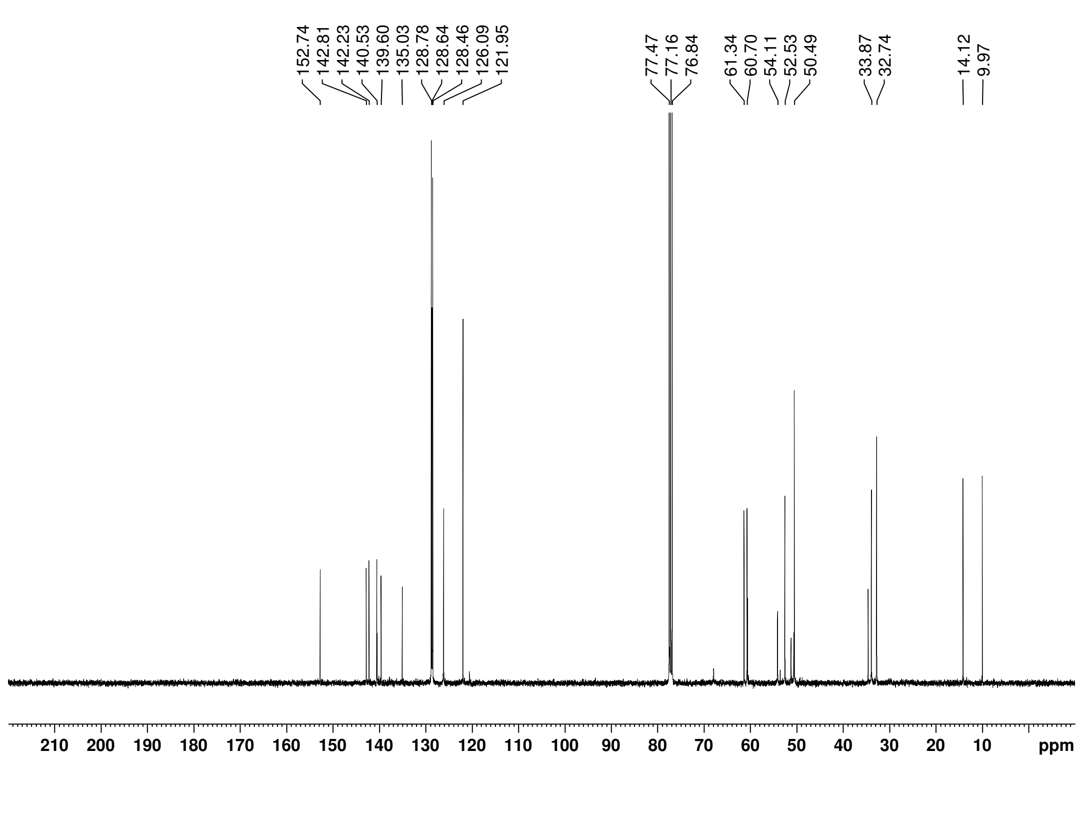

^{1}H spectrum of **3b** (400 MHz, $CDCl_3$):

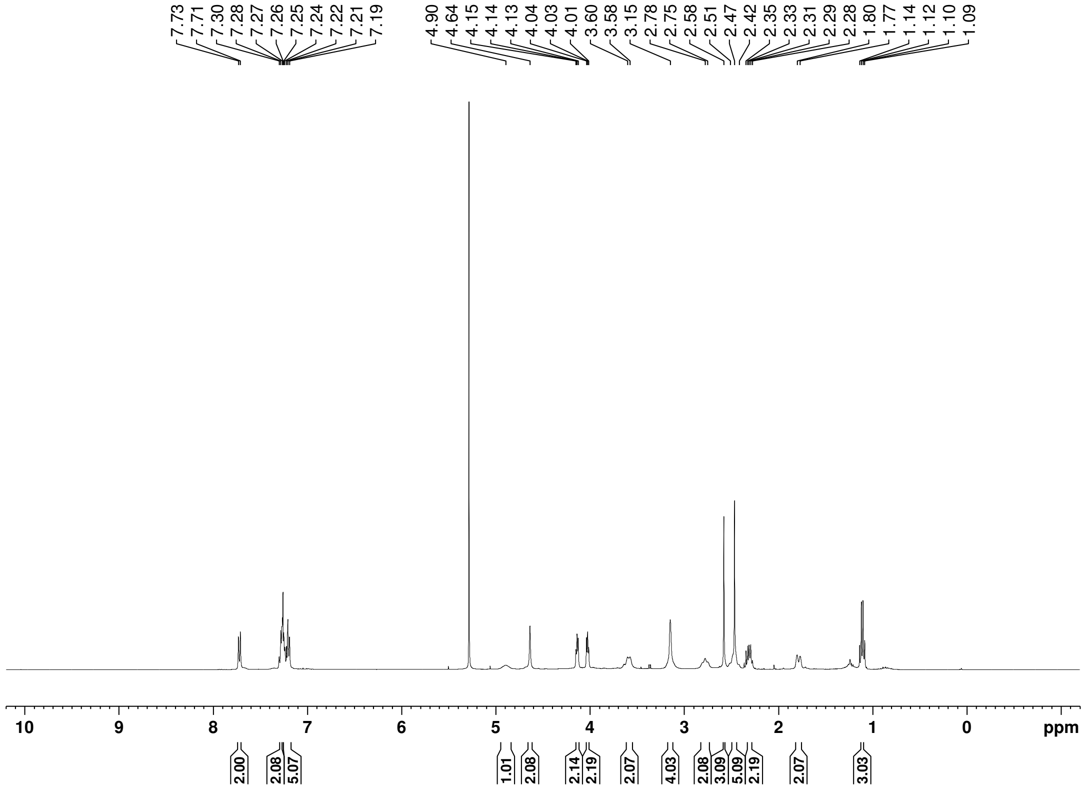

*Contains DCM.

^{13}C spectrum of **3b** (101 MHz, $CDCl_3$):

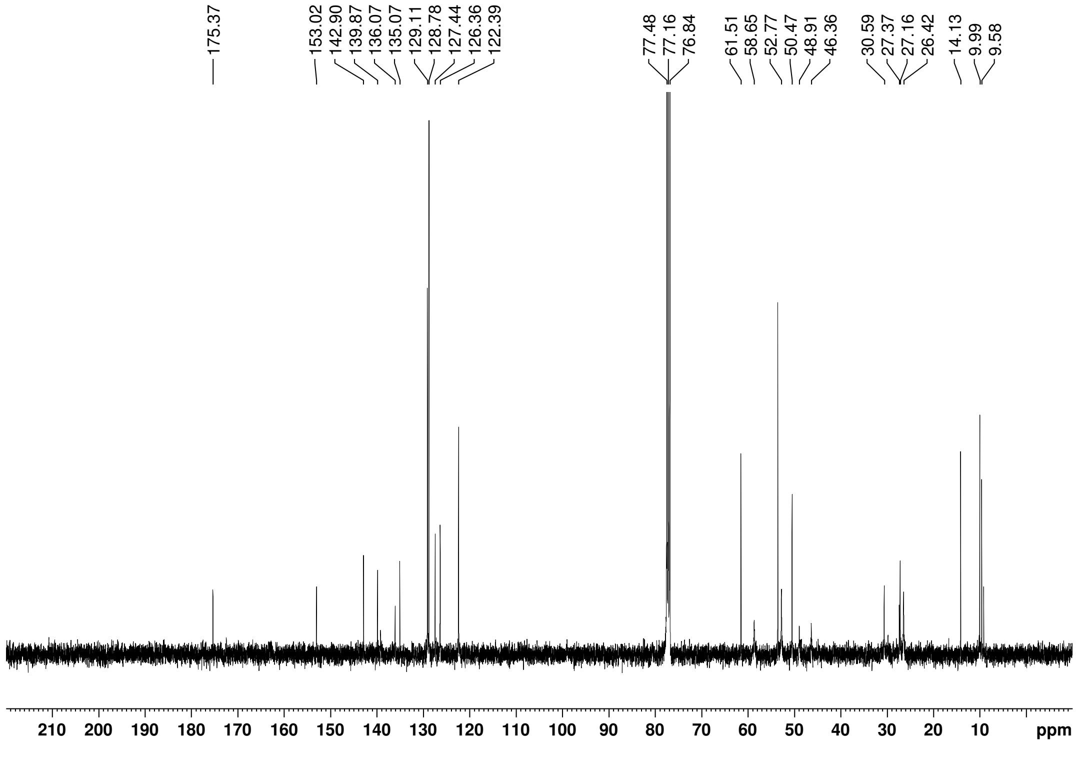

*Contains DCM

^{1}H spectrum of **24** (400 MHz, $CDCl_3$):

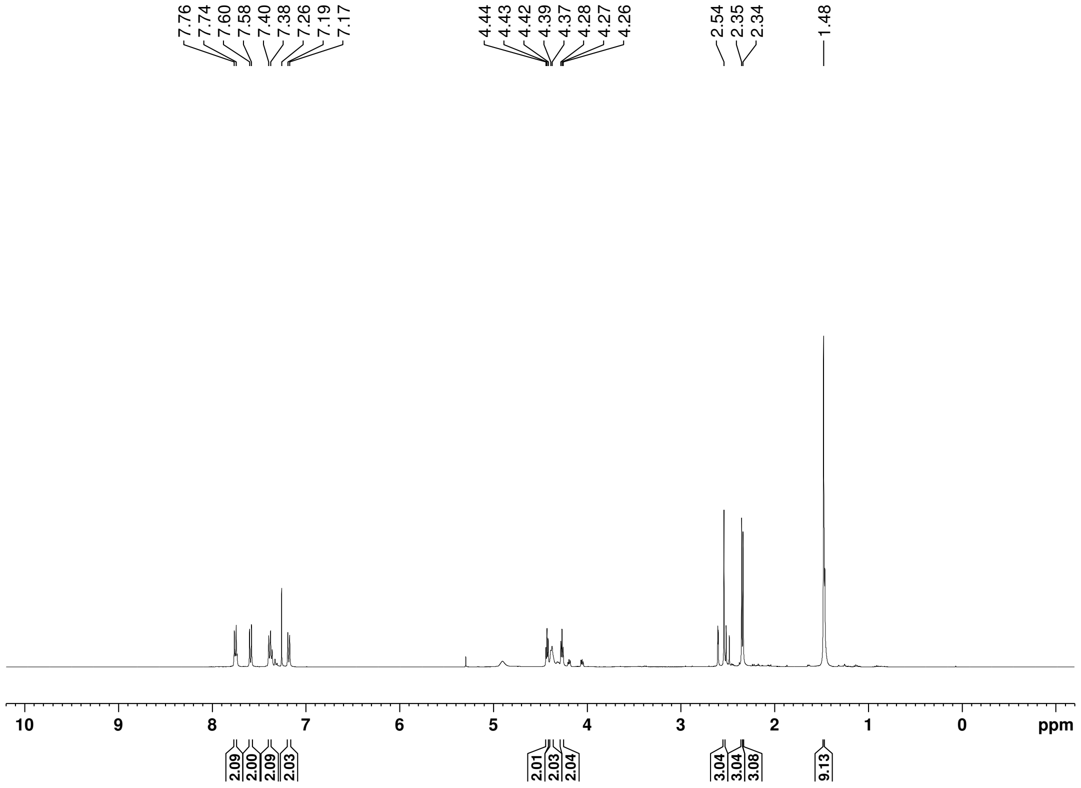

^{13}C spectrum of **24** (101 MHz, $CDCl_3$):

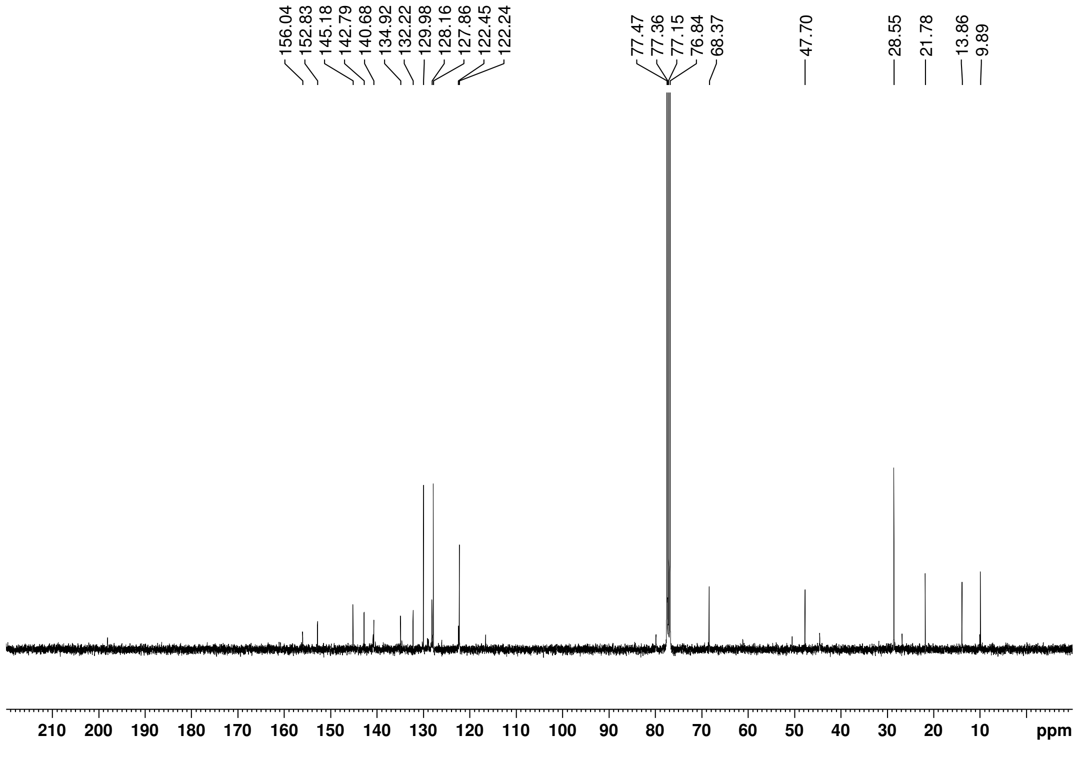

^{1}H spectrum of **25** (400 MHz, $CDCl_3$):

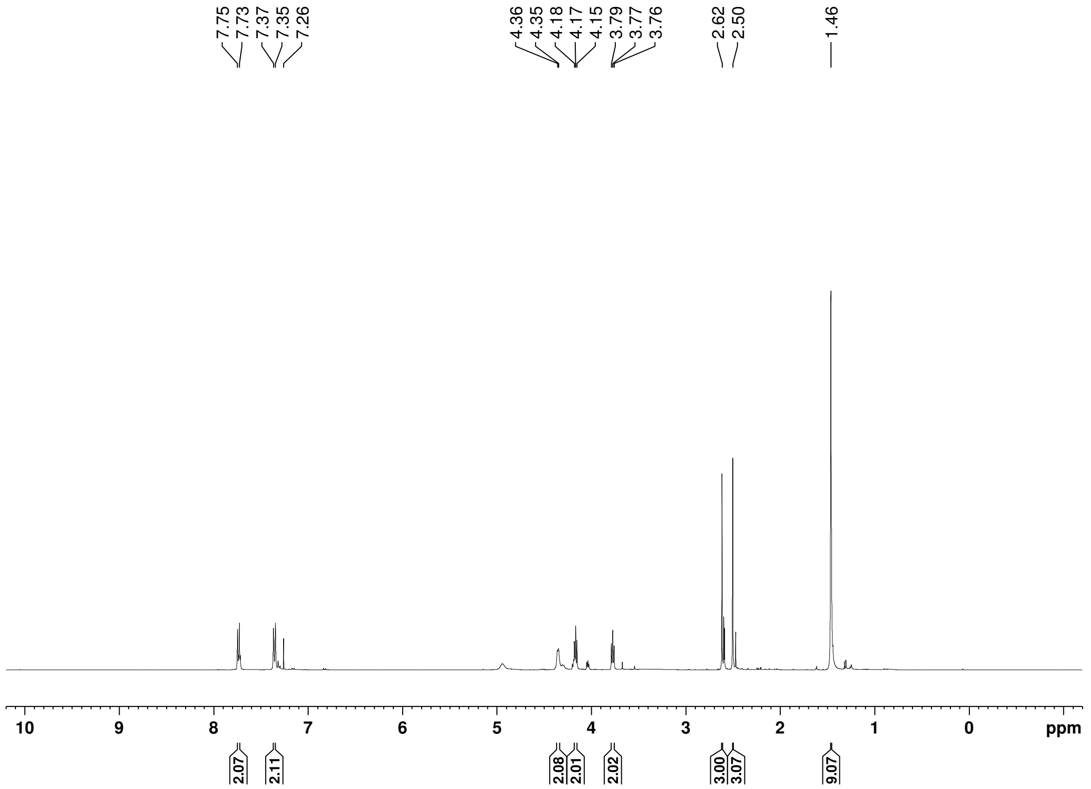

^{13}C spectrum of **25** (101 MHz, $CDCl_3$):

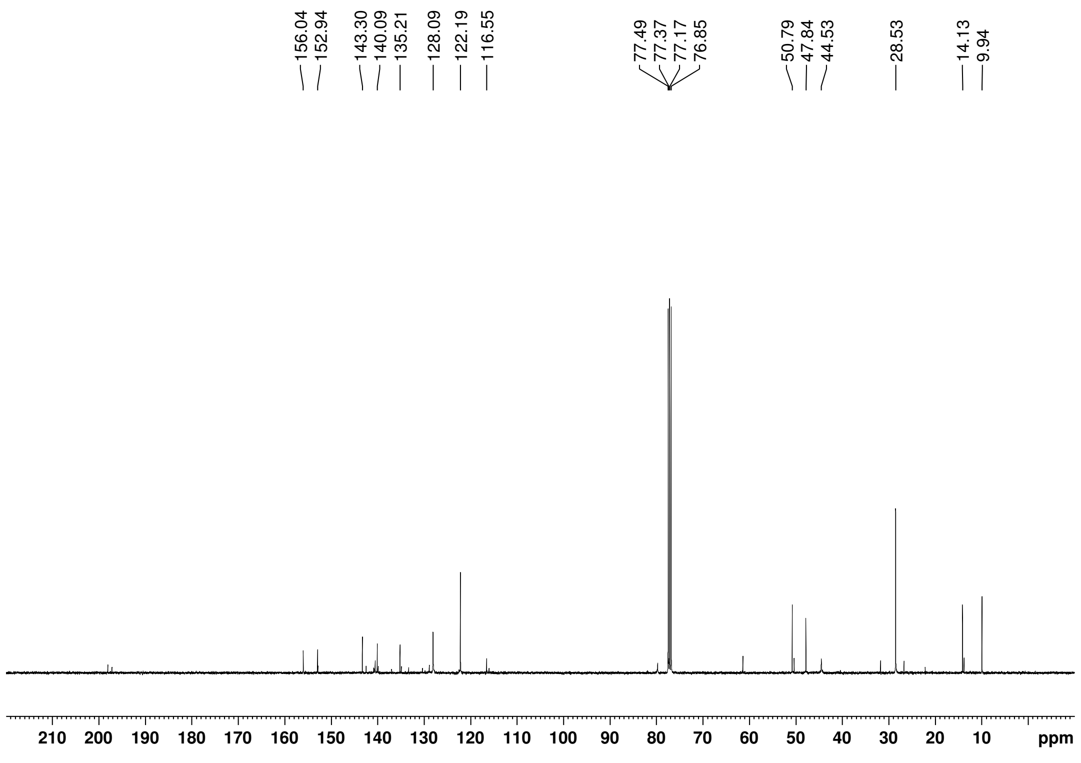

^{1}H spectrum of **26** (400 MHz, $CDCl_3$):

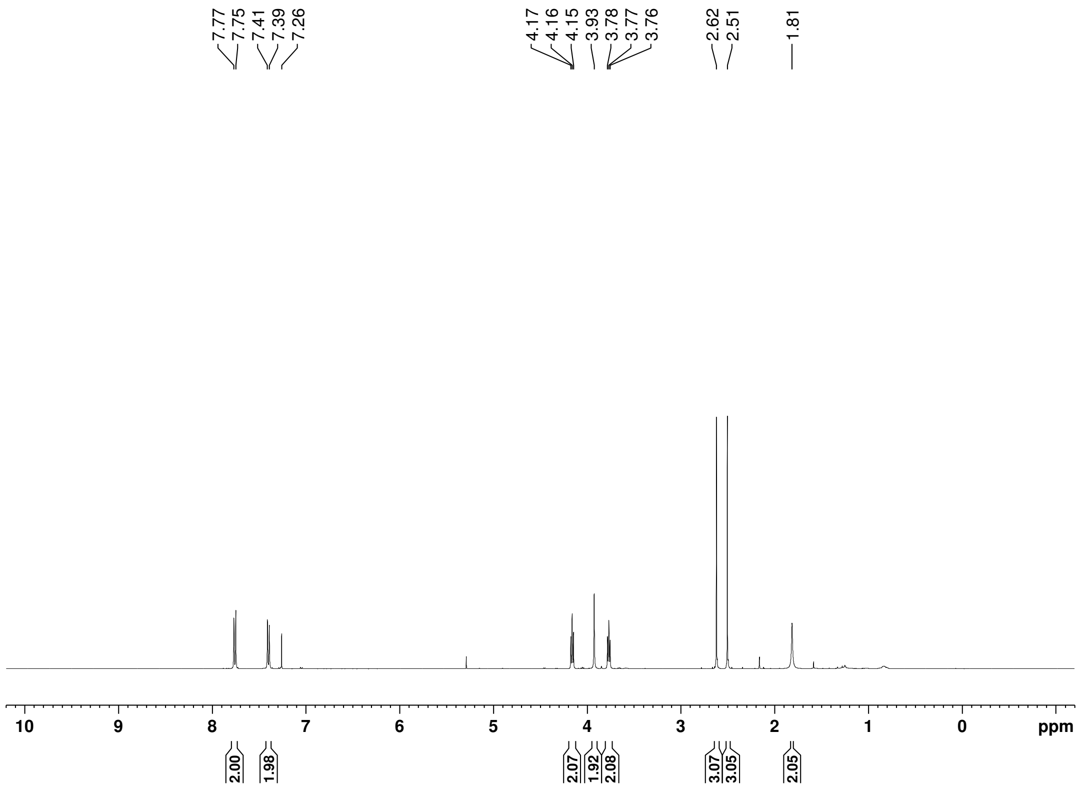

^{13}C spectrum of **26** (101 MHz, $CDCl_3$):

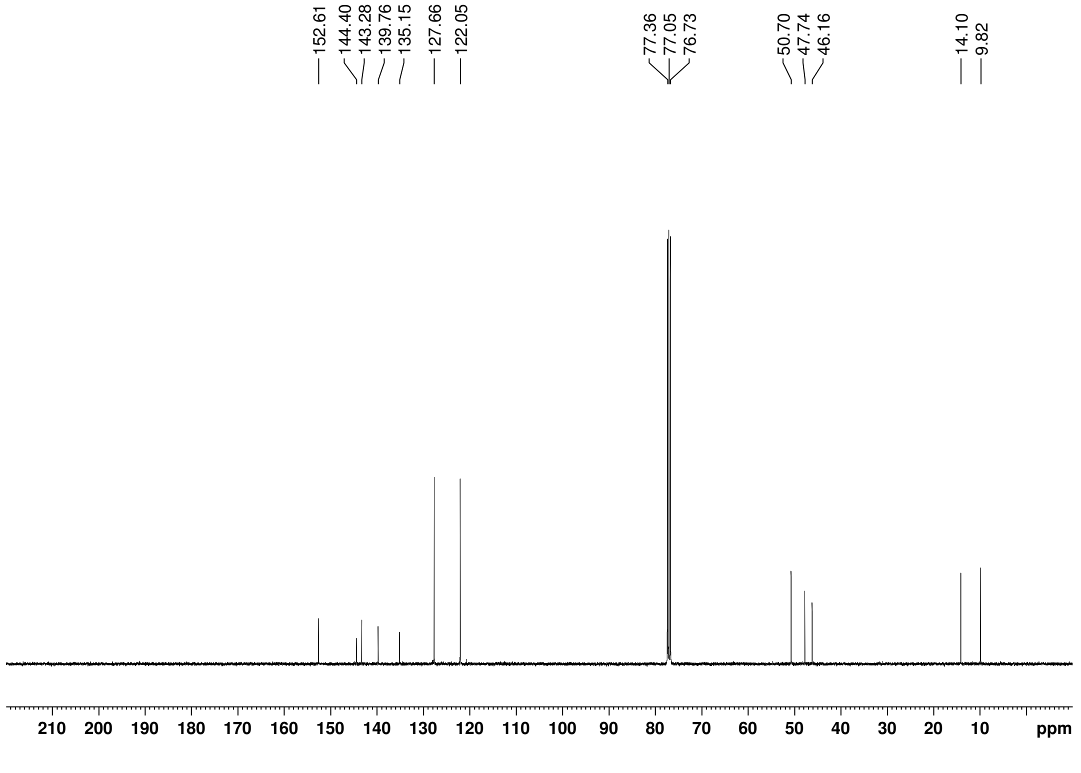

^{1}H spectrum of **27** (400 MHz, $CDCl_3$):

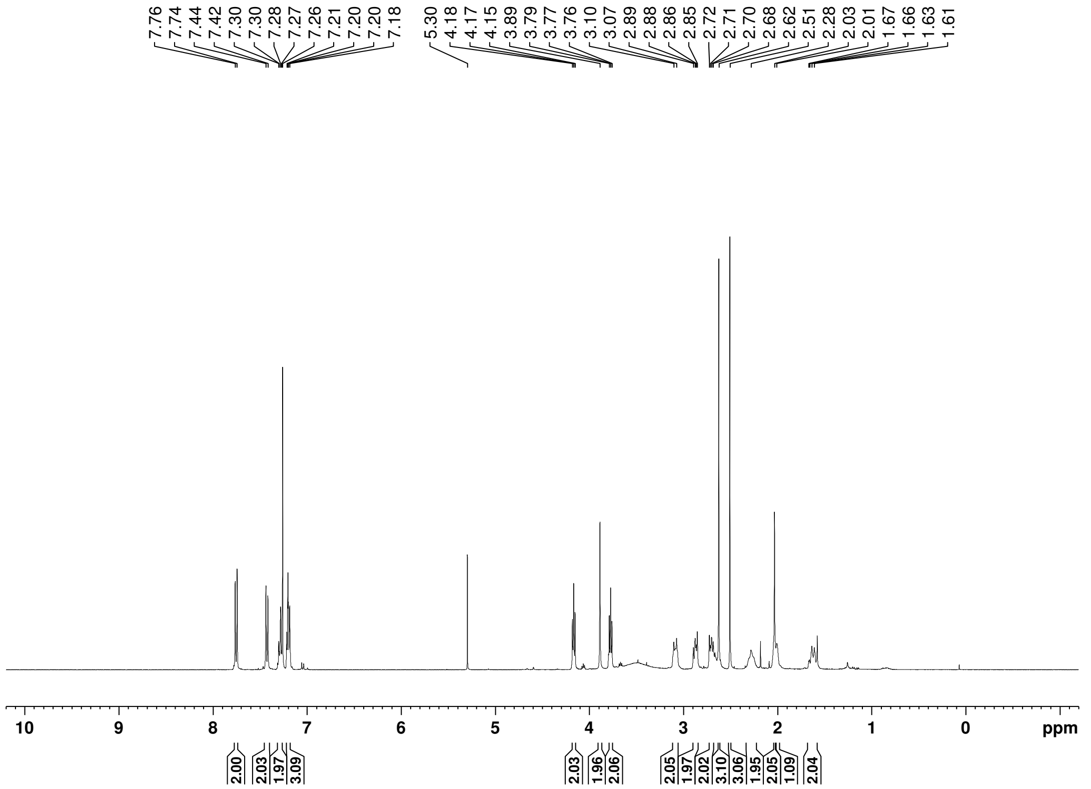

*Contains dichloromethane.

^{13}C spectrum of **27** (101 MHz, $CDCl_3$):

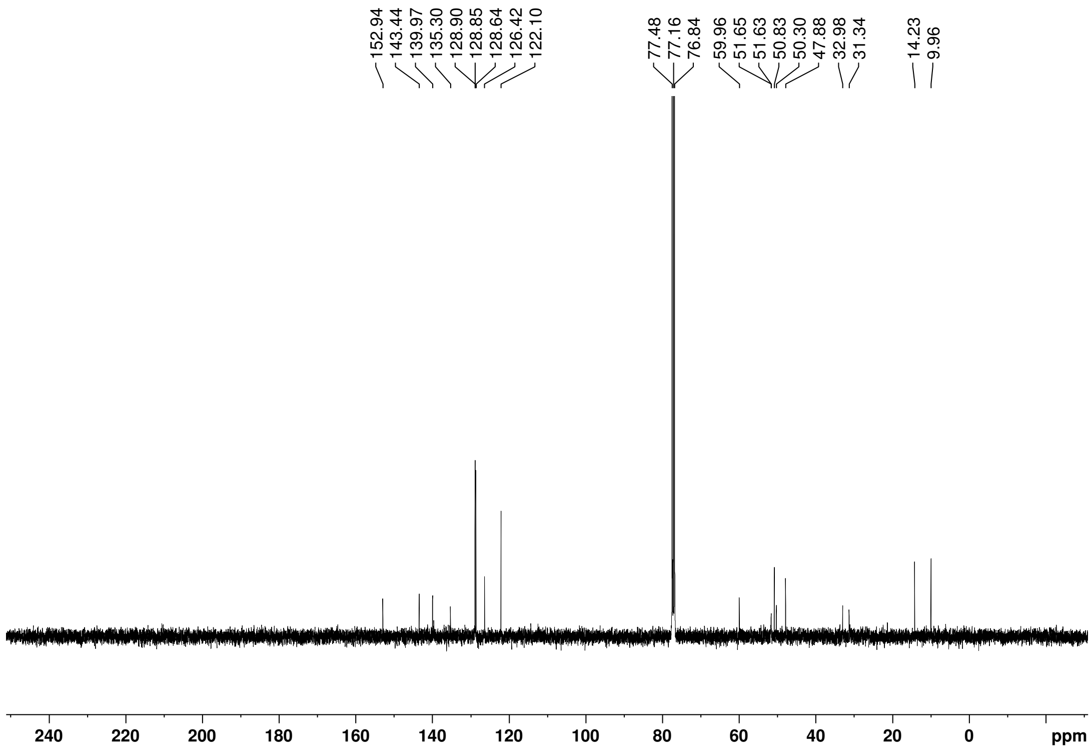

^{1}H spectrum of **3c** (400 MHz, $CDCl_3$):

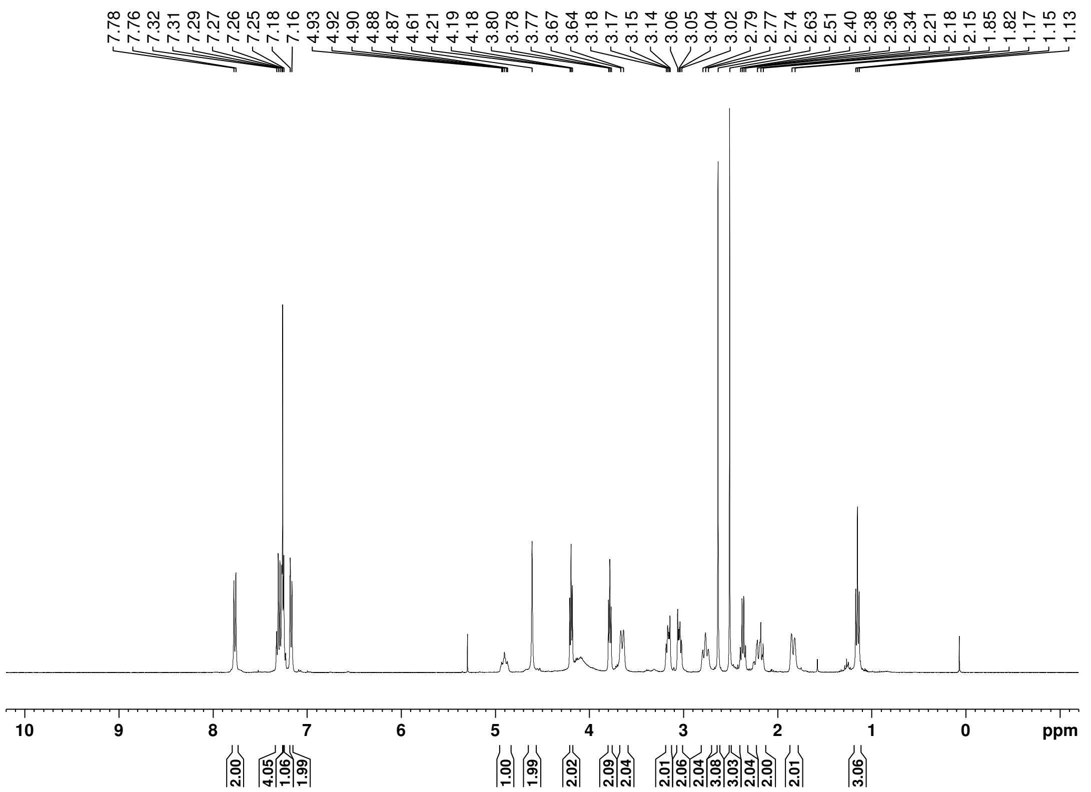

^{13}C spectrum of **3c** (101 MHz, $CDCl_3$):

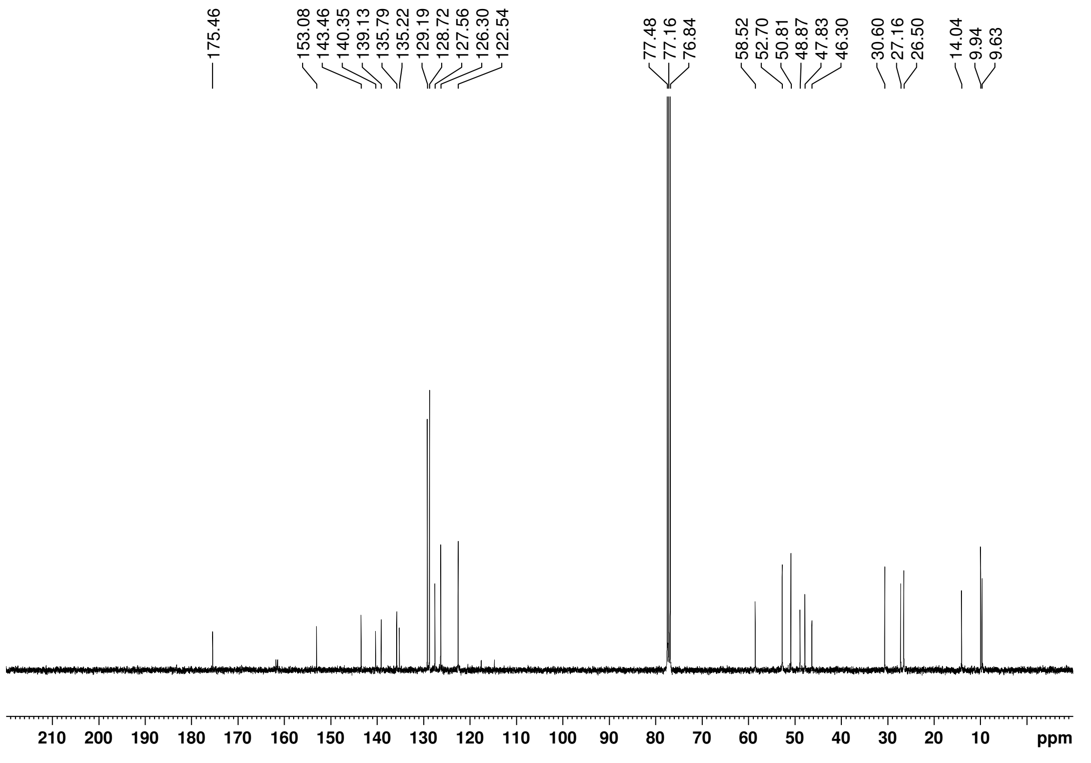

IR spectrum:

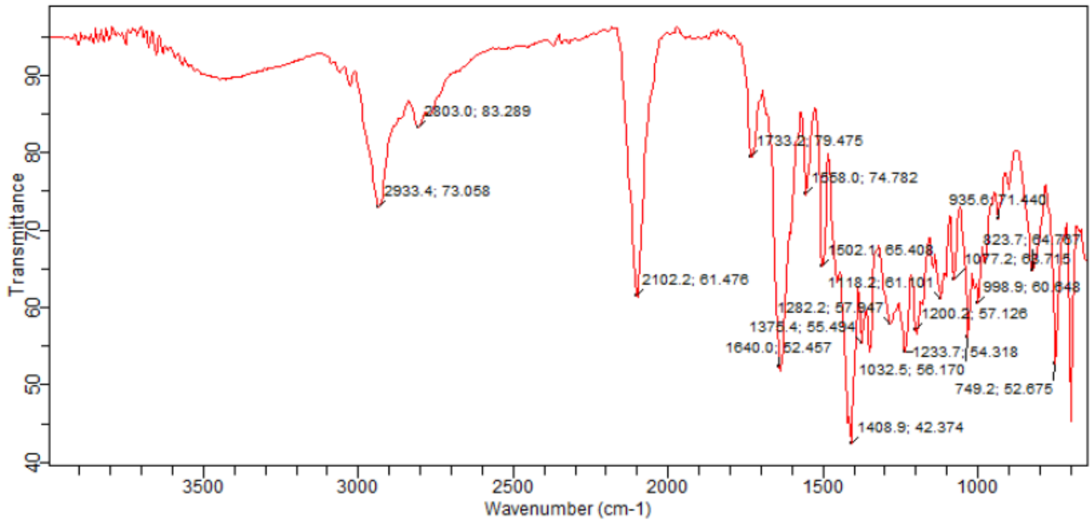

Transmittance
Wavenumber (cm-1)
2803.0; 83.289
2933.4; 73.058
2102.2; 61.476
1733.2; 79.475
1558.0; 74.782
1502.1; 65.408
1640.0; 52.457
1408.9; 42.374
935.6; 71.440
823.7; 64.767
749.2; 52.675

^{1}H spectrum of **30** (400 MHz, $CDCl_3$):

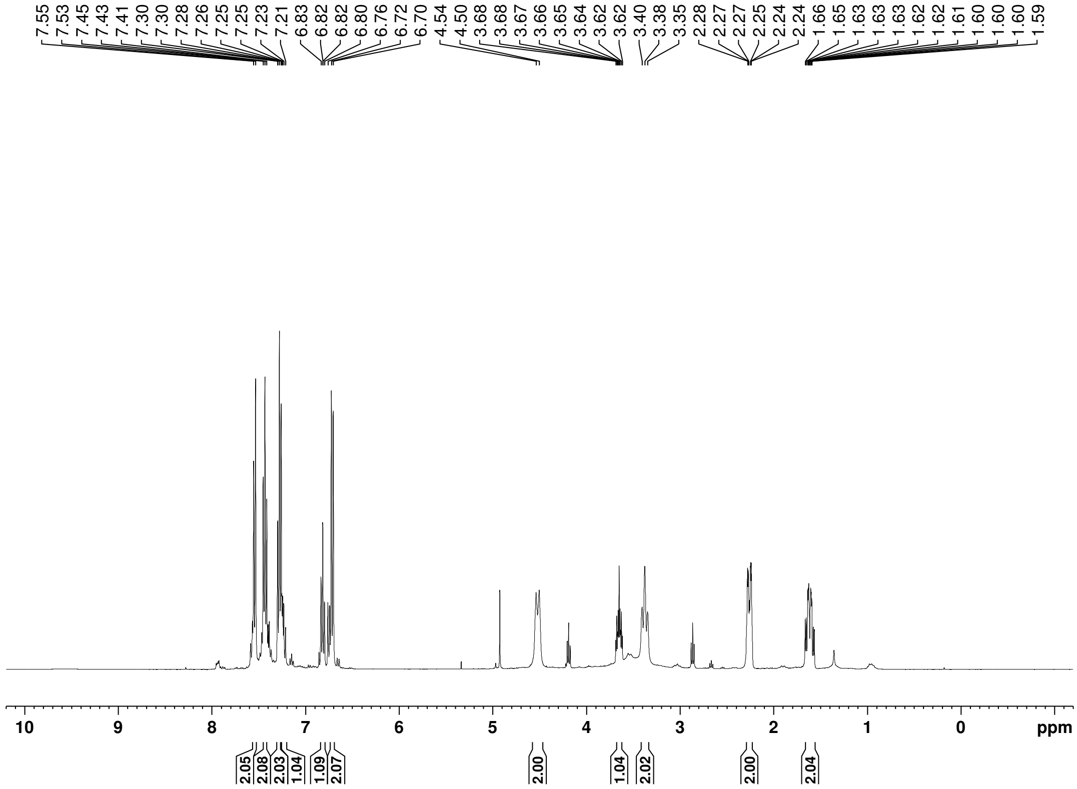

^{13}C spectrum of **30** (101 MHz, $CDCl_3$):

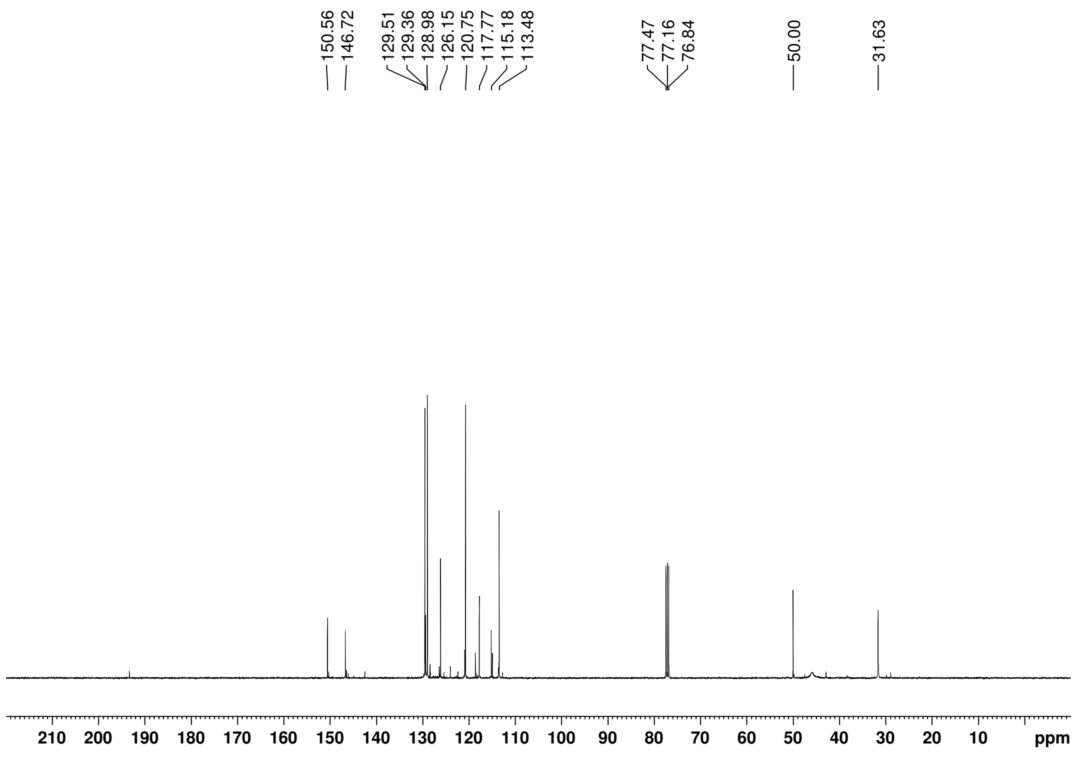

^{1}H spectrum of **4** (400 MHz, $CDCl_3$):

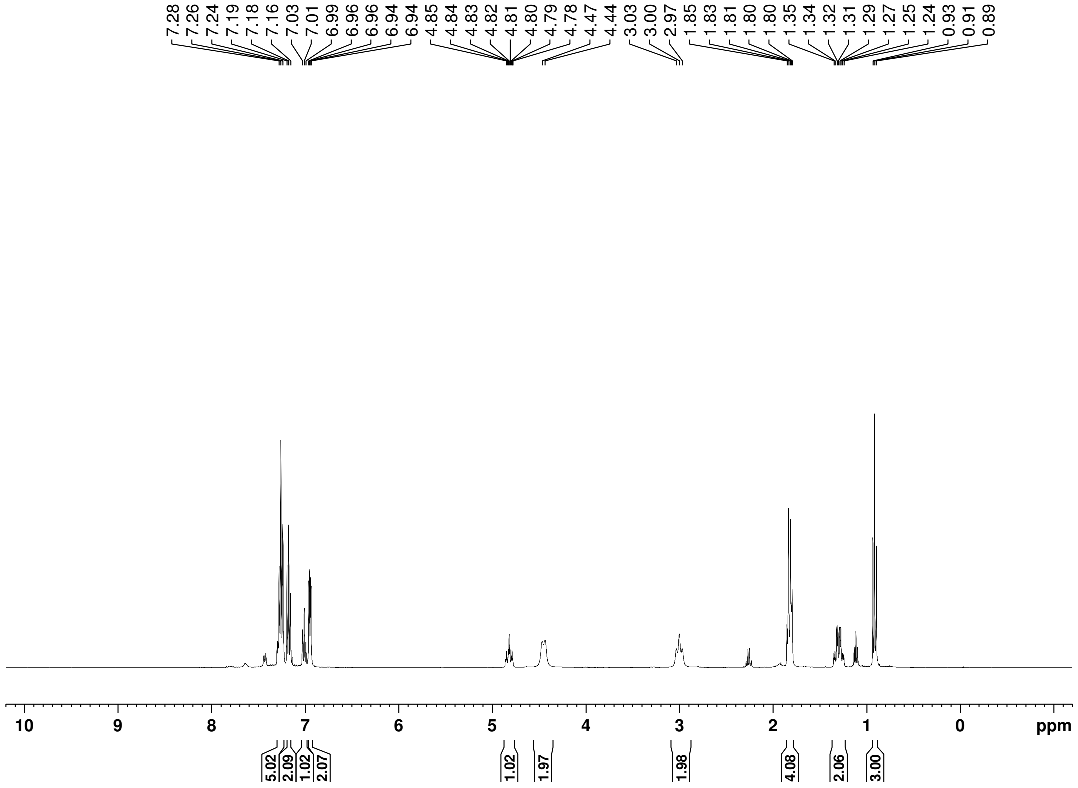

^{13}C spectrum of **4** (101 MHz, $CDCl_3$):

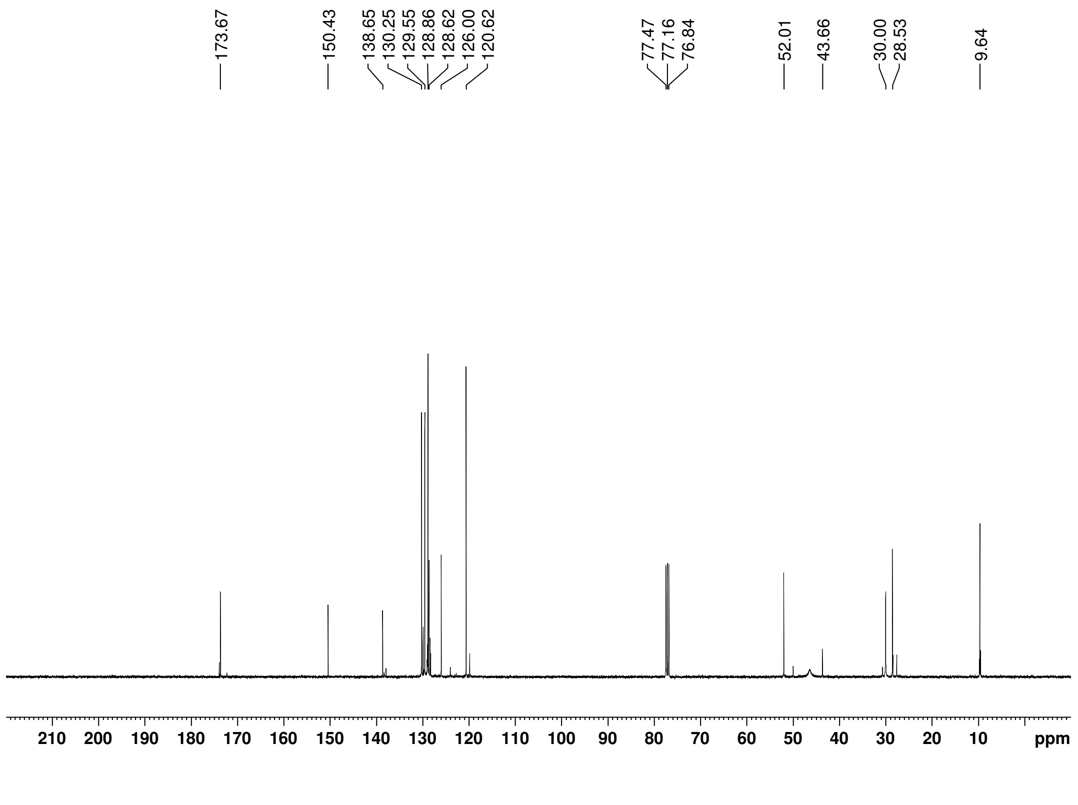

6. References

[1] W. Huang, A. Manglik, A. J. Venkatakrishnan, T. Laeremans, E. N. Feinberg, A. L. Sanborn, H. E. Kato, K. E. Livingston, T. S. Thorsen, R. C. Kling, S. Granier, P. Gmeiner, S. M. Husbands, J. R. Traynor, W. I. Weis, J. Steyaert, R. O. Dror, B. K. Kobilka, *Nature* **2015**, *524*, 315-321.

[2] L. Stricker, M. Böckmann, T. M. Kirse, N. L. Doltsinis, B. J. Ravoo, *Chem. Eur. J.* **2018**, *24*, 8639-8647.

[3] M. Schönberger, D. Trauner, *Angew. Chem. Int. Ed. Engl.* **2014**, *53*, 3264-3267.

[4] A. F. Casy, M. R. Huckstep, *J. Pharm. Pharmacol.* **1988**, *40*, 605-608.

[5] C. A. Valdez, R. N. Leif, B. P. Mayer, *PLoS One* **2014**, *9*, e108250.

[6] R. Lazny, M. Sienkiewicz, S. Bräse, *Tetrahedron* **2001**, *57*, 5825-5832.

[7] a) H. Hübner, C. Haubmann, W. Utz, P. Gmeiner, *J. Med. Chem.* **2000**, *43*, 756-762; b) A. Drakopoulos, Z. Koszegi, Y. Lanoiselée, H. Hübner, P. Gmeiner, D. Calebiro, M. Decker, *J. Med. Chem.* **2020**, *63*, 3596-3609.

[8] O. H. Lowry, N. J. Rosebrough, A. L. Farr, R. J. Randall, *J Biol Chem* **1951**, *193*, 265-275.

[9] Y. C. Cheng, W. H. Prusoff, *Biochem. Pharmacol.* **1973**, *22*, 3099-3108.

[10] a) H. Liu, J. Hofmann, I. Fish, B. Schaake, K. Eitel, A. Bartuschat, J. Kaindl, H. Rampp, A. Banerjee, H. Hübner, M. J. Clark, S. G. Vincent, J. T. Fisher, M. R. Heinrich, K. Hirata, X. Liu, R. K. Sunahara, B. K. Shoichet, B. K. Kobilka, P. Gmeiner, *Proc. Natl. Acad. Sci.* **2018**, *115*, 12046-12050; b) C. Gentzsch, K. Seier, A. Drakopoulos, M.-L. Jobin, Y. Lanoiselée, Z. Koszegi, D. Maurel, R. Sounier, H. Hübner, P. Gmeiner, S. Granier, D. Calebiro, M. Decker, *Angew. Chem. Int. Ed. Engl.* **2020**, *59*, 5958-5964.
